# General Method of Synthesis of 5-(Het)arylamino-1,2,3-triazoles via Buchwald–Hartwig Reaction of 5-Amino- or 5-Halo-1,2,3-triazoles

**DOI:** 10.3390/molecules27061999

**Published:** 2022-03-20

**Authors:** Pavel S. Gribanov, Anna N. Philippova, Maxim A. Topchiy, Lidiya I. Minaeva, Andrey F. Asachenko, Sergey N. Osipov

**Affiliations:** 1 A. N. Nesmeyanov Institute of Organoelement Compounds, Russian Academy of Sciences, 28 Vavilova Str., 119991 Moscow, Russia; anyfil96@gmail.com (A.N.P.); osipov@ineos.ac.ru (S.N.O.); 2 A.V. Topchiev Institute of Petrochemical Synthesis, Russian Academy of Sciences, Leninskiy Prospect 29, 119991 Moscow, Russia; maxtopchiy@yandex.ru (M.A.T.); minaeva.lidiya@gmail.com (L.I.M.); aasachenko@gmail.com (A.F.A.)

**Keywords:** cross-coupling, amination, triazoles, palladium, carbene ligands, heterocycles

## Abstract

An efficient access to the novel 5-(het)arylamino-1,2,3-triazole derivatives has been developed. The method is based on Buchwald–Hartwig cross-coupling reaction of 5-Amino or 5-Halo-1,2,3-triazoles with (het)aryl halides and amines, respectively. As result, it was found that palladium complex [(THP-Dipp)Pd(cinn)Cl] bearing expanded-ring *N*-heterocyclic carbene ligand is the most active catalyst for the process to afford the target molecules in high yields.

## 1. Introduction

**Figure 1:**
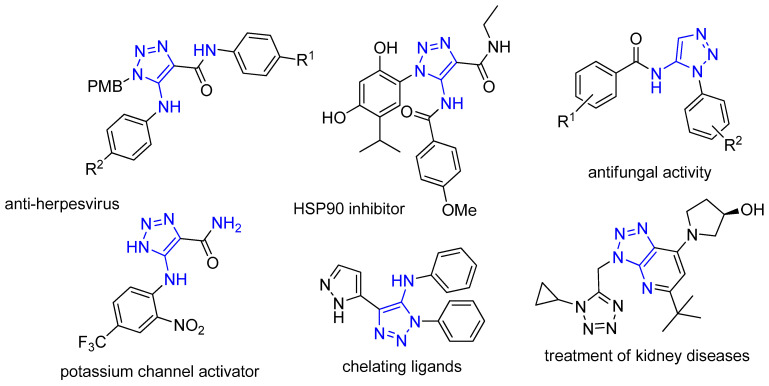
Potential application of 5-amino-1,2,3-triazoles *N*-substituted derivatives.

Nitrogen containing heterocycles, in particular five-membered azole systems, are common structural elements of many natural and synthetic biological active compounds. They serve as universal scaffolds for creating new organic molecules with set properties especially for the needs of biomolecular and medicinal chemistry as well as for materials science [1,2,3,4,5,6]. In the last few decades fully substituted and variously functionalized 1,2,3-triazoles, whose structure fragment is not found in nature, became one of the most interesting and widely used class of compounds due to their unique physicochemical properties and synthetic accessibility [7,8]. These compounds possess remarkable thermal and metabolic stability, large dipole moment, and capability for H-bond formation making them effective peptide bond isosteres [9,10,11] that result in a variety of applications in diverse fields of chemistry [12,13,14,15,16,17,18,19,20]. Among fully substituted 1,2,3-triazoles special attention is focused on 5-amino-1,2,3-triazoles and their 5-arylamino derivatives, which exhibit very promising biological properties such as antiviral, antifungal, antiproliferative and antimetastatic activities. They also serve as activators of potassium channel andchelating agents and have a potential for treating inflammatory kidney diseases (Figure 1) [21,22,23,24,25,26].

**Scheme 1:**
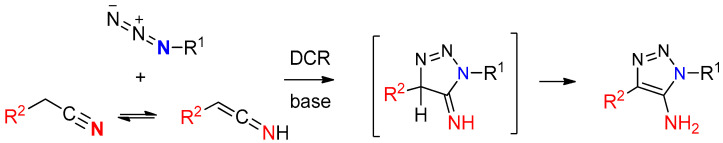
1,3-Dipolar cycloaddition reaction (DCR) between aryl azides and monosubstituted acetonitriles.

Since the pioneering Dimroth works published in the beginning of the 20th century [27,28], keteniminate-mediated 1,3-dipolar cycloaddition (DCR) of organic azides with nitriles bearing an active methylene group provide one of the most efficient and straightforward methods to access to the 5-amino-1,2,3-triazole synthesis up to date (Scheme 1) [29,30,31,32]. 

Unfortunately this approach is not applicable to 5-amino substituted 1,2,3-triazoles including 5-arylamino derivatives. The scope of the existing methods for the synthesis of these compounds is limited to a few examples and has a number of disadvantages. Thus, previously described methods for the preparation of 5-arylamino-1,2,3-triazoles include: (1) interaction between hard accessible carbodiimides and diazo compounds [21]; (2) three-component amine/enolizible ketone/azide reaction leading to low yields of the target products [33]; (3) high temperature thermolysis of the 5-triazenyl-1,2,3-triazoles to give a large amount of 2H-1,2,3-triazole as a by-product [34]; (4) base-mediated hydrolysis of 1,2,3-triazolo[1,5-a]quinazolin-5(4H)-ones [35] as well as Rh-catalyzed azide-alkyne cycloaddition of internal ynamides to afford *N*,*N*-disubstituted amino-1,2,3-triazoles [26].

**Figure 2:**
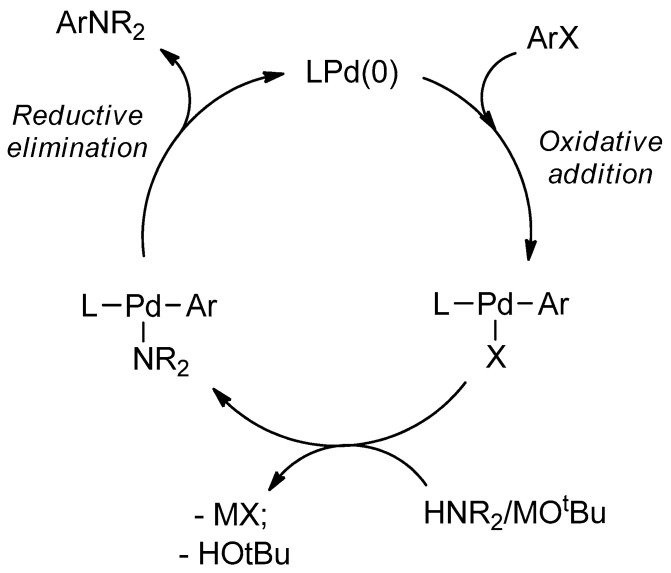
Simplified catalytic cycle for Buchwald−Hartwig amination reaction.

On the other hand, in the past 30 years, palladium-catalyzed cross-coupling reactions leading to the formation of new C-N bonds have become a widely used tool both in academia and in industry [36,37]. This Buchwald–Hartwig amination is the most popular cross-coupling reaction [38,39,40] (Figure 2) to access a wide range of *N*-mono- and *N,N*-disubstituted arylamines [41]. Despite impressive advances in the field, coupling of heteroaromatic amines with (het)aryl halides still remains problematic, often requiring long reaction times and time-consuming searches for optimal conditions and catalytic systems [3,42,43,44,45]. tThere are no examples of Buchwald–Hartwig cross-coupling of 5-halo- and 5-amino-1,2,3-triazoles with (het)aryl amines and halides, respectively, to afford *N*-aryl amino derivatives except a report on synthesis of related 4-amino-1,2,3-triazoles (with just 3 examples) [46].

Therefore, taking into account the growing popularity of 5-amino-1,2,3-triazole derivatives in medical chemistry, the development of new efficient and robust approaches to their synthesis remains of great interest.

**Scheme 2:**
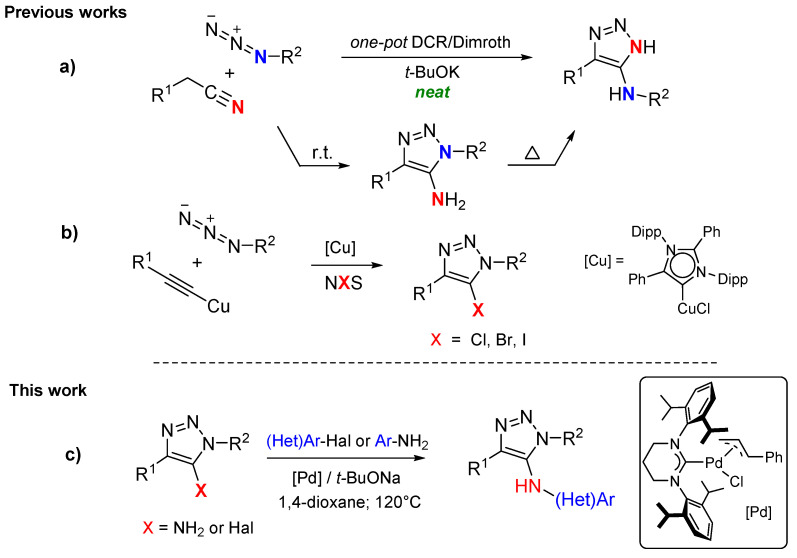
Synthetic approach to 5-amino-1,2,3-triazoles (**a**), 5-halo-1,2,3-triazoles (**b**) and *N*-arylamino 1,2,3-triazoles (**c**).

We have recently developed effective methods for obtaining 5-amino- [47] and 5-halo-1,2,3-triazoles [48] via one pot azide-nitrile cycloaddition/Dimroth rearrangement (Scheme 2a) and Cu(I)-catalyzed [3+2] cycloaddition reaction of Cu(I)-acetylide and aryl azides with subsequent Cu-triazolide halogenation (Scheme 2b). Based on our experience in Pd-catalyzed cross-couplings of hetaryl halides [49,50,51,52] and halo-1,2,3-triazoles [53,54] we would like to provide details of an efficient route to *N*-arylamino-1,2,3- triazoles using the Buchwald–Hartwig reaction of 5-amino or 5-halo-1,2,3-triazoles (Scheme 2c).

## 2. Results and Discussion

**Figure 3:**
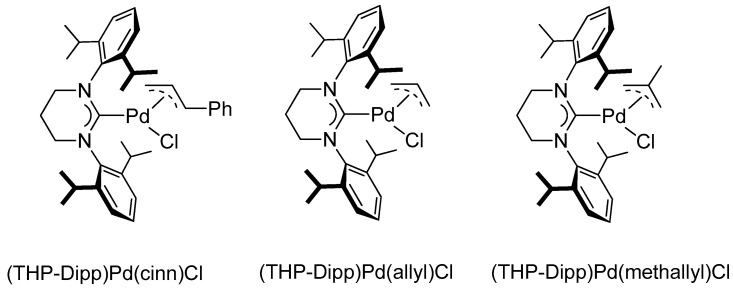
Structures of (THP-Dipp) Pd complexes.

We commenced our investigation with the reaction between 1-benzyl-4-phenyl-1,2,3-triazole-5-amine and 1-bromo-4-methylbenzene to screen for optimal conditions for the cross-coupling (Table 1). A series of palladium complexes with expanded-ring NHC ligands (Figure 3) were initially tested as they proved to be competent catalysts for Buchwald–Hartwig amination of (het)aryl halides with primary aryl amines [50,51]. We found that the reaction performed in the presence of 1.0 mol% (THP-Dipp)Pd(cinn)Cl and 1.2 equiv. of sodium *tert*-butoxide in 1,4-dioxane at 120 °C for 24 h yielded the desired 5-(*p*-tolyl)amino-1,2,3-triazole **2a** in 53% yield (Table 1, entry 1). The reaction did not reveal the full conversion of the starting materials (TLC and ^1^H NMR analysis). The prolonged reaction time did not result in a better yield of the product. The increase of the Pd-catalyst loading up to 2 mol% and the base up to 3.0 equiv. almost led to quantitative formation of **2a** (entry 3). Other NHC-Pd complexes with allyl and metallyl ligands exhibited slightly less activity under tested conditions (entries 4, 5). The traditional Pd(OAc)_2_/phosphine-based catalytic systems [55] were also tested, exhibiting insufficient activity for the process (entries 6–9).

**Scheme 3:**
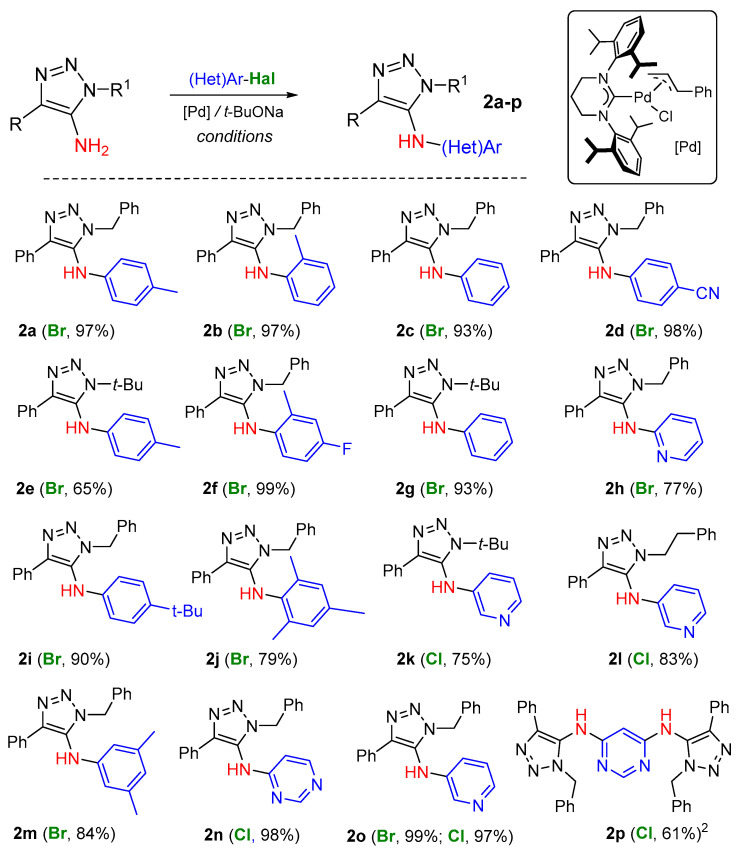
Buchwald–Hartwig cross-coupling of 5-amino-1,2,3-triazoles ^1^. ^1^ Conditions: 5-amino-1,2,3-triazole (0.5 mmol); (het)aryl-Hal (1 equiv.); (THP-Dipp)Pd(cinn)Cl (2 mol %); *t*-BuONa (3 equiv.); 1,4-dioxane (2.5 mL); 120 °C under argon 24 h; ^2^ 4,6-Dichloropyrimidine (0.25 mmol); 5-aminotriazole (2.0 equiv.); (THP-Dipp)Pd(cinn)Cl (4 mol %), *t*-BuONa (6 equiv.).

With these optimized conditions in hand, different 5-amino-1,2,3-triazoles were involved in the Buchwald–Hartwig cross-coupling reactions with a wide range of aromatic and heteroaromatic halides bearing various substituents in their structures. As a result, we found that in all studied cases the nature and location of the substituent in the (het)aryl core of both triazole and halide substrates doesn’t not significantly influence the reaction leading to the formation of the corresponding 5-amino-1,2,3-triazoles derivatives **2a**–**p** including sterically hindered *ortho*-Me aryl derivatives **2b**, **2f**, **2j** in good and excellent yields. It is noteworthy that the reaction works perfectly for both (het)aryl bromides and chlorides (Scheme 3).

**Scheme 4:**
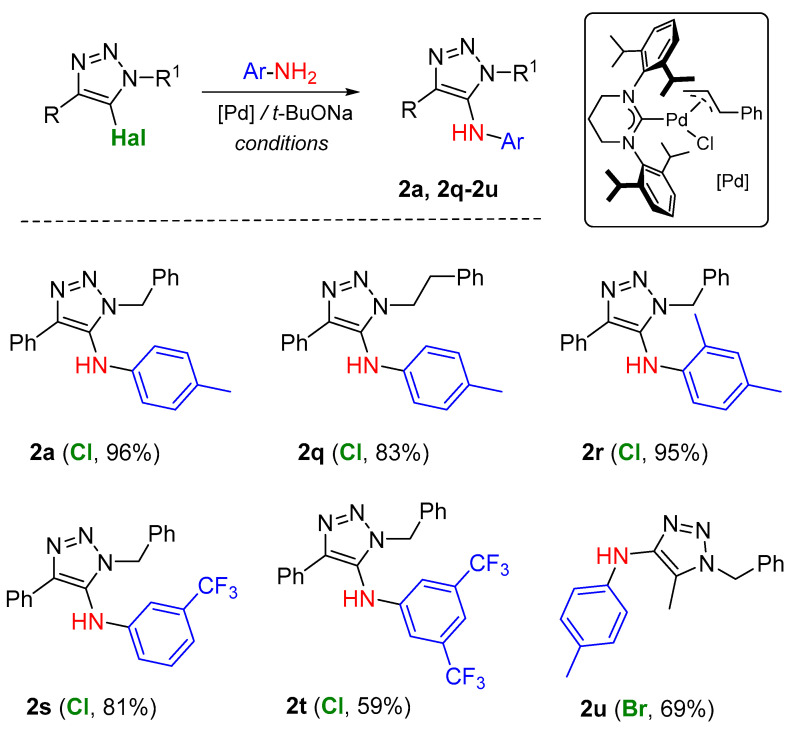
Buchwald–Hartwig cross-coupling of 4- and 5-halo-1,2,3-triazoles ^1^. ^1^ Reaction conditions: 4- or 5-halo-1,2,3-triazole (0.5 mmol); aryl-NH_2_ (1 equiv.); (THP-Dipp)Pd(cinn)Cl (2 mol %); *t*-BuONa (3 equiv.); 1,4-dioxane (2.5 mL); 120 °C under argon, 24 h.

Then, we studied the reversed variant of the Buchwald–Hartwig cross-coupling reaction, namely the interaction of 5-halo-1,2,3-triazoles with aryl amines. Fortunately, we found that the conditions for aminotriazole—aryl halide coupling proved to also be suitable for the combination of halotriazole—aryl amine. Thus, corresponding derivatives of 5-arylamino-1,2,3-triazole such as *N*-(*p*-tolylamino) (**2a**, **2q**) and *N*-(2,4-dimethylamino) (**2r**) triazoles were obtained in good to excellent yields. Arylamines with electron-withdrawing CF_3_ group(s) in aromatic ring (**2s** and **2t**) can also be successfully used for this reaction. Example **2u** demonstrates that the method is also applicable for the preparation of 4-(*N*-arylamino)-1,2,3-triazoles from the corresponding 4-halo-1,2,3-triazoles, while their synthesis was previously described via coupling of 4-amino-1,2,3-triazoles [46] (Scheme 4).

## 3. Materials and Methods

### 3.1. General Information

All the reactions were carried out under argon atmosphere, and the solvents were distilled from appropriate drying agents prior to use. All reagents were used as purchased from Sigma-Aldrich (Munich, Germany). In the study, 1,4-disubstituted-5-chloro- [48] and 5-amino-1,2,3-triazoles [47] and 1-benzyl-4-bromo-5-methyl-1*H*-1,2,3-triazole [56] were synthesized according to published procedures. (THP-Dipp)Pd(cinn)Cl [57], (THP-Dipp)Pd(allyl)Cl [58] and (THP-Dipp)Pd(metallyl)Cl were synthesized according to published procedure [57] from corresponding NHC-silver (I) complexes. Analytical data was in accordance with the literature data. Analytical TLC was performed with Merck silica gel 60 F 254 plates (Darmstadt, Germany); visualization was accomplished with UV light or iodine vapors. Chromatography was carried out using Merck silica gel (Kieselgel 60, 0.063–0.200 mm, Darmstadt, Germany) and petroleum ether/ethyl acetate as an eluent. The NMR spectra were obtained with Bruker AV-400, Karlsruhe, Germany) (400 MHz ^1^H, 101 MHz ^13^C, 376 MHz ^19^F) using TMS and CCl_3_F as references for ^1^H and ^19^F NMR spectra. respectively. Chemical shifts for ^1^H and ^13^C were reported as δ values (ppm).

### 3.2. General Procedure for Preparation of N-arylamino-1,2,3-triazoles via BHA Reaction of 5-Amino or 4(5)-halo-1,2,3-triazoles

Under argon in a Schlenk tube with magnetic stirring bar, corresponding amino- or halo-1,2,3-triazole (0. 5 mmol), (het)arylhalide or primary amine (1.0 equiv.) were dissolved in dry 1,4-dioxane (2.5 mL) at room temperature. The solution was degassed with three freeze-pump-thaw cycles. Then 6.6 mg (0.01 mmol, 2 mol%) of (THP-Dipp)Pd(cinn)Cl and sodium *tert*-butoxide (3.0 equiv.) were added to the reaction mixture, and the reaction mixture was stirred at 120 °C (oil bath temperature) for 18 h. After cooling to room temperature, the reaction mixture was poured into water and extracted with dichloromethane (3 × 10 mL). The combined organic phases were washed with brine, dried over MgSO_4_, filtered and concentrated under reduced pressure. Purification by chromatography (eluent—hexane: ethyl acetate 4:1) gave analytically pure corresponding *N*-arylamino-1,2,3-triazole as a white solid.

### 3.3. Preparation and Characterization of Novel Compounds

*(THP-Dipp)Pd(methallyl)Cl*

The title compound was synthesized according to literature procedure [58] from (6-Dipp)AgBr and (2-Methylallyl)palladium(II) chloride dimer as a white powder (88% yield). ^1^H NMR (400 MHz, Acetone-*d*_6_) δ 7.40–7.11 (m, 6H), 3.85–3.56 (m, 7H), 3.33–3.18 (m, 2H), 2.88 (s, 1H), 2.67–2.59 (m, 2H), 2.53–2.37 (m, 2H), 1.51–1.15 (m, 24H), 1.02 (s, 2H). ^13^C DEPTQ-135 NMR (Acetone, 101 MHz): δ 214.8, 146.6, 143.8, 130.0, 129.1, 128.2, 125.5, 70.3, 50.3, 49.2, 47.0, 47.0, 29.1, 27.1, 25.2, 24.9, 22.9, 22.1, 21.2, 21.0. HRMS (ESI): calcd for C_32_H_47_N_2_Pd [(THP-Dipp)Pd(methallyl)]^+^: 563.2775, 564.2788, 565.2781, 566.2808, 567.2777; found: 563.2776, 564.2795, 565.2788, 566.2810, 567.2780.

*1-benzyl-5-(p-tolylamino)-4-phenyl-1H-1,2,3-triazole* (**2a**)

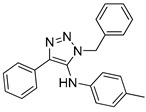



From 1-benzyl-4-phenyl-1*H*-1,2,3-triazol-5-amine and 1-bromo-4-methylbenzene (165 mg, 97% yield) or from 1-benzyl-5-chloro-4-phenyl-1*H*-1,2,3-triazole and *p*-toluidine (163 mg, 96% yield), following general procedure, **2a** was obtained as a white solid, m.p. 181–182 °C. ^1^H NMR (400 MHz, Chloroform-*d*) δ 7.82 (d, *J* = 7.0 Hz, 2H), 7.34–7.26 (m, 6H), 7.22–7.17 (m, 2H), 7.00 (d, *J* = 8.4 Hz, 2H), 6.45 (d, *J* = 8.4 Hz, 2H), 5.36 (s, 2H), 5.05 (s, 1H), 2.27 (s, 3H). ^13^C{^1^H} NMR (101 MHz, Chloroform-*d*) δ 141.2, 140.9, 134.8, 132.1, 130.3, 130.0, 129.0, 128.7, 128.4, 128.0, 127.9, 126.0, 114.3, 51.4, 20.6. IR (υ/cm^−1^): 737.34 (VS), 812 (VS), 1006 (S), 1072 (S), 1177 (S), 1251 (S), 1288 (S), 1325 (S), 1359 (S), 1422 (S), 1441 (S), 1518 (S), 1586 (S), 1610 (S), 1810 (M), 1888 (M), 1955 (M), 2980 (W), 3025 (W), 3249 (M). HRMS (ESI): calcd for C_22_H_21_N_4_ [M+H]^+^: 341.1761; found: 341.1769.

*1-benzyl-5-(o-tolylamino)-4-phenyl-1H-1,2,3-triazole* (**2b**)

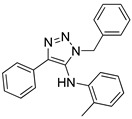



From 1-benzyl-4-phenyl-1*H*-1,2,3-triazol-5-amine and 1-bromo-2-methylbenzene, following general procedure, **2b** (165 mg, 97% yield) was obtained as a white solid, m.p. 193–195 °C. ^1^H NMR (400 MHz, Chloroform-*d*) δ 7.76 (d, *J* = 7.0 Hz, 2H), 7.33–7.25 (m, 6H), 7.18–7.12 (m, 3H), 6.98 (t, *J* = 7.5 Hz, 1H), 6.83 (t, *J* = 7.4 Hz, 1H), 6.25 (d, *J* = 8.1 Hz, 1H), 5.33 (s, 2H), 4.82 (s, 1H), 2.12 (s, 3H). ^13^C{^1^H} NMR (101 MHz, Chloroform-*d*) δ 141.5, 141.0, 134.7, 131.9, 131.1, 130.4, 129.0, 128.8, 128.5, 128.1, 127.8, 127.6, 125.9, 123.3, 120.7, 112.7, 51.8, 17.5. IR (υ/cm^−1^): 3271 (W), 1606 (S), 1586 (S), 1571 (S), 1514 (S), 1496 (S), 1448 (S), 1411 (S), 1362 (S), 1294 (S), 1251 (S), 1159 (S), 1110 (S), 1073 (S), 1006 (S), 769 (VS), 747 (VS), 734 (VS), 717 (VS). HRMS (ESI): calcd for C_22_H_21_N_4_ [M+H]^+^: 341.1761; found: 341.1764.

*1-benzyl-5-(phenylamino)-4-phenyl-1H-1,2,3-triazole* (**2c**)

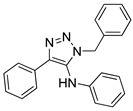



From 1-benzyl-4-phenyl-1*H*-1,2,3-triazol-5-amine and bromobenzene, following general procedure, **2c** (151 mg, 93% yield) was obtained as a white solid, m.p. 187–188 °C. ^1^H NMR (400 MHz, Chloroform-*d*) δ 7.80 (dd, *J* = 8.3, 1.4 Hz, 2H), 7.31–7.24 (m, 6H), 7.20–7.15 (m, 4H), 6.87 (t, *J* = 7.4 Hz, 1H), 6.52 (d, *J* = 7.6 Hz, 2H), 5.34 (s, 2H), 5.14 (s, 1H). ^13^C{^1^H} NMR (101 MHz, Chloroform-*d*) δ 143.6, 141.1, 134.7, 131.6, 130.2, 129.9, 129.0, 128.8, 128.5, 128.1, 127.9, 126.0, 120.7, 114.3, 51.5. IR (υ/cm^−1^): 3234 (M), 3180 (W), 2930 (W), 1602 (S), 1582 (S), 1568 (S), 1496 (S), 1445 (S), 1422 (S), 1364 (S), 1325 (S), 1256 (S), 1236 (S), 1176 (S), 1151 (S), 1077 (S), 770 (VS), 752 (VS). HRMS (ESI): calcd for C_21_H_19_N_4_ [M+H]^+^: 327.1604; found: 327.1608.

*1-benzyl-5-((4-benzonitrile)amino)-4-phenyl-1H-1,2,3-triazole* (**2d**)

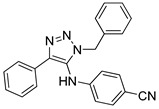



From 1-benzyl-4-phenyl-1*H*-1,2,3-triazol-5-amine and 4-bromobenzonitrile, following general procedure, **2d** (172 mg, 98% yield) was obtained as a white solid, m.p. 179–180 °C. ^1^H NMR (400 MHz, DMSO-*d*_6_) δ 9.02 (s, 1H), 7.74 (d, *J* = 7.9 Hz, 2H), 7.48 (d, *J* = 8.5 Hz, 2H), 7.37 (t, *J* = 7.6 Hz, 2H), 7.31–7.24 (m, 4H), 7.19–7.15 (m, 2H), 6.52 (d, *J* = 8.6 Hz, 2H), 5.45 (s, 2H). ^13^C{^1^H} NMR (101 MHz, DMSO-*d*_6_) δ 148.5, 139.2, 135.2, 133.8, 130.7, 130.0, 128.8, 128.6, 128.0, 127.9, 127.8, 125.2, 119.6, 113.8, 100.3, 50.2. IR (υ/cm^−1^): 2962 (M), 2927 (W), 2223 (M), 1604 (S), 1590 (S), 1519 (S), 1456 (S), 1434 (S), 1426 (S), 1358 (S), 1323 (S), 1258 (S), 1173 (S), 1006 (S), 822 (VS), 773 (VS), 734 (VS), 716 (VS), 696 (VS). HRMS (ESI): calcd for C_22_H_18_N_5_ [M+H]^+^: 352.1557; found: 352.1556.

*1-tert-butyl-5-(p-tolylamino)-4-phenyl-1H-1,2,3-triazole* (**2e**)

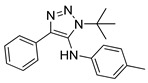



From 1-*tert*-butyl-4-phenyl-1*H*-1,2,3-triazol-5-amine and 1-bromo-4-methylbenzene, following general procedure, **2e** (100 mg, 65% yield) was obtained as a white solid, m.p. 241–242 °C. ^1^H NMR (400 MHz, Chloroform-*d*) δ 7.80–7.74 (m, 2H), 7.28–7.20 (m, 3H), 6.97 (d, *J* = 6.5 Hz, 2H), 6.46 (d, *J* = 6.1 Hz, 2H), 5.25 (s, 1H), 2.22 (s, 3H), 1.68 (s, 9H). ^13^C{^1^H} NMR (101 MHz, Chloroform-*d*) δ 142.6, 142.3, 131.8, 130.6, 130.3, 129.3, 128.6, 127.8, 126.1, 114.2, 61.3, 29.8, 20.6. IR (υ/cm^−1^): 3233 (M), 2975 (M), 1612 (S), 1593 (S), 1568 (S), 1516 (VS), 1449 (S), 1410 (S), 1371 (S), 1309 (VS), 1235 (S), 1195 (S), 991 (VS), 805 (VS), 762 (VS), 693 (VS). HRMS (ESI): calcd for C_19_H_23_N_4_ [M+H]^+^: 307.1917; found: 307.1921.

*1-benzyl-5-((4-fluoro-2-methylphenyl)amino)-4-phenyl-1H-1,2,3-triazole* (**2f**)

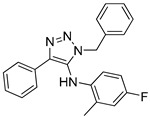



From 1-benzyl-4-phenyl-1*H*-1,2,3-triazol-5-amine and 1-bromo-4-fluoro- 2-methylbenzene, following general procedure, **2f** (177 mg, >99% yield) was obtained as a white solid, m.p. 216–217 °C. ^1^H NMR (400 MHz, Chloroform-*d*) δ 7.74 (dd, *J* = 8.2, 1.2 Hz, 2H), 7.33 (t, *J* = 7.3 Hz, 2H), 7.31–7.27 (m, 4H), 7.16–7.12 (m, 2H), 6.90 (dd, *J* = 9.1, 2.7 Hz, 1H), 6.70–6.60 (m, 1H), 6.15 (dd, *J* = 8.7, 4.8 Hz, 1H), 5.35 (s, 2H), 4.74 (s, 1H), 2.12 (s, 3H). ^13^C{^1^H} NMR (101 MHz, Chloroform-*d*) δ 157.4 (d, *J* = 239.0 Hz), 140.7, 137.5 (d, *J* = 2.0 Hz), 134.6, 132.1, 130.4, 129.0, 128.9, 128.6, 128.2, 127.8, 125.9, 125.4 (d, *J* = 7.6 Hz), 117.8 (d, *J* = 22.8 Hz), 114.1 (d, *J* = 8.2 Hz), 113.6 (d, *J* = 22.3 Hz), 51.8, 17.6. ^19^F NMR (376 MHz, Chloroform-*d*) δ -123.92. IR (υ/cm^−1^): 3241 (W), 1610 (S), 1588 (S), 1516 (S), 1498 (S), 1446 (S), 1411 (S), 1362 (S), 1268 (S), 1239 (S), 1199 (S), 1007 (S), 953 (S), 856 (VS), 800 (VS), 771 (VS), 737 (VS), 714 (VS), 697 (VS). HRMS (ESI): calcd for C_22_H_20_FN_4_ [M+H]^+^: 359.1667; found: 359.1670.

*1-tert-butyl-5-(phenylamino)-4-phenyl-1H-1,2,3-triazole* (**2g**)

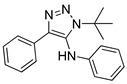



From 1-*tert*-butyl-4-phenyl-1*H*-1,2,3-triazol-5-amine and bromobenzene, following general procedure, **2g** (136 mg, 93% yield) was obtained as a white solid, m.p. 228–229 °C. ^1^H NMR (400 MHz, Chloroform-*d*) δ 7.76 (d, *J* = 7.3 Hz, 2H), 7.25–7.19 (m, 3H), 7.16 (t, *J* = 7.1 Hz, 2H), 6.81 (t, *J* = 7.3 Hz, 1H), 6.55 (d, *J* = 7.6 Hz, 2H), 5.58 (s, 1H), 1.68 (s, 9H). ^13^C{^1^H} NMR (101 MHz, Chloroform-*d*) δ 144.6, 142.5, 131.6, 130.2, 129.7, 128.6, 127.9, 126.2, 120.1, 114.1, 61.5, 29.8. IR (υ/cm^−1^): 3346 (W), 3056 (W), 2980 (W), 2931 (W), 1604 (S), 1566 (S), 1498 (S), 1423 (S), 1370 (S), 1309 (S), 1233 (S), 1183 (S), 990 (VS), 768 (VS), 746 (VS), 717 (VS), 690 (VS). HRMS (ESI): calcd for C_18_H_21_N_4_ [M+H]^+^: 293.1761; found: 293.1766.

*1-benzyl-5-((pyridine-2-yl)amino)-4-phenyl-1H-1,2,3-triazole* (**2h**)

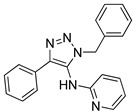



From 1-benzyl-4-phenyl-1*H*-1,2,3-triazol-5-amine and 2-bromopyridine, following general procedure, (**2h**) (127 mg, 77% yield) was obtained as a white solid, m.p. 173–174 °C. ^1^H NMR (400 MHz, DMSO-*d*_6_) δ 8.93 (s, 1H), 7.99–7.96 (m, 1H), 7.77 (d, *J* = 7.0 Hz, 2H), 7.57–7.52 (m, 1H), 7.37 (t, *J* = 7.5 Hz, 2H), 7.32–7.25 (m, 4H), 7.19 (dd, *J* = 7.6, 1.8 Hz, 2H), 6.76–6.72 (m, 1H), 6.62 (d, *J* = 8.5 Hz, 1H), 5.40 (s, 2H). ^13^C{^1^H} NMR (101 MHz, Chloroform-*d*) δ 156.1, 148.0, 141.2, 138.6, 134.6, 130.4, 130.2, 128.8, 128.7, 128.4, 128.2, 128.1, 125.9, 115.9, 107.1, 51.6. IR (υ/cm^−1^): 3140 (W), 3082 (W), 3062 (W), 2914 (M), 2856 (M), 1588 (S), 1522 (S), 1500 (S), 1436 (S), 1361 (S), 1319 (S), 1233 (S), 1213 (S), 1153 (VS), 1101 (S), 1074 (S), 996 (VS), 783 (VS), 772 (VS), 738 (VS). HRMS (ESI): calcd for C_20_H_18_N_5_ [M+H]^+^: 328.1557; found: 328.1561.

*1-benzyl-5-((4-tert-butylphenyl))amino)-4-phenyl-1H-1,2,3-triazole* (**2i**)

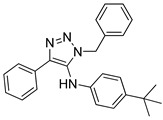



From 1-benzyl-4-phenyl-1*H*-1,2,3-triazol-5-amine and 1-bromo-4-*tert*-butylbenzene, following general procedure, **2i** (172 mg, 90% yield) was obtained as a white solid, m.p. 169–171 °C. ^1^H NMR (400 MHz, Chloroform-*d*) δ 7.81 (d, *J* = 7.0 Hz, 2H), 7.31–7.24 (m, 6H), 7.19–7.14 (m, 4H), 6.46 (d, *J* = 8.7 Hz, 2H), 5.33 (s, 2H), 5.07 (s, 1H), 1.27 (s, 9H). ^13^C{^1^H} NMR (101 MHz, Chloroform-*d*) δ 143.6, 141.0, 141.0, 134.8, 132.1, 130.4, 128.9, 128.8, 128.4, 128.0, 127.9, 126.6, 126.0, 114.1, 51.4, 34.2, 31.6. IR (υ/cm^−1^): 3253 (M), 3054 (M), 3034 (M), 2956 (M), 2900 (M), 2857 (M), 1607 (S), 1587 (S), 1568 (S), 1515 (VS), 1400 (S), 1360 (S), 1252 (S), 1190 (S), 922 (S), 814 (S), 770 (VS), 737 (VS), 719 (VS), 695 (VS). HRMS (ESI): calcd for C_25_H_27_N_4_ [M+H]^+^: 383.2230; found: 383.2241.

*1-benzyl-5-(mesitylamino)-4-phenyl-1H-1,2,3-triazole* (**2j**)

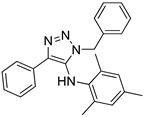



From 1-benzyl-4-phenyl-1*H*-1,2,3-triazol-5-amine and 2-bromo- 1,3,5-trimethylbenzene, following general procedure, **2j** (146 mg, 79% yield) was obtained as a white solid, m.p. 132–133 °C. ^1^H NMR (400 MHz, Chloroform-*d*) δ 7.71 (d, *J* = 7.3 Hz, 2H), 7.31–7.20 (m, 6H), 6.89 (dd, *J* = 7.2, 2.2 Hz, 2H), 6.72 (s, 2H), 5.15 (s, 2H), 4.93 (s, 1H), 2.23 (s, 3H), 1.75 (s, 6H). ^13^C{^1^H} NMR (101 MHz, Chloroform-*d*) δ 135.8, 135.1, 134.9, 134.9, 133.6, 131.2, 130.1, 129.8, 128.8, 128.4, 128.2, 127.2, 127.1, 126.2, 51.6, 20.7, 18.2. IR (υ/cm^−1^): 3339 (M), 3060 (W), 3032 (M), 2913 (M), 2853 (W), 1606 (S), 1586 (S), 1571 (S), 1485 (S), 1445 (S), 1421 (S), 1361 (S), 1317 (S), 1250 (S), 1073 (S), 1029 (S), 994 (S), 840 (S), 769 (VS), 724 (VS), 694 (VS). HRMS (ESI): calcd for C_24_H_25_N_4_ [M+H]^+^: 369.2074; found: 369.2074.

*1-tert-butyl-5-((pyridine-3-yl)amino)-4-phenyl-1H-1,2,3-triazole* (**2k**)

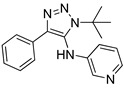



From 1-*tert*-butyl-4-phenyl-1*H*-1,2,3-triazol-5-amine and 3-chloropyridine, following general procedure, **2k** (110 mg, 75% yield) was obtained as a white solid, m.p. 233–234 °C. ^1^H NMR (400 MHz, DMSO-*d*_6_) δ 8.27 (s, 1H), 7.94 (s, 1H), 7.90 (d, *J* = 4.7 Hz, 1H), 7.73 (d, *J* = 7.9 Hz, 2H), 7.32 (t, *J* = 7.6 Hz, 2H), 7.24 (t, *J* = 7.6 Hz, 1H), 7.08 (dd, *J* = 8.3, 4.6 Hz, 1H), 6.73 (d, *J* = 8.0 Hz, 1H), 1.65 (s, 9H). ^13^C{^1^H} NMR (101 MHz, DMSO-*d*_6_) δ 141.6, 140.9, 139.9, 136.0, 131.1, 130.3, 128.6, 127.8, 125.4, 124.0, 119.5, 60.7, 29.1. IR (υ/cm^−1^): 3252 (W), 3002 (W), 2974 (W), 1589 (S), 1580 (S), 1508 (S), 1477 (S), 1449 (S), 1370 (S), 1299 (S), 1239 (S), 990 (VS), 800 (VS), 772 (VS), 709 (VS). HRMS (ESI) calcd for C_17_H_20_N_5_ [M+H]^+^: 294.1719; found: 294.1718.

*1-phenethyl-5-((pyridine-3-yl)amino)-4-phenyl-1H-1,2,3-triazole* (**2l**)

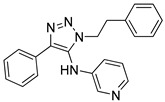



From 1-phenethyl-4-phenyl-1H-1,2,3-triazol-5-amine and 3-chloropyridine, following general procedure, **2l** (142 mg, 83% yield) was obtained as a white solid, m.p. 199–200 °C. ^1^H NMR (400 MHz, DMSO-*d*_6_) δ 8.46 (s, 1H), 8.01–7.92 (m, 2H), 7.72 (d, *J* = 7.3 Hz, 2H), 7.35 (t, *J* = 7.4 Hz, 2H), 7.29–7.16 (m, 4H), 7.12 (d, *J* = 7.2 Hz, 2H), 7.07 (dd, *J* = 8.0, 4.6 Hz, 1H), 6.65 (dd, *J* = 8.5, 1.3 Hz, 1H), 4.44 (t, *J* = 7.3 Hz, 2H), 3.13 (t, *J* = 7.6 Hz, 2H). ^13^C{^1^H} NMR (101 MHz, DMSO-*d*_6_) δ 140.6, 140.3, 138.3, 137.5, 136.4, 131.5, 130.3, 128.7, 128.6, 128.5, 127.7, 126.6, 125.2, 124.0, 119.7, 47.7, 35.0. IR (υ/cm^−1^): 3203 (W), 3162 (W), 3083 (W), 3025 (W), 2969 (W), 1582 (S), 1569 (S), 1480 (S), 1455 (S), 1402 (S), 1361 (S), 1312 (S), 1278 (S), 1232 (S), 990 (S), 799 VS, 763 (VS), 743 (VS), 701 (VS). HRMS (ESI): calcd for C_21_H_20_N_5_ [M+H]^+^: 342.1719; found: 342.1717.

*1-benzyl-5-((3,5-dimethylphenyl)amino)-4-phenyl-1H-1,2,3-triazole* (**2m**)

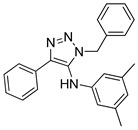



From 1-benzyl-4-phenyl-1*H*-1,2,3-triazol-5-amine and 1-bromo- 3,5-dimethylbenzene, following general procedure, **2m** (149 mg, 84% yield) was obtained as a white solid, m.p. 154–155 °C. ^1^H NMR (400 MHz, Chloroform-*d*) δ 7.82 (d, *J* = 7.0 Hz, 2H), 7.35–7.26 (m, 6H), 7.23–7.19 (m, 2H), 6.54 (s, 1H), 6.15 (s, 2H), 5.34 (s, 2H), 5.06 (s, 1H), 2.18 (s, 6H). ^13^C{^1^H} NMR (101 MHz, Chloroform-*d*) δ 143.7, 141.1, 139.7, 134.8, 131.9, 130.4, 128.9, 128.8, 128.4, 128.1, 128.0, 126.0, 122.7, 112.2, 51.4, 21.5. IR (υ/cm^−1^): 3266 (W), 2919 (W), 1601 (S), 1585 (S), 1495 (S), 1444 (S), 1353 (S), 1324 (S), 1233 (S), 1170 (S), 1004 (VS), 993 (VS), 837 (VS), 774 (VS), 739 (VS), 727 (VS), 691 (VS). HRMS (ESI): calcd for C_23_H_23_N_4_ [M+H]^+^: 355.1917; found: 355.1920.

*1-benzyl-5-((pyrimidine-4-yl)amino)-4-phenyl-1H-1,2,3-triazole* (**2n**)

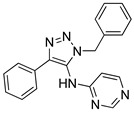



From 1-benzyl-4-phenyl-1*H*-1,2,3-triazol-5-amine and 4-chloropyrimidine, following general procedure, **2n** (161 mg, 98% yield) was obtained as a white solid, m.p. 156–157 °C. ^1^H NMR (400 MHz, DMSO-*d*_6_) δ 9.40 (s, 1H), 8.10 (s, 1H), 7.93 (d, *J* = 5.6 Hz, 2H), 7.75 (d, *J* = 7.7 Hz, 2H), 7.38 (t, *J* = 7.6 Hz, 2H), 7.27 (q, *J* = 7.7, 6.7 Hz, 4H), 7.18 (d, *J* = 7.7 Hz, 2H), 5.44 (s, 2H). ^13^C{^1^H} NMR (101 MHz, DMSO-*d*_6_) δ 152.5, 141.8, 139.4, 135.3, 135.0, 133.0, 130.5, 130.4, 128.7, 128.5, 127.8, 127.8, 127.8, 125.3, 50.5. IR (υ/cm^−1^): 3189 (W), 3067 (W), 2953 (W), 1593 (S), 1497 (S), 1472 (S), 1446 (S), 1360 (S), 1318 (S), 1278 (S), 1231 (S), 1150 (S), 996 (S), 825 (VS), 767 (VS), 734 (VS), 694 (VS). HRMS (ESI) calcd for C_19_H_17_N_6_ [M+H]^+^: 329.1515; found: 329.1514.

*1-benzyl-5-((pyridine-3-yl)amino)-4-phenyl-1H-1,2,3-triazole* (**2o**)

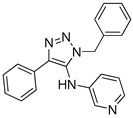



From 1-benzyl-4-phenyl-1*H*-1,2,3-triazol-5-amine and 3-chloropyridine (159 mg, 97% yield) or 3-bromopyridine (163 mg, >99% yield), following general procedure, **2o** was obtained as a white solid, m.p. 169–170 °C. ^1^H NMR (400 MHz, Chloroform-*d*) δ 8.02–7.97 (m, 2H), 7.73 (dd, *J* = 7.9, 1.6 Hz, 2H), 7.27–7.22 (m, 3H), 7.21–7.17 (m, 3H), 7.15–7.11 (m, 2H), 6.92 (dd, *J* = 8.3, 4.7 Hz, 1H), 6.52 (ddd, *J* = 8.3, 2.7, 1.2 Hz, 1H), 6.30 (s, 1H), 5.36 (s, 2H). ^13^C{^1^H} NMR (101 MHz, Chloroform-*d*) δ 141.3, 141.3, 140.3, 136.9, 134.3, 130.6, 129.9, 129.0, 128.8, 128.6, 128.4, 127.9, 125.9, 124.1, 120.3, 51.6. IR (υ/cm^−1^): 3221 (W), 3173 (M), 3090 (W), 3043 (M), 3027 (M), 2962 (M), 2904 (M), 2780 (M), 1608 (S), 1583 (S), 1570 (S), 1538 (S), 1480 (S), 1427 (S), 1409 (S), 1364 (S), 1321 (S), 1246 (S), 1234 (S), 1048 (S), 1006 (S), 994 (S). HRMS (ESI): calcd for C_20_H_18_N_5_ [M+H]^+^: 328.1557; found: 328.1561.

*N4,N6-bis(1-benzyl-4-phenyl-1H-1,2,3-triazol-5-yl)pyrimidine-4,6-diamine* (**2p**)

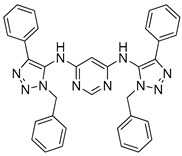



From 1-benzyl-4-phenyl-1*H*-1,2,3-triazol-5-amine and 4,6-dichloropyrimidine, following general procedure, **2p** (88 mg, 61% yield) was obtained as a white solid, m.p. 263–264 °C. ^1^H NMR (400 MHz, DMSO-*d*_6_) δ 9.28 (s, 2H), 7.98 (s, 1H), 7.73 (s, 4H), 7.40 (s, 4H), 7.37–7.29 (m, 3H), 7.28–7.10 (m, 10H), 5.38 (s, 4H). ^13^C{^1^H} NMR (101 MHz, DMSO-*d*_6_) δ 161.3, 158.2, 139.4, 135.2, 130.2, 130.1, 128.7, 128.5, 127.9, 127.8, 125.3, 50.5. IR (υ/cm^−1^): 3064 (M), 3032 (M), 2927 (M), 1601 (S), 1587 (S), 1496 (S), 1356 (S), 1288 (S), 1237 (S), 1188 (S), 1073 (S), 991 (S), 822 (VS), 769 (VS), 734 (VS), 720 (VS), 692 (VS). HRMS (ESI): calcd for C_34_H_29_N_10_ [M+H]^+^: 577.2571; found: 577.2574.

*1-phenethyl-5-(p-tolylamino)-4-phenyl- 1H-1,2,3-triazole* (**2q**)

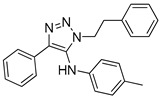



From 5-chloro-1-phenethyl-4-phenyl-1*H*-1,2,3-triazole and *p*-toluidine, following general procedure, **2q** (147 mg, 83% yield) was obtained as a white solid, m.p. 152–153 °C. ^1^H NMR (400 MHz, Chloroform-*d*) δ 7.74 (d, *J* = 7.9 Hz, 2H), 7.30–7.23 (m, 6H), 7.04–6.99 (m, 2H), 6.93 (d, *J* = 8.0 Hz, 2H), 6.29 (d, *J* = 7.5 Hz, 2H), 4.77 (s, 1H), 4.37 (t, *J* = 8.3 Hz, 2H), 3.14 (t, *J* = 8.4 Hz, 2H), 2.21 (s, 3H). ^13^C{^1^H} NMR (101 MHz, Chloroform-*d*) δ 140.8, 139.5, 137.6, 132.9, 130.2, 129.9, 129.1, 129.0, 128.7, 128.4, 127.3, 126.2, 125.9, 114.2, 49.2, 36.4, 20.6. IR (υ/cm^−1^): 3205 (M), 3176 (M), 3085 (M), 3027 (M), 2950 (M), 2931 (M), 1878 (M), 1610 (S), 1585 (S), 1572 (S), 1520 (S), 1498 (S), 1451 (S), 1364 (S), 1258 (S), 1011 (S), 807 (VS), 762 (VS), 748 (VS), 699 (VS). HRMS (ESI): calcd for C_23_H_23_N_4_ [M+H]^+^: 355.1917; found: 355.1920.

*1-benzyl-5-((2,4-dimethylphenyl)amino)-4-phenyl-1H-1,2,3-triazole* (**2r**)

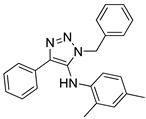



From 1-benzyl-5-chloro-4-phenyl-1*H*-1,2,3-triazole and 2,4-dimethylaniline, following general procedure, **2r** (169 mg, 95% yield) was obtained as a white solid, m.p. 194–195 °C. ^1^H NMR (400 MHz, Chloroform-*d*) δ 7.77 (d, *J* = 7.5 Hz, 2H), 7.33–7.24 (m, 6H), 7.16–7.11 (m, 2H), 6.99 (s, 1H), 6.78 (d, *J* = 8.2 Hz, 1H), 6.16 (d, *J* = 8.1 Hz, 1H), 5.31 (s, 2H), 4.84 (s, 1H), 2.25 (s, 3H), 2.11 (s, 3H). ^13^C{^1^H} NMR (101 MHz, Chloroform-*d*) δ 140.5, 139.0, 134.7, 132.5, 131.8, 130.2, 130.1, 129.0, 128.8, 128.5, 128.1, 127.9, 127.9, 126.0, 123.5, 113.0, 51.8, 20.6, 17.5. IR (υ/cm^−1^): 3260 (W), 2962 (W), 2924 (W), 2857 (W), 1608 (S), 1587 (S), 1571 (S), 1517 (S), 1446 (S), 1360 (S), 1237 (S), 1156 (S), 804 (VS), 766 (VS), 736 (VS), 694 (VS). HRMS (ESI): calcd for C_23_H_23_N_4_ [M+H]^+^: 355.1917; found: 355.1918.

*1-benzyl-5-((3-(trifluoromethyl)phenyl)amino)-4-phenyl-1H-1,2,3-triazole* (**2s**)

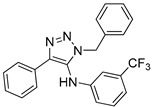



From 1-benzyl-5-chloro-4-phenyl-1*H*-1,2,3-triazole and 3-(trifluoromethyl)aniline, following general procedure, **2s** (159 mg, 81% yield) was obtained as a white solid, m.p. 115–117 °C. ^1^H NMR (400 MHz, Chloroform-*d*) δ 7.72 (dd, *J* = 6.4, 2.9 Hz, 2H), 7.23–7.11 (m, 9H), 7.05 (d, *J* = 7.7 Hz, 1H), 6.81 (s, 1H), 6.55 (d, *J* = 8.0 Hz, 1H), 6.41 (m, 1H), 5.32 (s, 2H). ^13^C{^1^H} NMR (101 MHz, Chloroform-*d*) δ 144.3, 141.3, 134.2, 131.9 (q, *J* = 32.3 Hz), 131.1, 130.2, 129.8, 128.9, 128.8, 128.5, 128.3, 128.0, 125.9, 124.0 (q, *J* = 272.8 Hz), 116.9, 110.9 (q, *J* = 3.6 Hz), 51.5. ^19^F NMR (376 MHz, Chloroform-*d*) δ -62.8. IR (υ/cm^−1^): 3195 (M), 3038 (M), 2927 (M), 1619 (S), 1586 (S), 1571 (S), 1495 (S), 1486 (S), 1444 (S), 1425 (S), 1336 (VS), 1231 (S), 1163 (VS), 1118 (VS), 1099 (S), 1067 (VS), 1006 (S), 996 (S), 916 (S), 871 (S), 791 (S), 769 (VS), 736 (VS), 692 (VS). HRMS (ESI): calcd for C_22_H_18_F_3_N_4_ [M+H]^+^: 395.1478; found: 395.1482.

*1-benzyl-5-((3,5-bis(trifluoromethyl)phenyl)amino)-4-phenyl-1H-1,2,3-triazole* (**2t**)

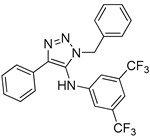



From 1-benzyl-5-chloro-4-phenyl-1*H*-1,2,3-triazole and 3,5-bis(trifluoromethyl) aniline, following general procedure, **2t** (136 mg, 59% yield) was obtained as a white solid, m.p. 110–111 °C. ^1^H NMR (400 MHz, Chloroform-*d*) δ 7.70 (dd, *J* = 7.7, 1.9 Hz, 2H), 7.35–7.26 (m, 4H), 7.24–7.22 (m, 3H), 7.16–7.11 (m, 2H), 6.75 (s, 2H), 5.52 (s, 1H), 5.42 (s, 2H). ^13^C{^1^H} NMR (101 MHz, Chloroform-*d*) δ 144.8, 141.9, 133.8, 133.0 (q, *J* = 33.5 Hz), 129.8, 129.5, 129.2, 129.0, 128.8, 128.7, 127.9, 126.0, 125.8, 123.1 (q, *J* = 272.6 Hz), 113.8 (p, *J* = 3.8 Hz), 113.7, 113.6, 52.0. ^19^F NMR (376 MHz, Chloroform-*d*) δ -63.19. IR (υ/cm^−1^): 3457 (W), 3204 (W), 3074 (W), 2930 (W), 1616 (S), 1590 (S), 1498 (S), 1471 (S), 1387 (S), 1276 (S), 1182 (S), 1130 (VS), 953 (VS), 873 (VS), 766 (VS), 700 (VS). HRMS (nESI): calcd for C_23_H_17_F_6_N_4_ [M+H]^+^: 463.1357; found: 463.1348.

*1-benzyl-4-(p-tolylamino)-5-methyl-1H-1,2,3-triazole* (**2u**)

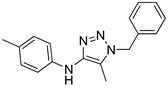



From 1-benzyl-4-bromo-5-methyl-1*H*-1,2,3-triazole and *p*-toluidine, following general procedure, **2u** (97 mg, 69% yield) was obtained as a white solid, m.p. 133–134 °C. ^1^H NMR (400 MHz, Chloroform-*d*) δ 7.38–7.32 (m, 3H), 7.19 (d, *J* = 6.7 Hz, 2H), 6.98 (d, *J* = 8.3 Hz, 2H), 6.63 (d, *J* = 8.1 Hz, 2H), 5.61 (s, 1H), 5.48 (s, 2H), 2.24 (s, 3H), 2.02 (s, 3H). ^13^C{^1^H} NMR (101 MHz, Chloroform-*d*) δ 144.7, 142.4, 134.6, 129.8, 129.2, 129.1, 128.5, 127.3, 125.1, 114.8, 52.9, 29.8, 20.6. IR (υ/cm^−1^): 3246 (M), 3109 (W), 3034 (M), 2924 (M), 2855 (M), 1884 (M), 1602 (S), 1511 (S), 1455 (S), 1435 (S), 1390 (S), 1345 (S), 1234 (S), 1121 (S), 815 (VS), 725 (VS), 696 (VS). HRMS (ESI): calcd for C_17_H_18_N_5_ [M+H]^+^: 279.1604; found: 279.1606.

## 4. Conclusions

In conclusion, we have developed an efficient and robust method for the preparation of a series of new 5-(het)arylamino-1,2,3-triazole derivatives via Buchwald–Hartwig cross-coupling reaction of 5-amino or 5-halo-1,2,3-triazoles with (het)aryl halides and amines respectively. As a result of the careful screening for optimal conditions, a catalytic system based on the palladium complex [(THP-Dipp)Pd(cinn)Cl] with expanded-ring NHC ligand has been revealed as the most active for the process. The reaction functions perfectly in 1,4-dioxane medium at 120 °C in the presence of an excess of *t*-BuONa to afford a variety of *5*-(het)arylamino-1,2,3-triazoles with good to excellent yields. The compounds obtained have major potential to be used in biomolecular chemistry and material science.

## Figures and Tables

**Table 1:**
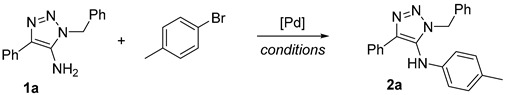
Screening of catalytic systems in the BHA reaction ^1^.

Entry	[Pd] (mol.%)	Base (Equiv.)	Yield, %
1	(THP-Dipp)Pd(cinn)Cl (1)	*t*-BuONa (1.2)	53
2	(THP-Dipp)Pd(cinn)Cl (2)	*t*-BuONa (1.2)	86
**3**	**(THP-Dipp)Pd(cinn)Cl (2)**	***t*-BuONa (3.0)**	**97**
4	(THP-Dipp)PdAllylCl (2)	*t*-BuONa (1.2)	73
5	(THP-Dipp)PdMetallylCl (2)	*t*-BuONa (1.2)	39
6	Pd(OAc)_2_ (2 mol %)/RuPhos (4)	*t*-BuONa (1.2)	30
7	Pd(OAc)_2_ (2 mol %)/SPhos (4)	*t*-BuONa (1.2)	5
8	Pd(OAc)_2_ (2 mol %)/DavePhos (4)	*t*-BuONa (1.2)	12
9	Pd(OAc)_2_ (2 mol %)/XPhos (4)	*t*-BuONa (1.2)	6
10	(THP-Dipp)Pd(cinn)Cl (2)	Cs_2_CO_3_ (1.2)	16

^1^ Reaction conditions:1-benzyl-4-phenyl-1*H*-1,2,3-triazol-5-amine **1a** (0.5 mmol); 1-bromo-4-methylbenzene (1 equiv.); [Pd], base; 1,4-dioxane (2.5 mL); 120 °C, 24 h.

## Data Availability

Data are contained within the article and Supplementary Materials.

## Supplementary Materials

The following are available online at https://www.mdpi.com/article/10.3390/molecules27061999/s1, copies of ^1^H and ^13^C NMR spectra for all novel compounds.

## Sample Availability

Samples of all of the compounds are available from the authors.
