# Supplementary material for: General Method of Synthesis of 5-(Het)arylamino-1,2,3-triazoles via Buchwald–Hartwig Reaction of 5-Amino- or 5-Halo-1,2,3-triazoles
Source: Molecules. 2022 Mar 20;27(6):1999. doi: 10.3390/molecules27061999 (PMC8949195; doi:10.3390/molecules27061999)
Supplement: Supplementary file 1 [file molecules-27-01999-s001.zip › molecules-1638595-supplementary.pdf]

Supporting information *for*

General method of synthesis of 5-(het)arylamino-1,2,3-triazoles *via* Buchwald–Hartwig reaction of 5-amino- or 5-halo-1,2,3-triazoles

Pavel S. Gribanov, Anna N. Philippova, Maxim A. Topchiy, Lidiya I. Minaeva, Andrey F. Asachenko, and Sergey N. Osipov

NMR spectra

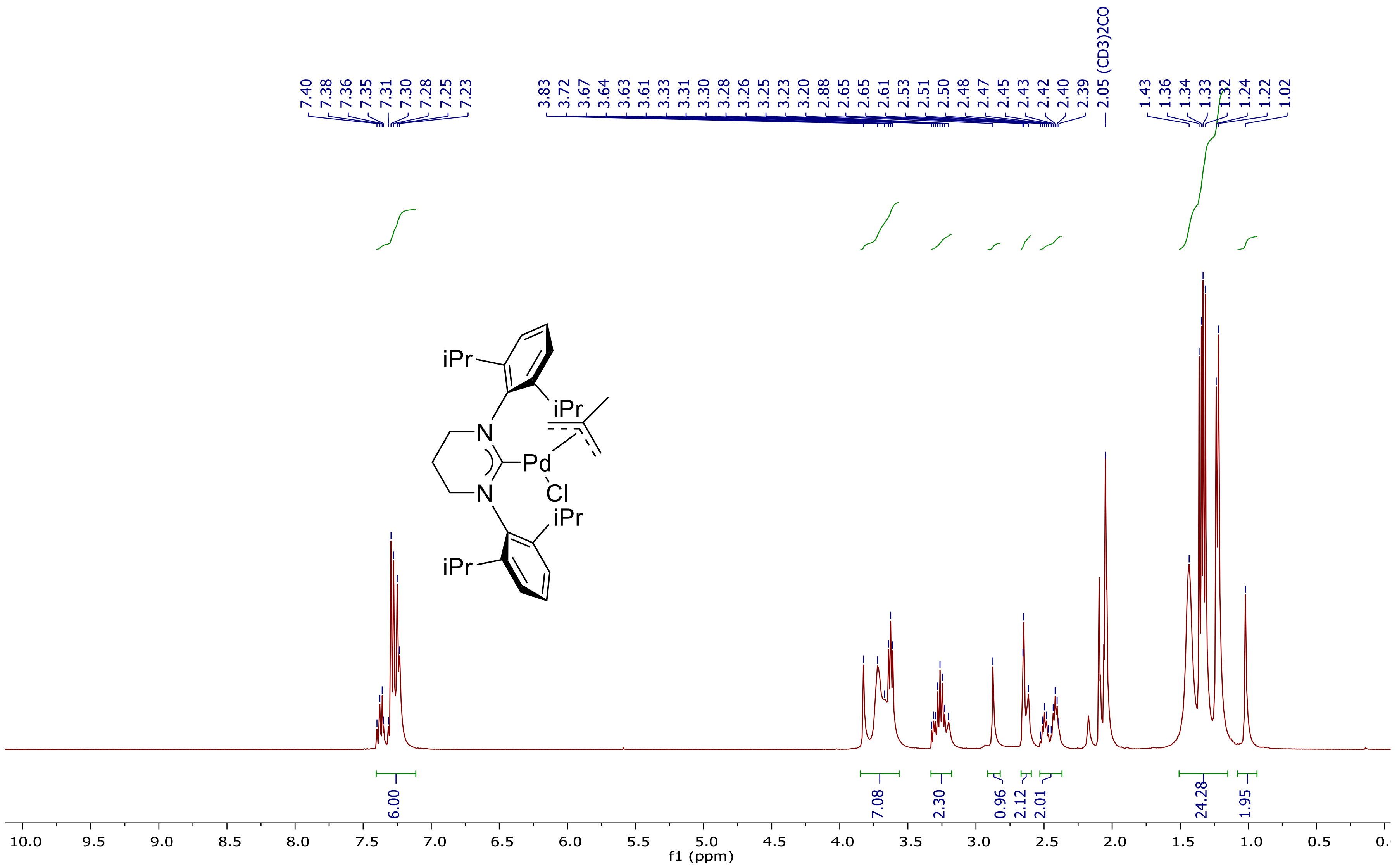

Figure S1. ^{1}**H NMR (400 MHz, Acetone-d6) of (THP-Dipp)Pd(methallyl)Cl**

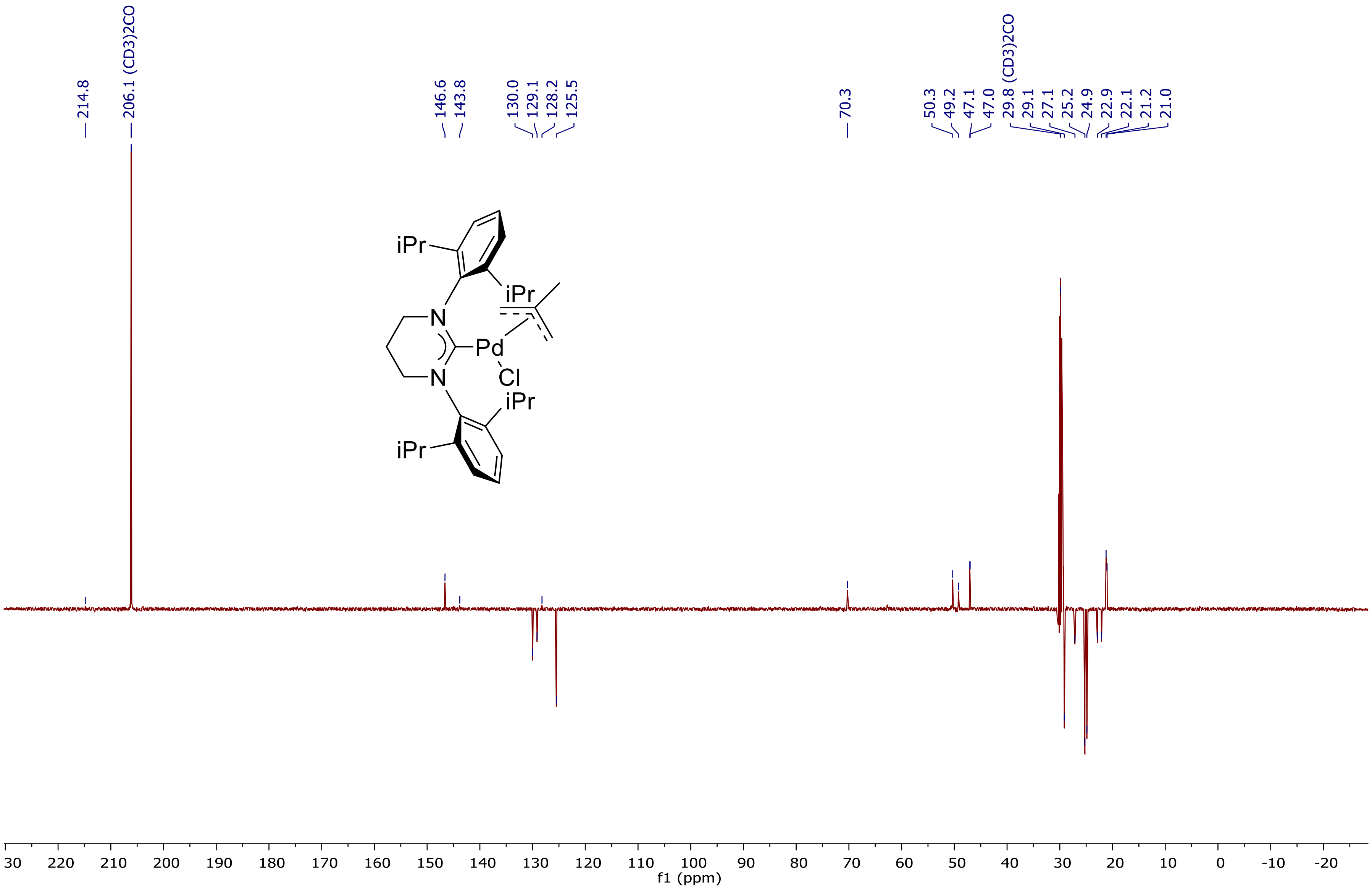

Figure S2. ^{13}**C DEPTQ-135 NMR (101 MHz, Acetone-d6) of (THP-Dipp)Pd(methallyl)Cl**

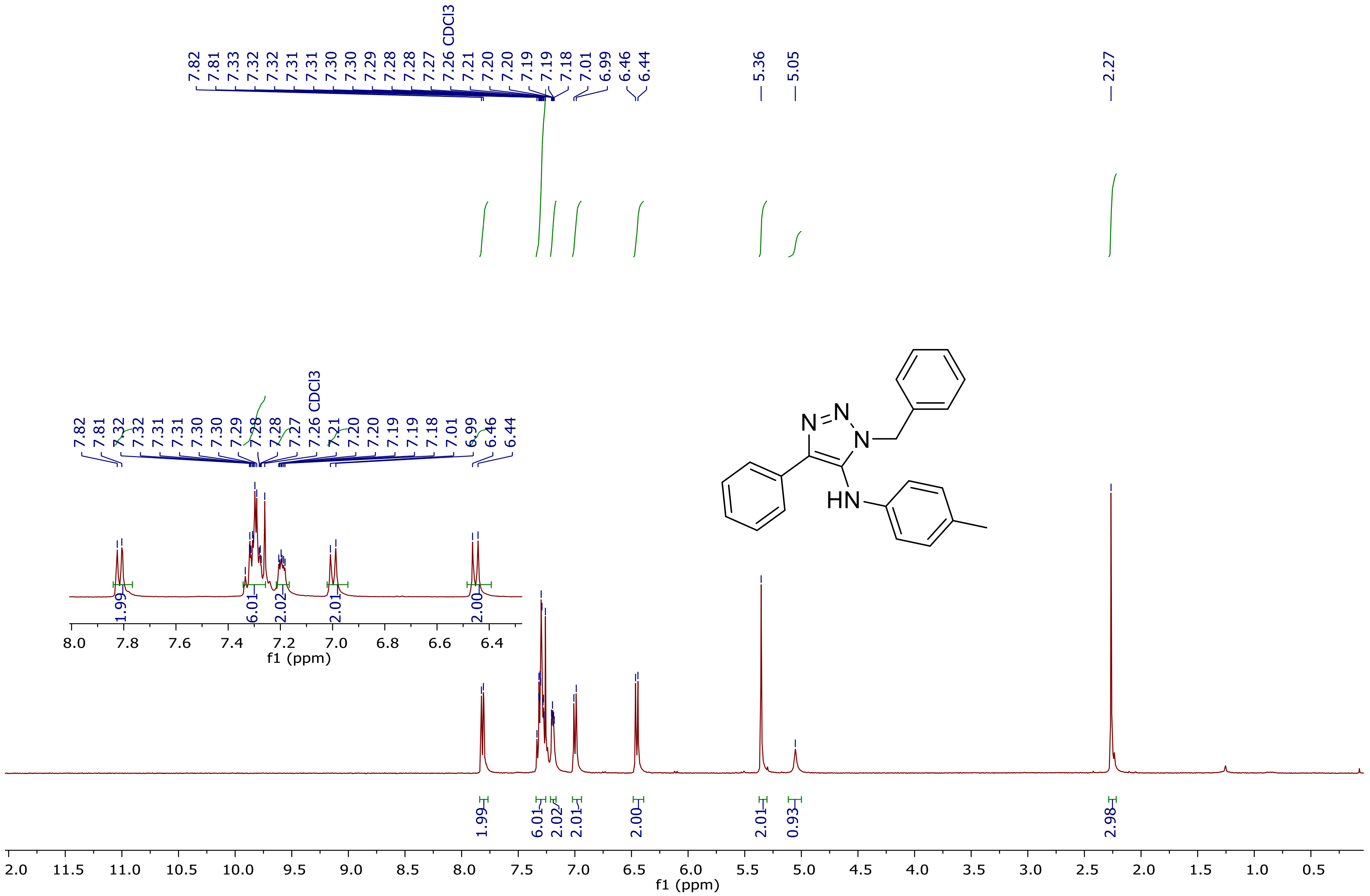

Figure S3. ^{1}H NMR (400 MHz, Chloroform-*d*) of 2a

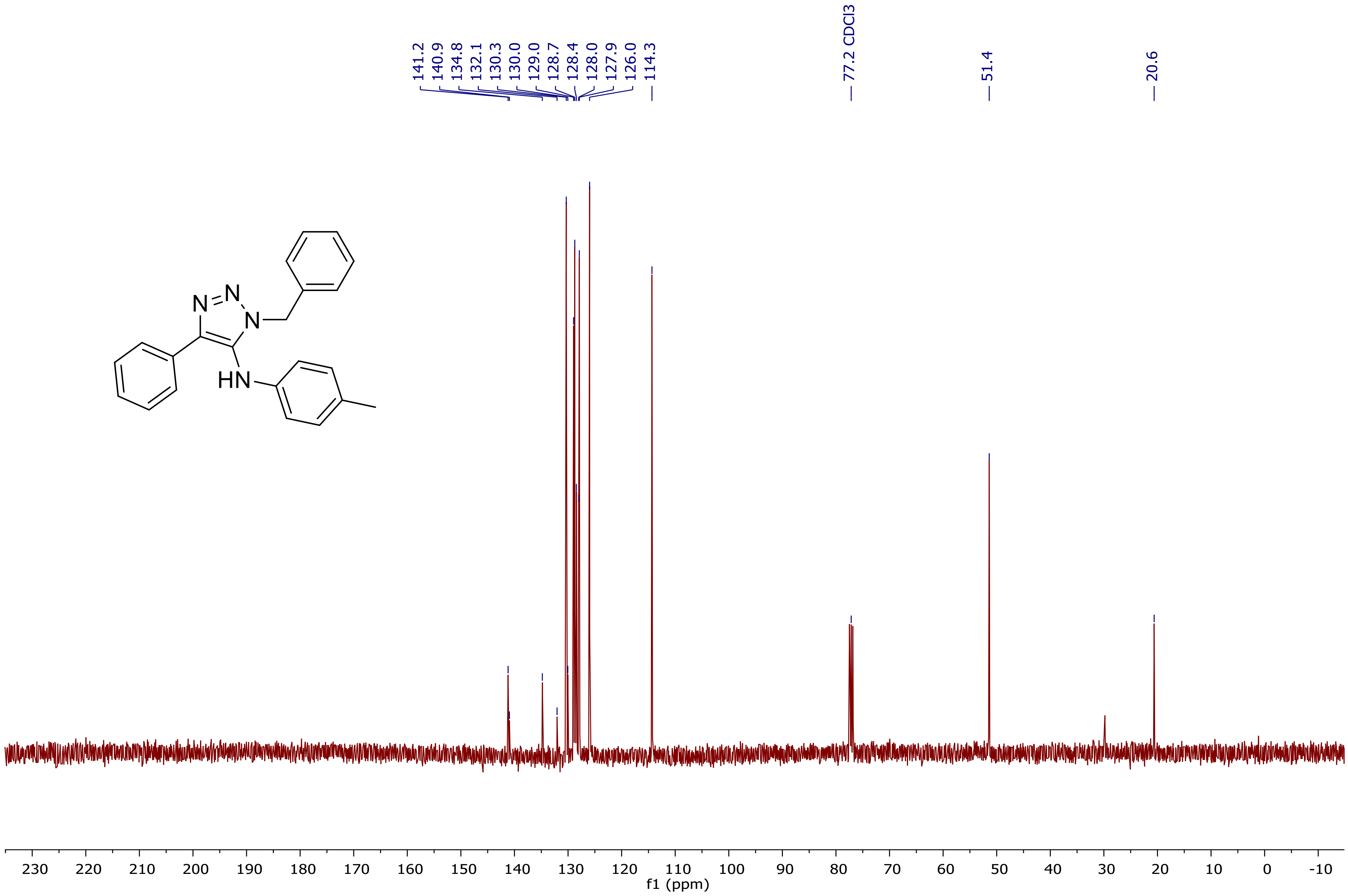

Figure S4. $^{13}C\{^1H\}$ **NMR (101 MHz, Chloroform-*d*) of 2a**

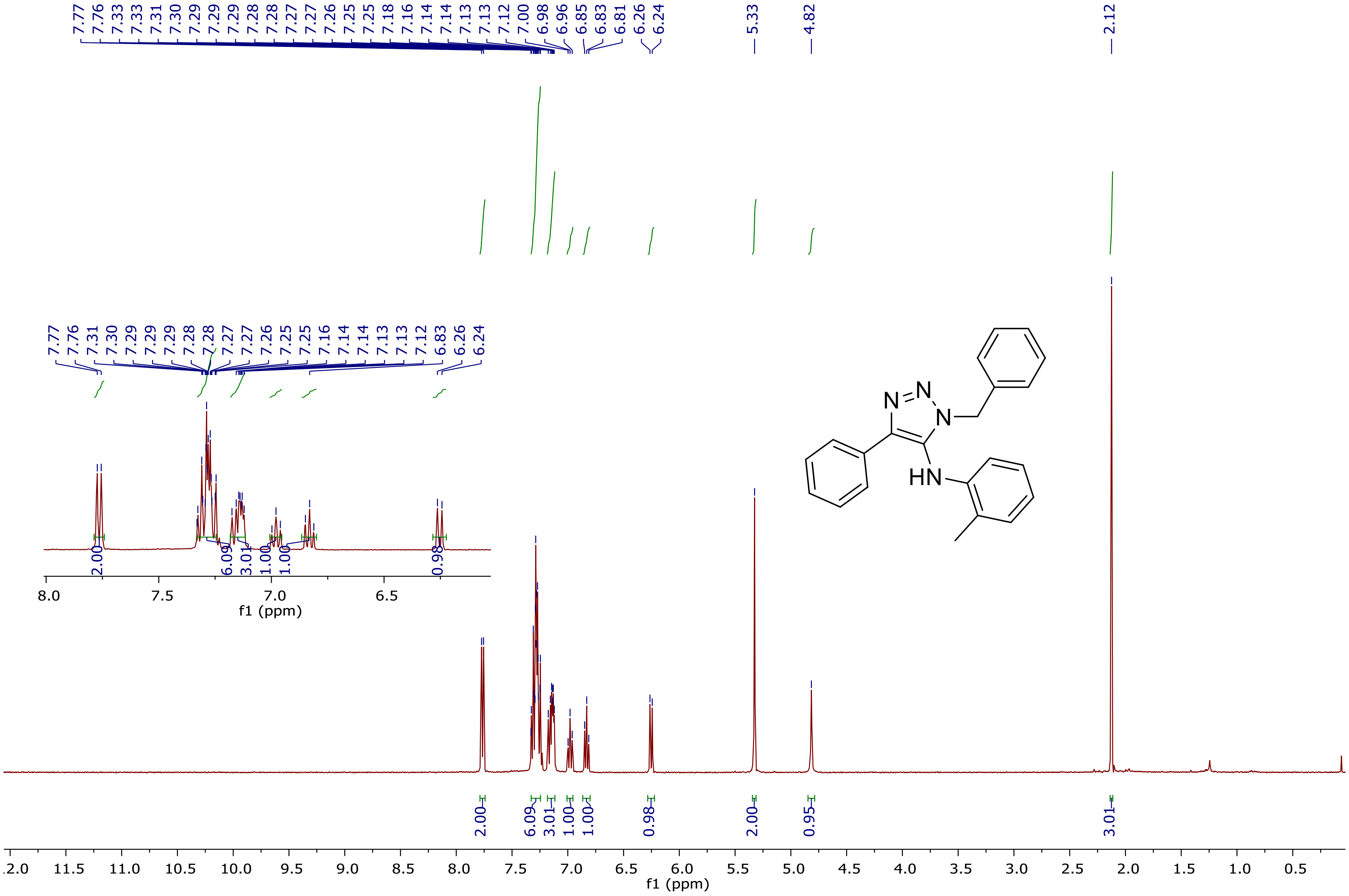

Figure S5. ^{1}H **NMR (400 MHz, Chloroform-*d*) of 2b**

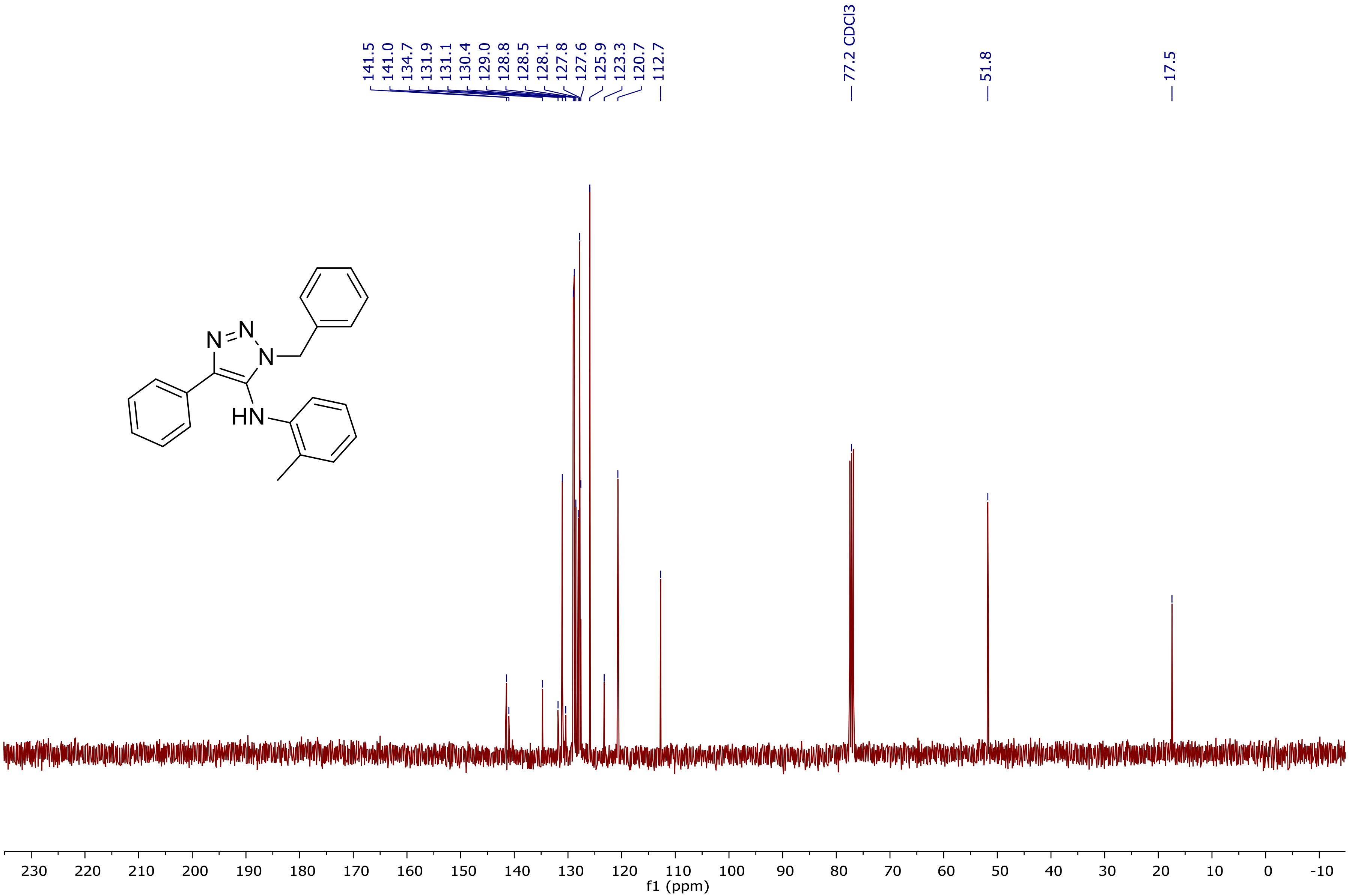

Figure S6. $^{13}C\{^{1}H\}$ **NMR (101 MHz, Chloroform-*d*) of 2b**

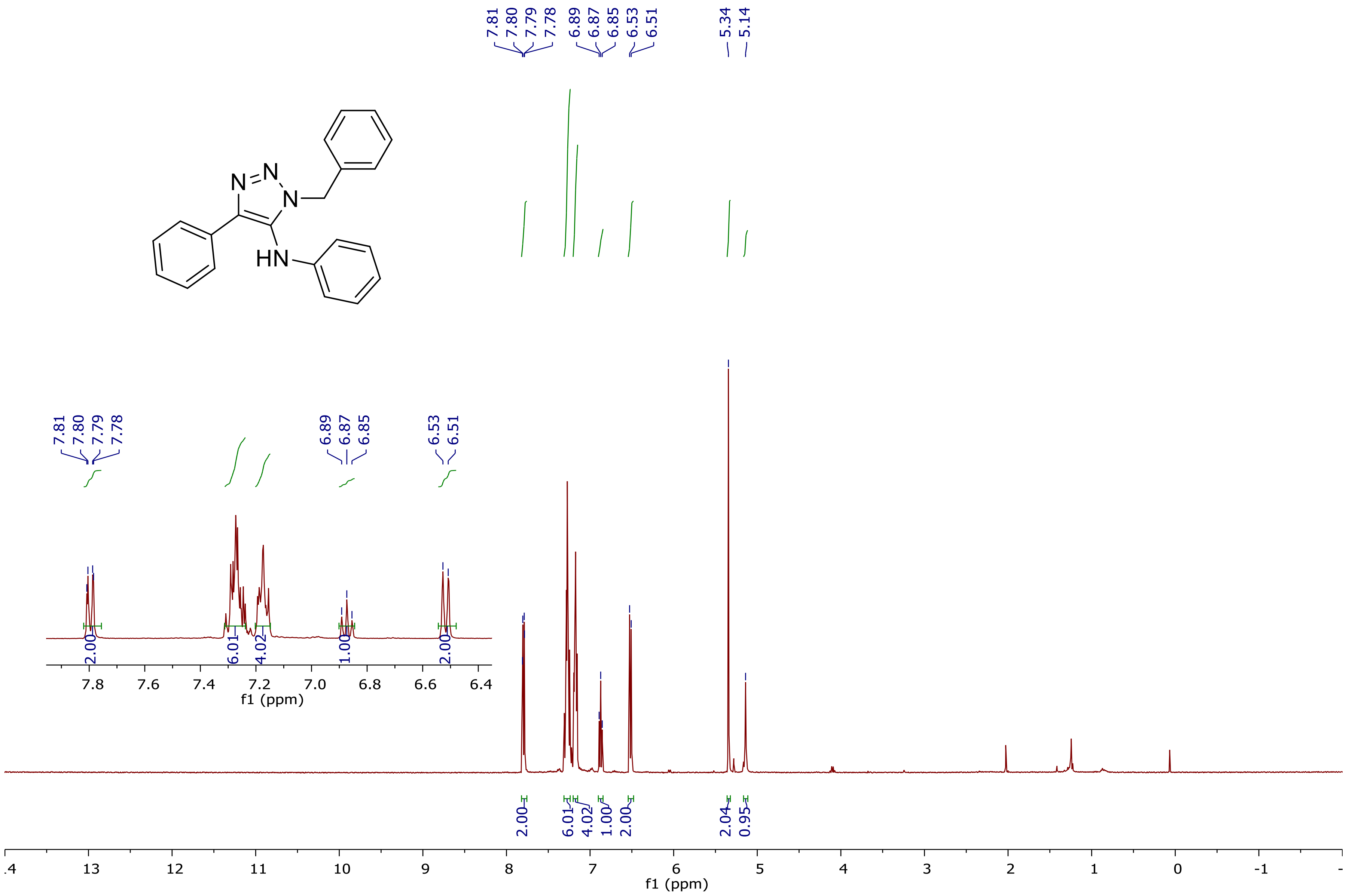

Figure S7. **^{1}H NMR (400 MHz, Chloroform-*d*) of 2c**

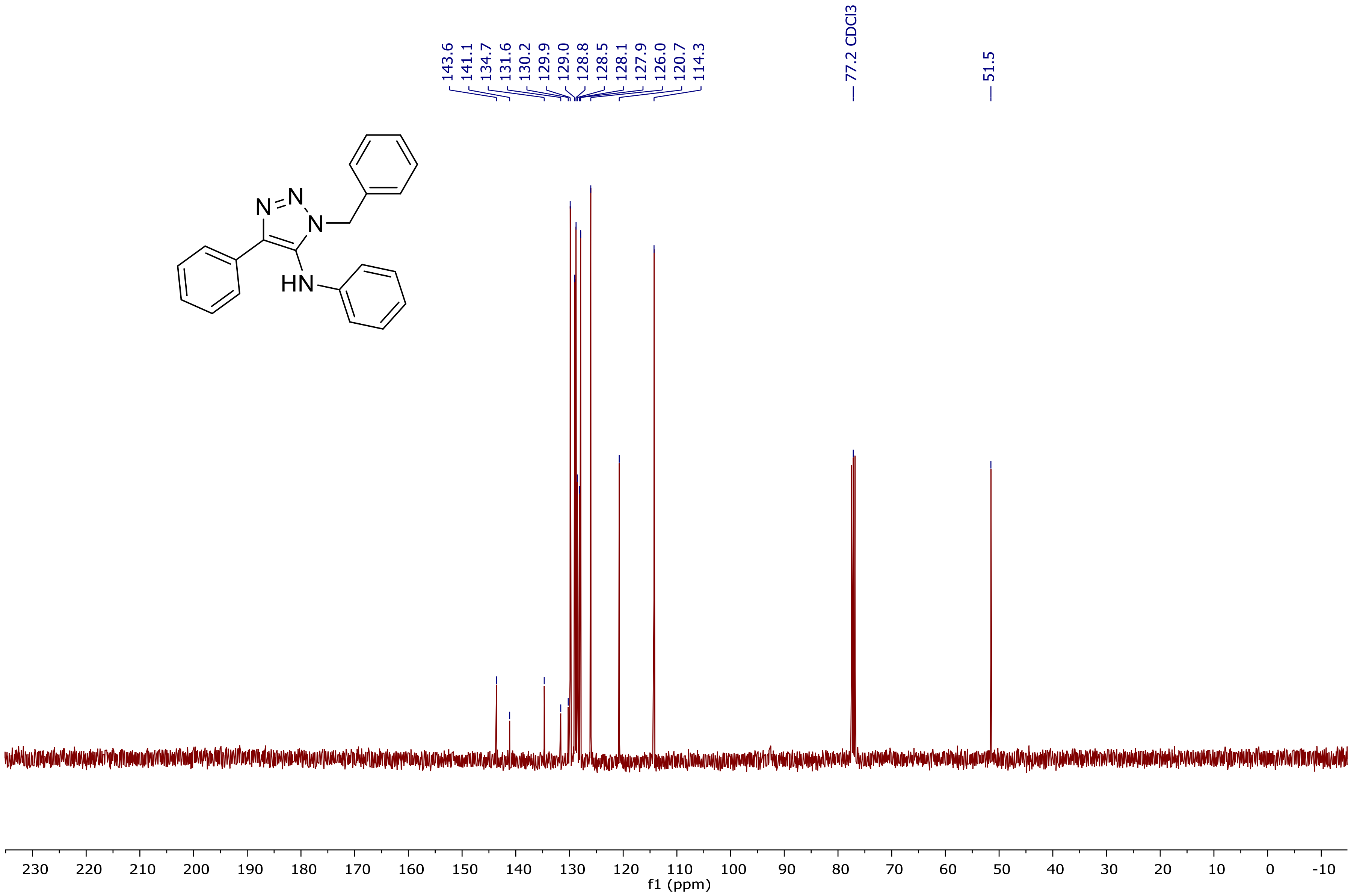

Figure S8. $^{13}C\{^{1}H\}$ **NMR (101 MHz, Chloroform-*d*) of 2c**

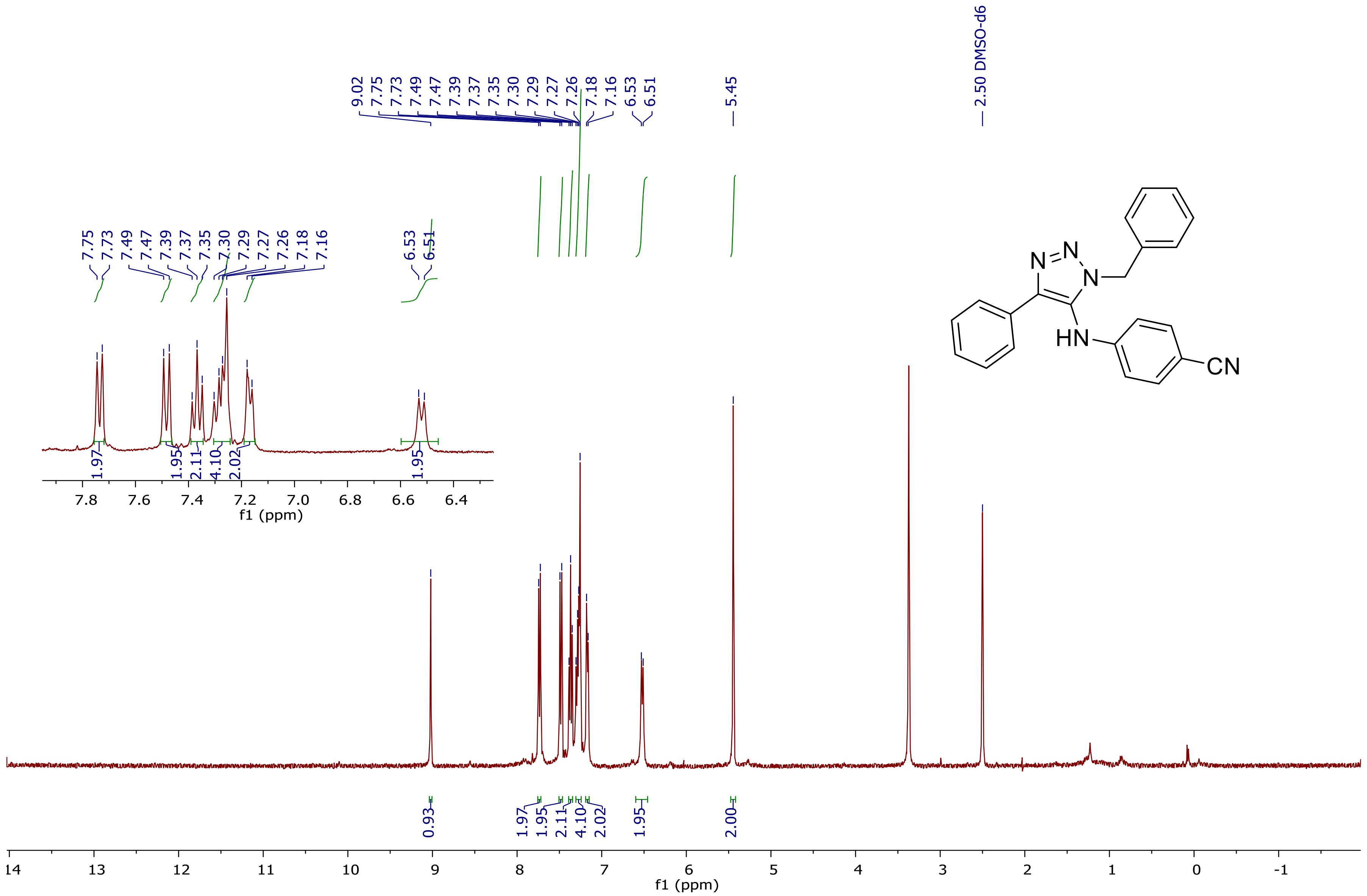

Figure S9. ^{1}H NMR (400 MHz, DMSO-d_6) of 2d

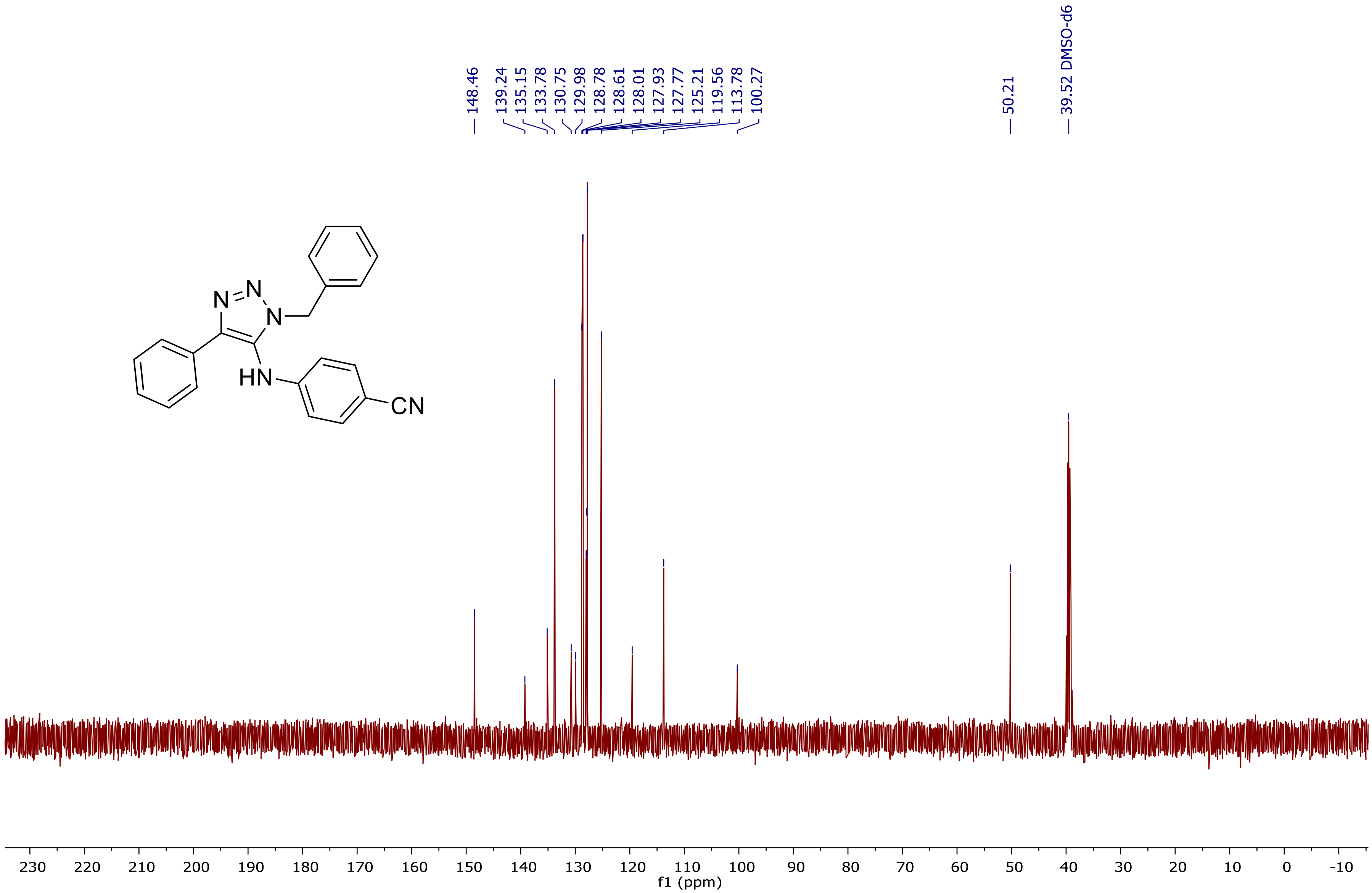

Figure S10. $^{13}C\{^{1}H\}$ **NMR (101 MHz, DMSO-*d*$_6$) of 2d**

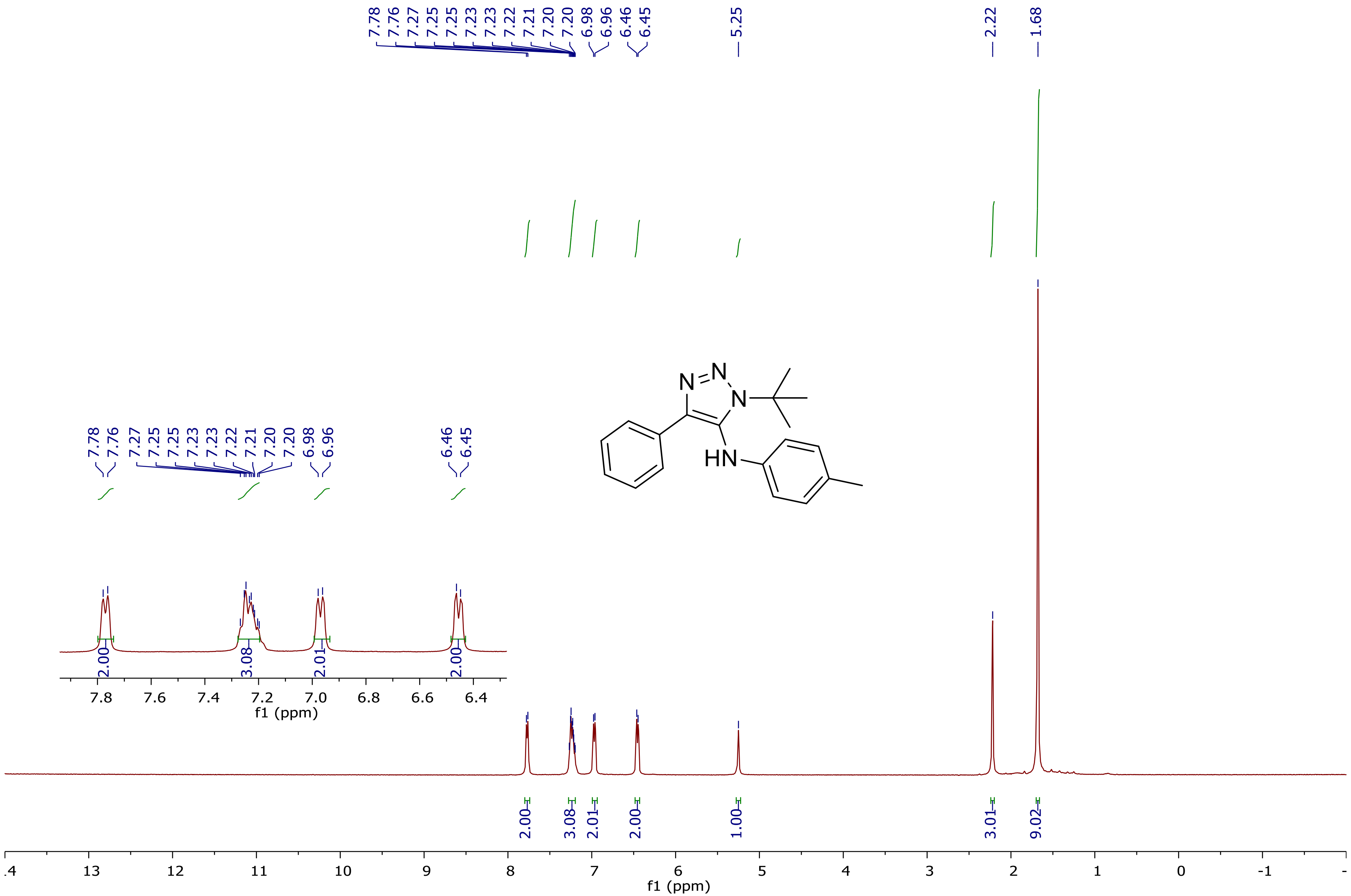

Figure S11. ^{1}H NMR (400 MHz, Chloroform-*d*) of 2e

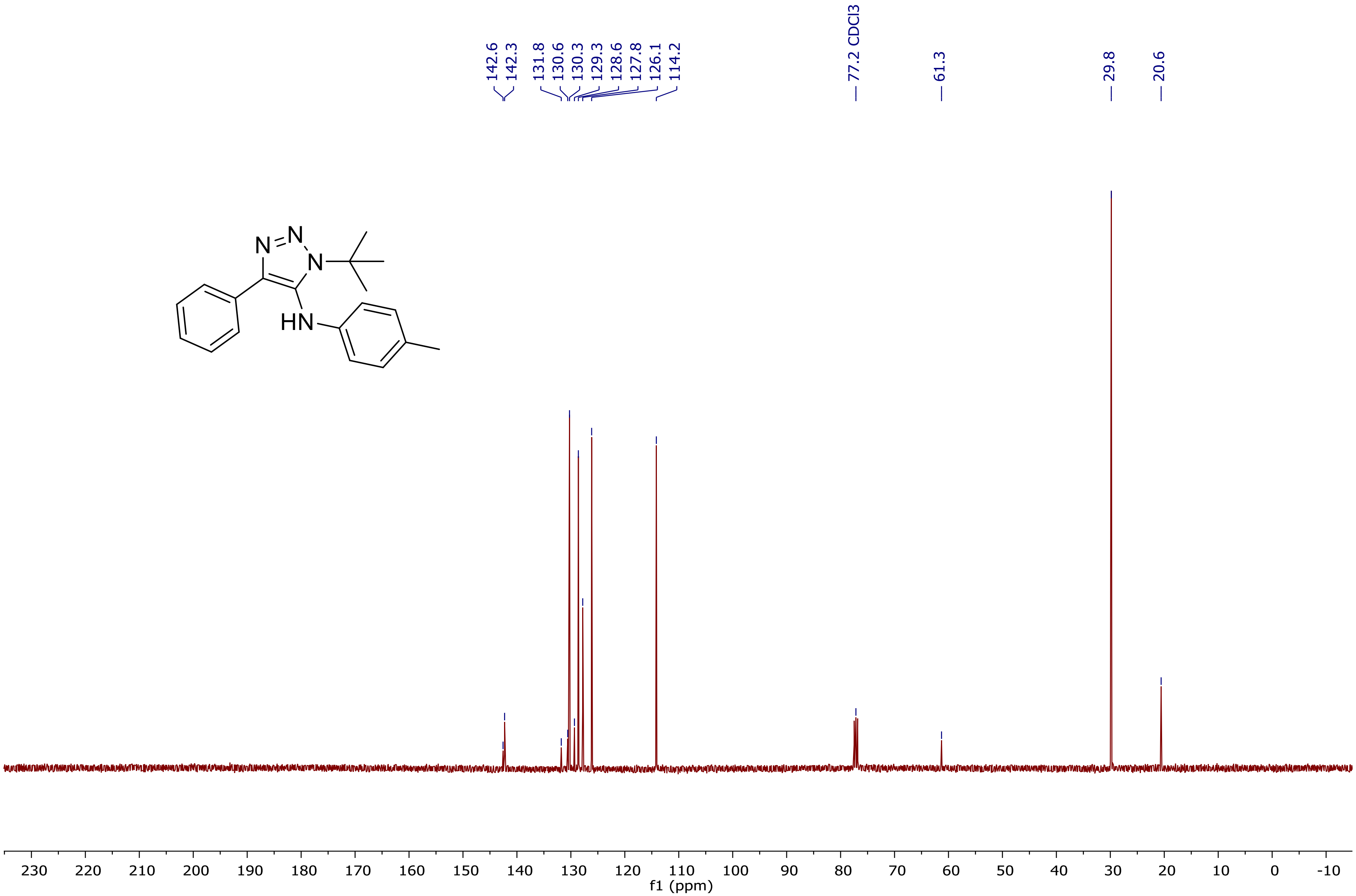

Figure S12. $^{13}C\{^{1}H\}$ **NMR (101 MHz, Chloroform-*d*) of 2e**

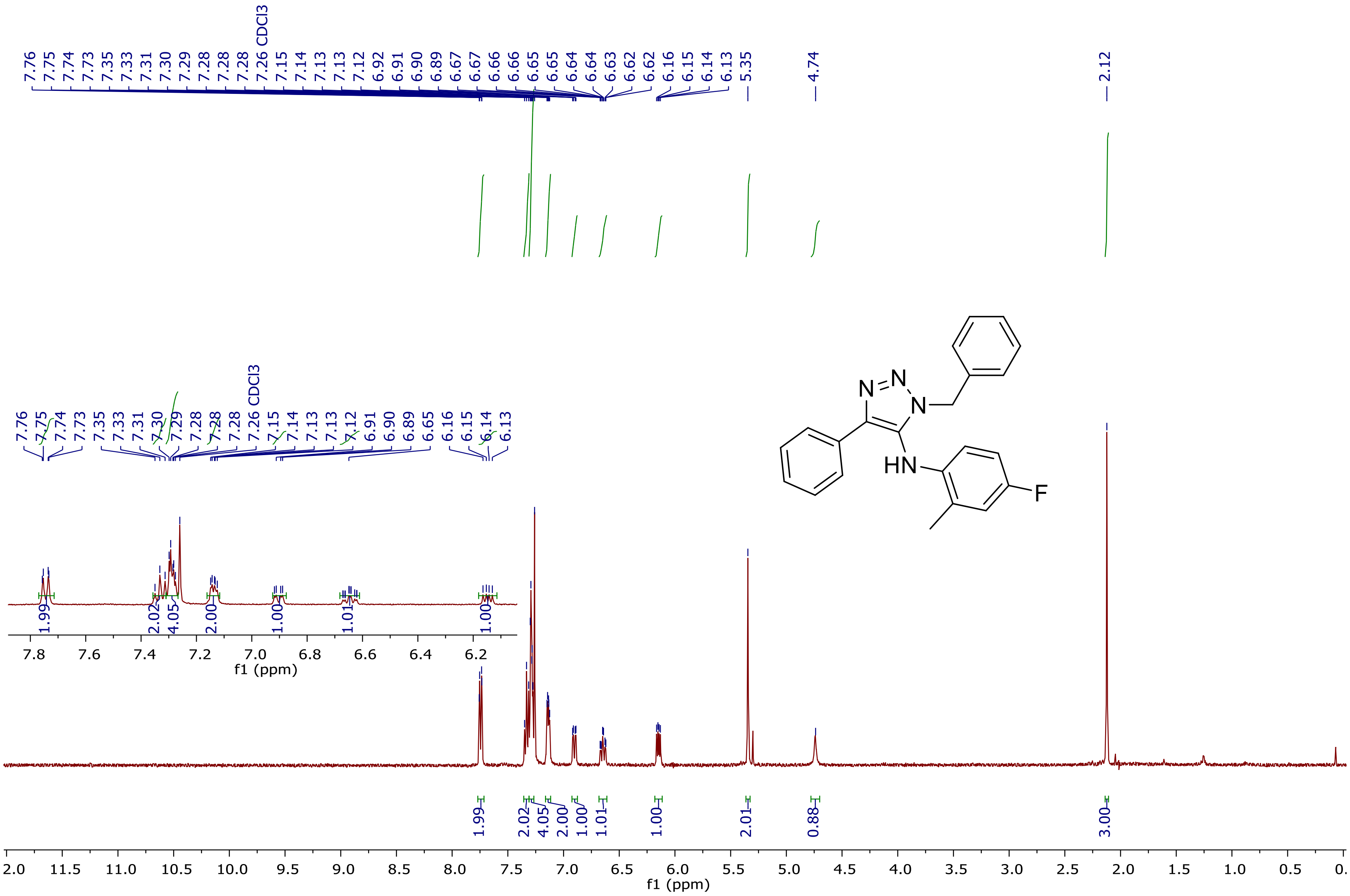

Figure S13. ^{1}H NMR (400 MHz, Chloroform-*d*) of 2f

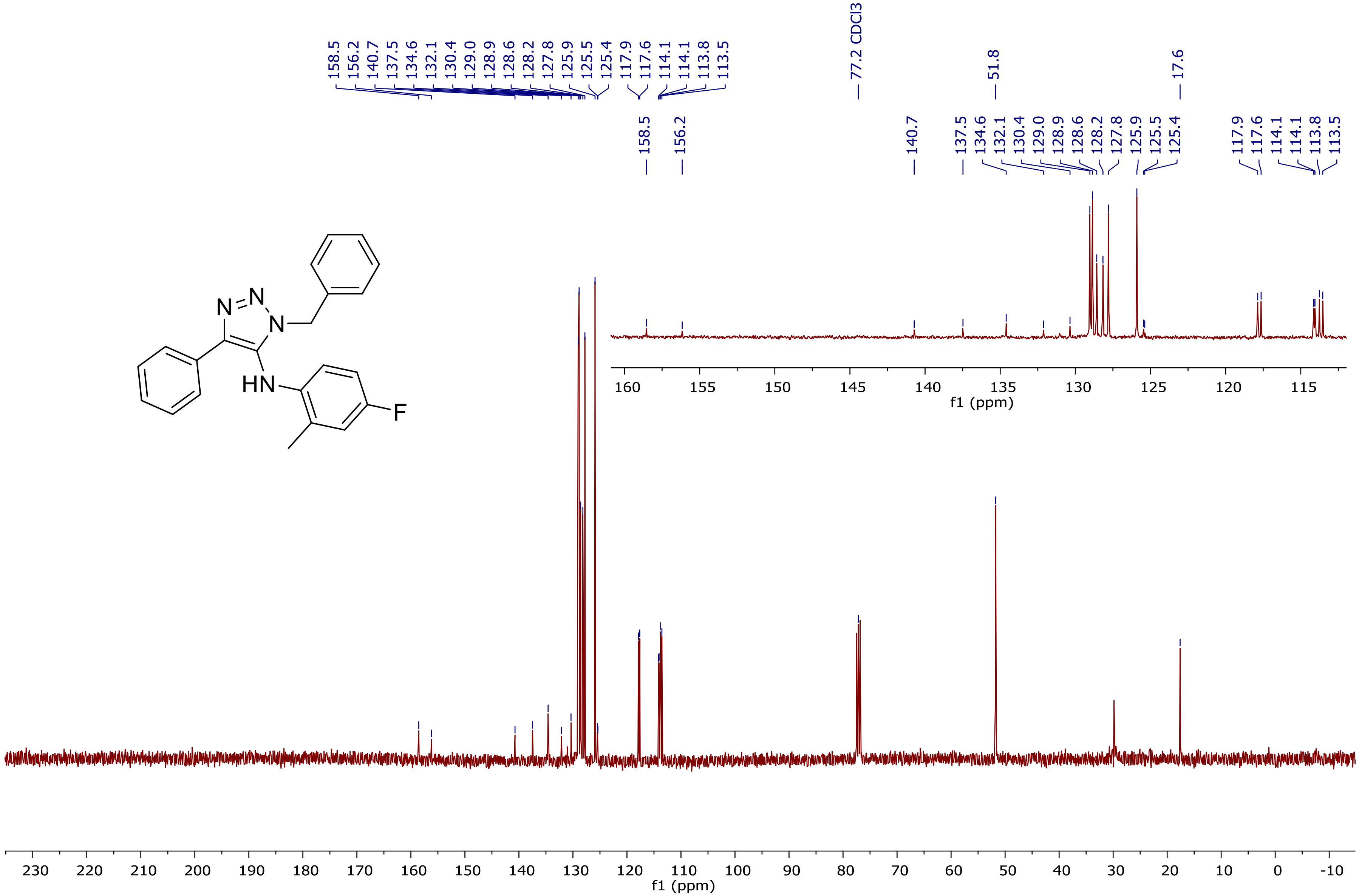

Figure S14. $^{13}C\{^{1}H\}$ **NMR (101 MHz, Chloroform-*d*) of 2f**

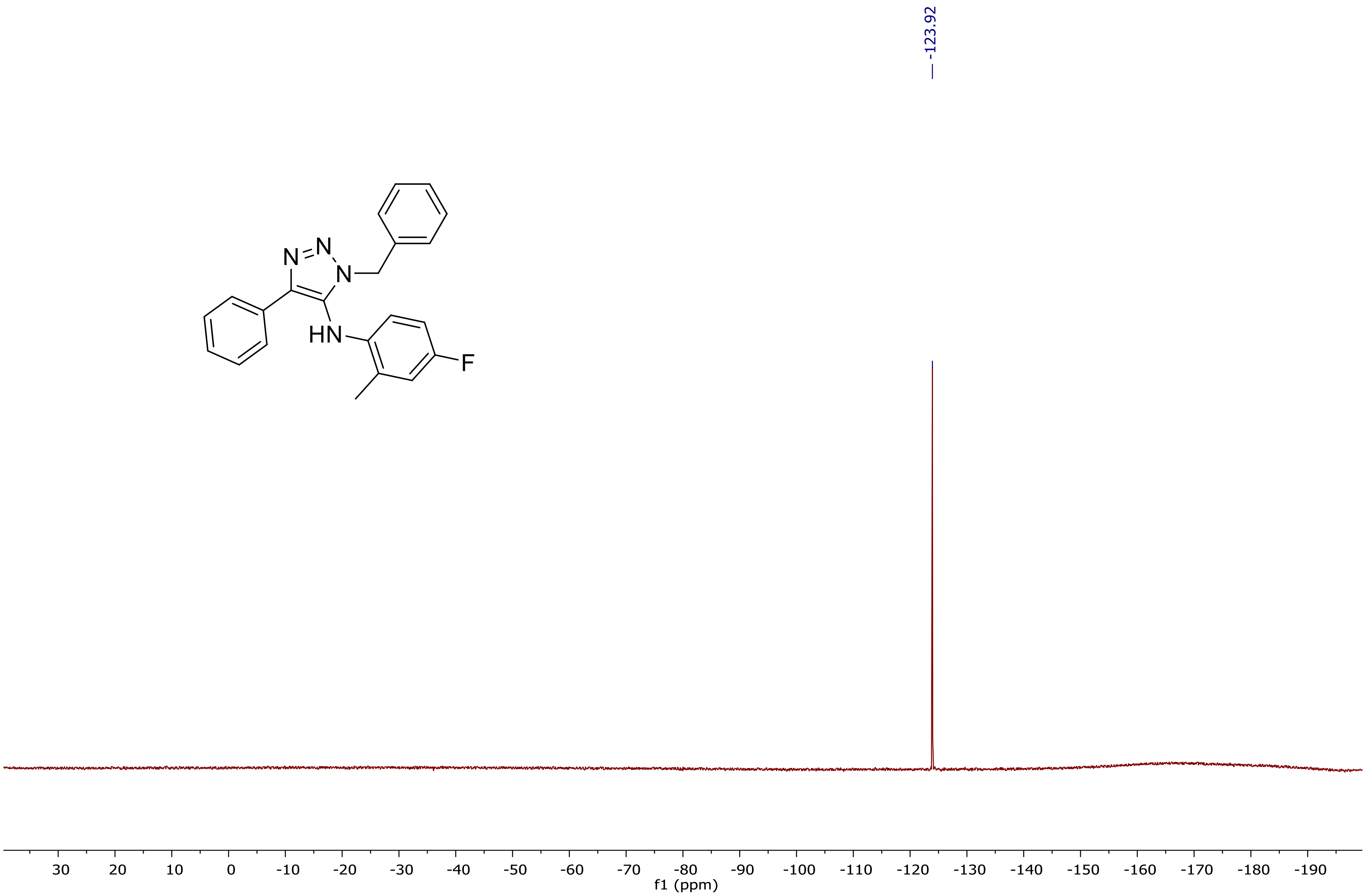

Figure S15. ^{19}**F NMR (376 MHz, Chloroform-*d*) of 2f**

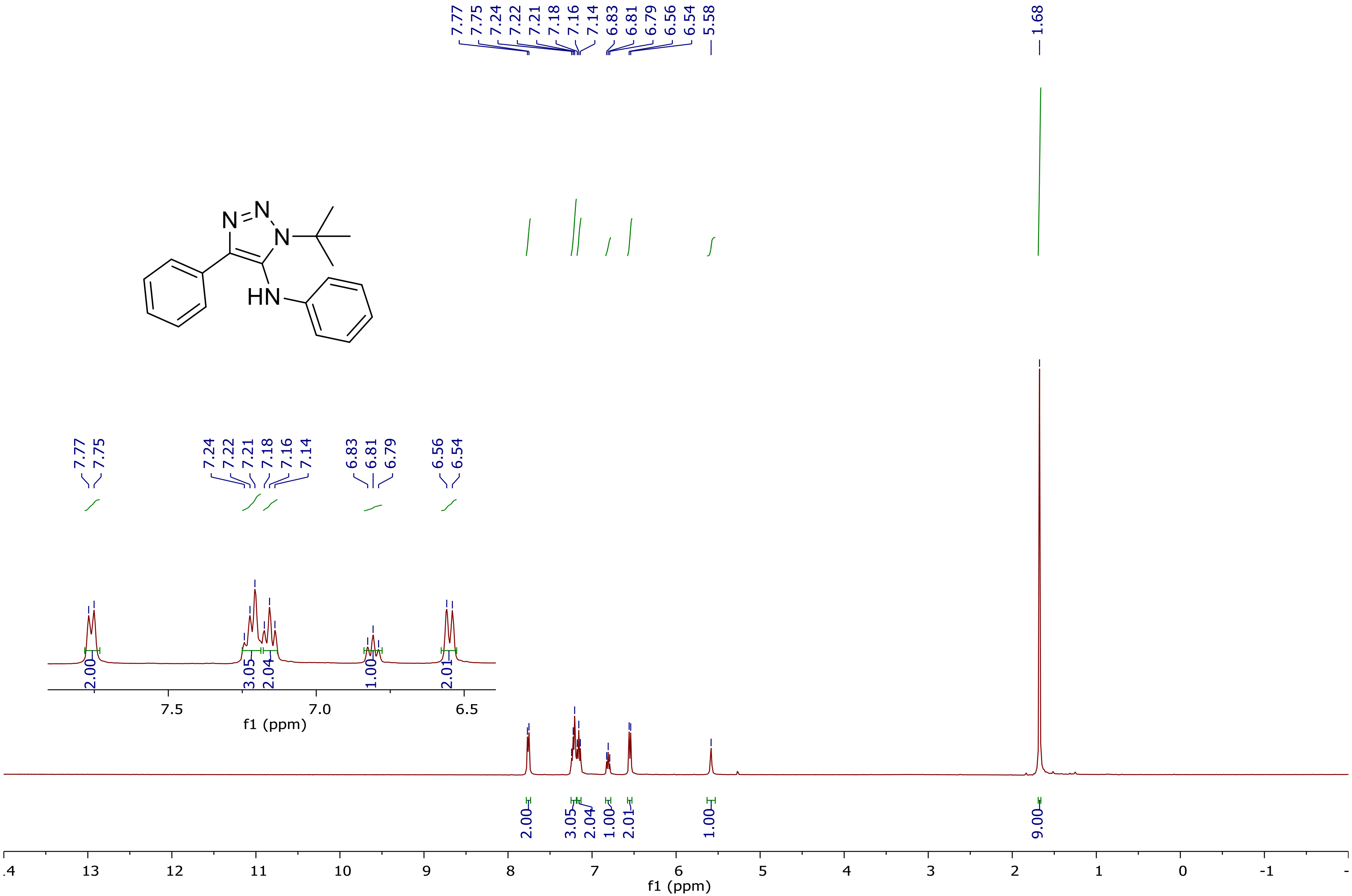

Figure S16. ^{1}H NMR (400 MHz, Chloroform-*d*) of 2g

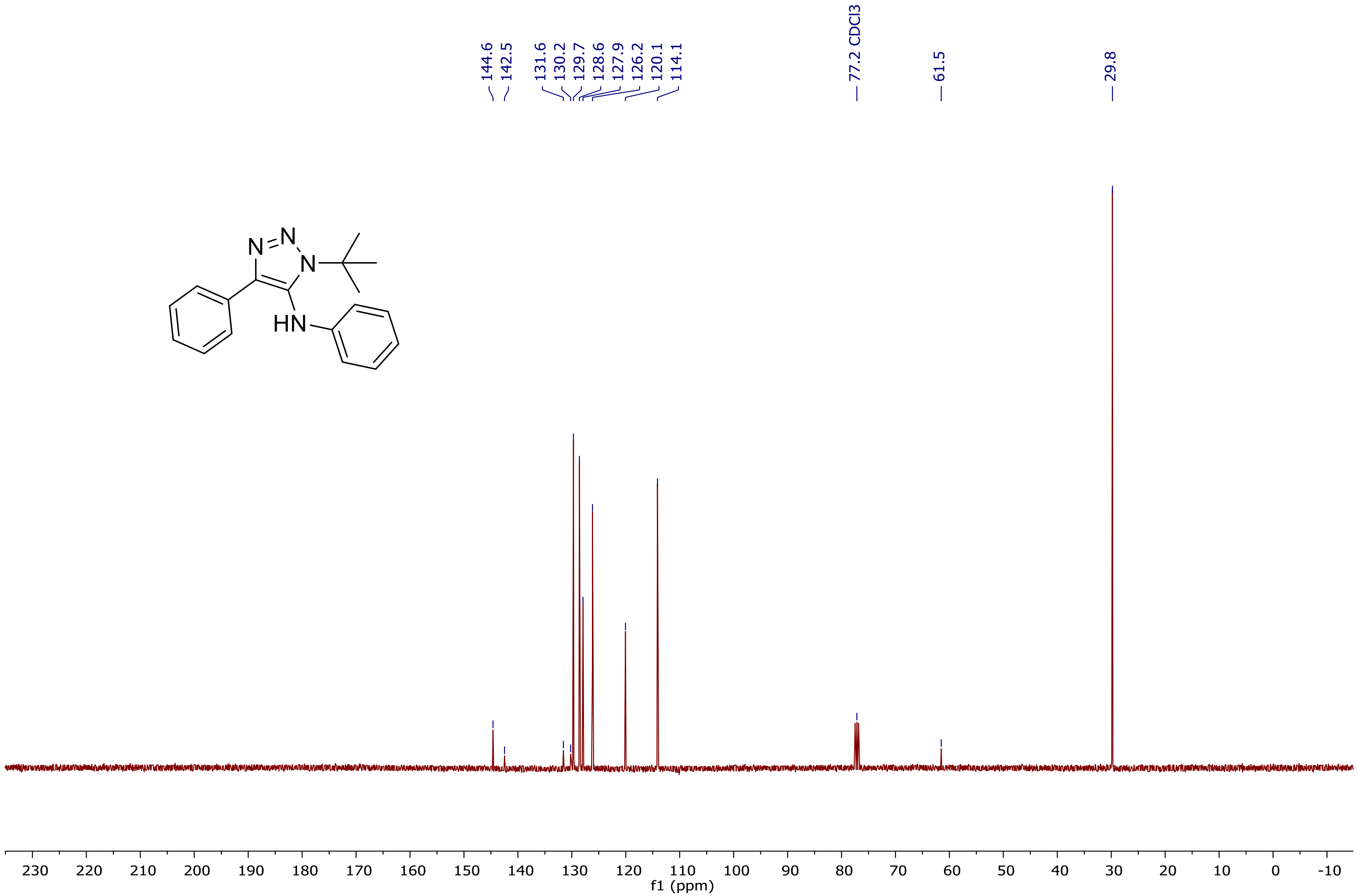

Figure S17. $^{13}C\{^{1}H\}$ **NMR (101 MHz, Chloroform-*d*) of 2g**

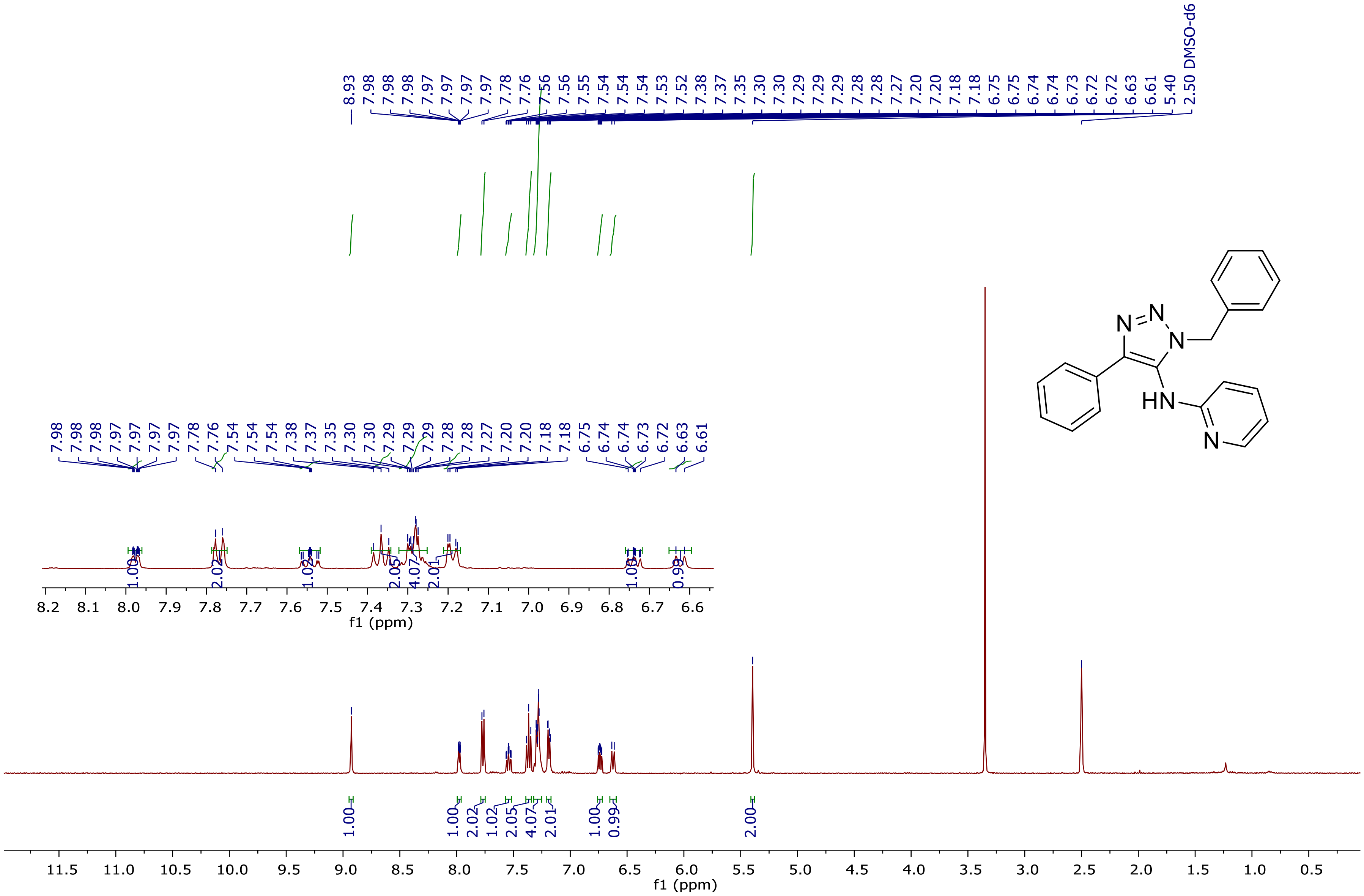

Figure S18. ^{1}H NMR (400 MHz, DMSO-d_6) of 2h

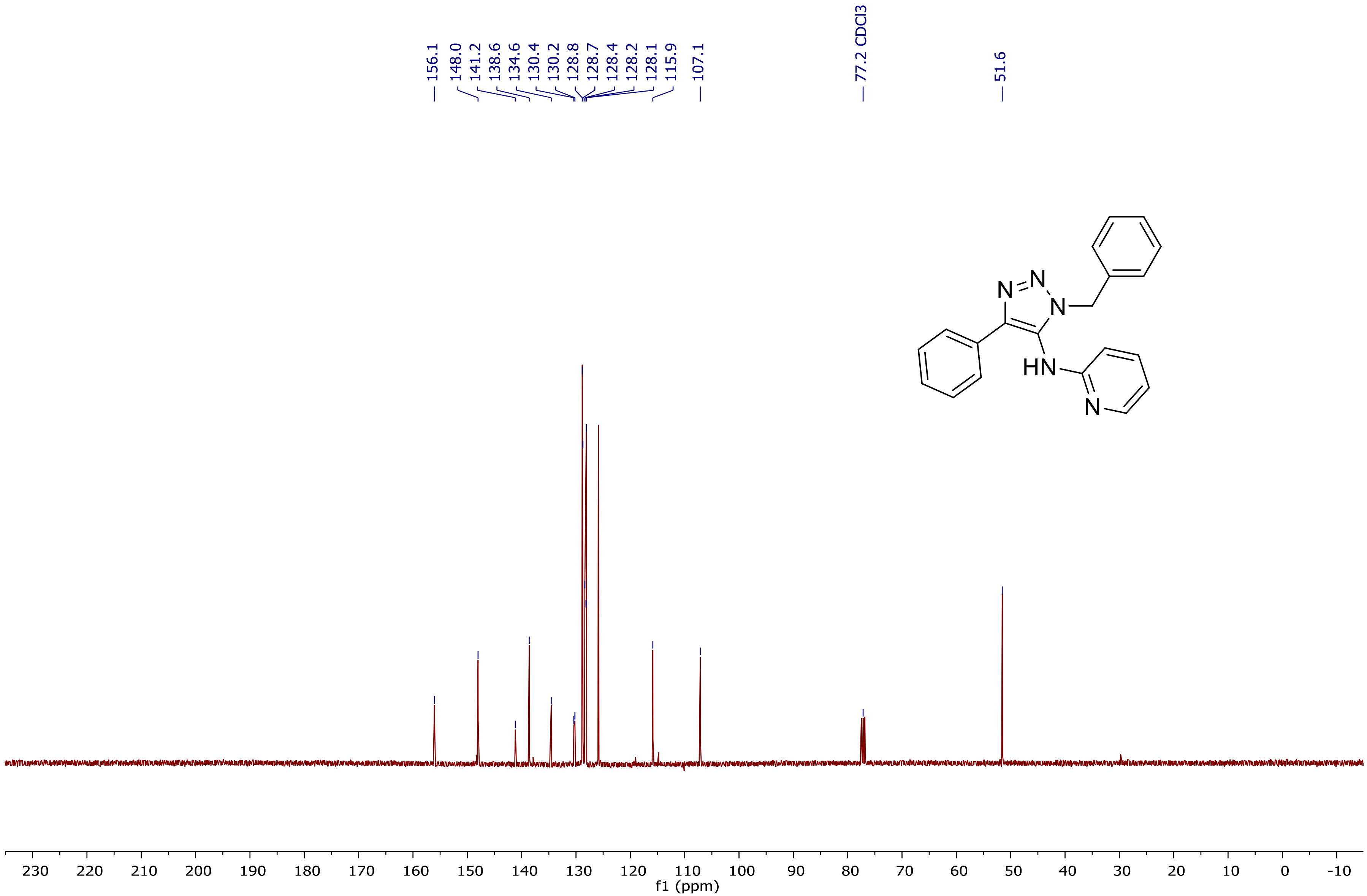

Figure S19. $^{13}C\{^{1}H\}$ **NMR (101 MHz, Chloroform-*d*) of 2h**

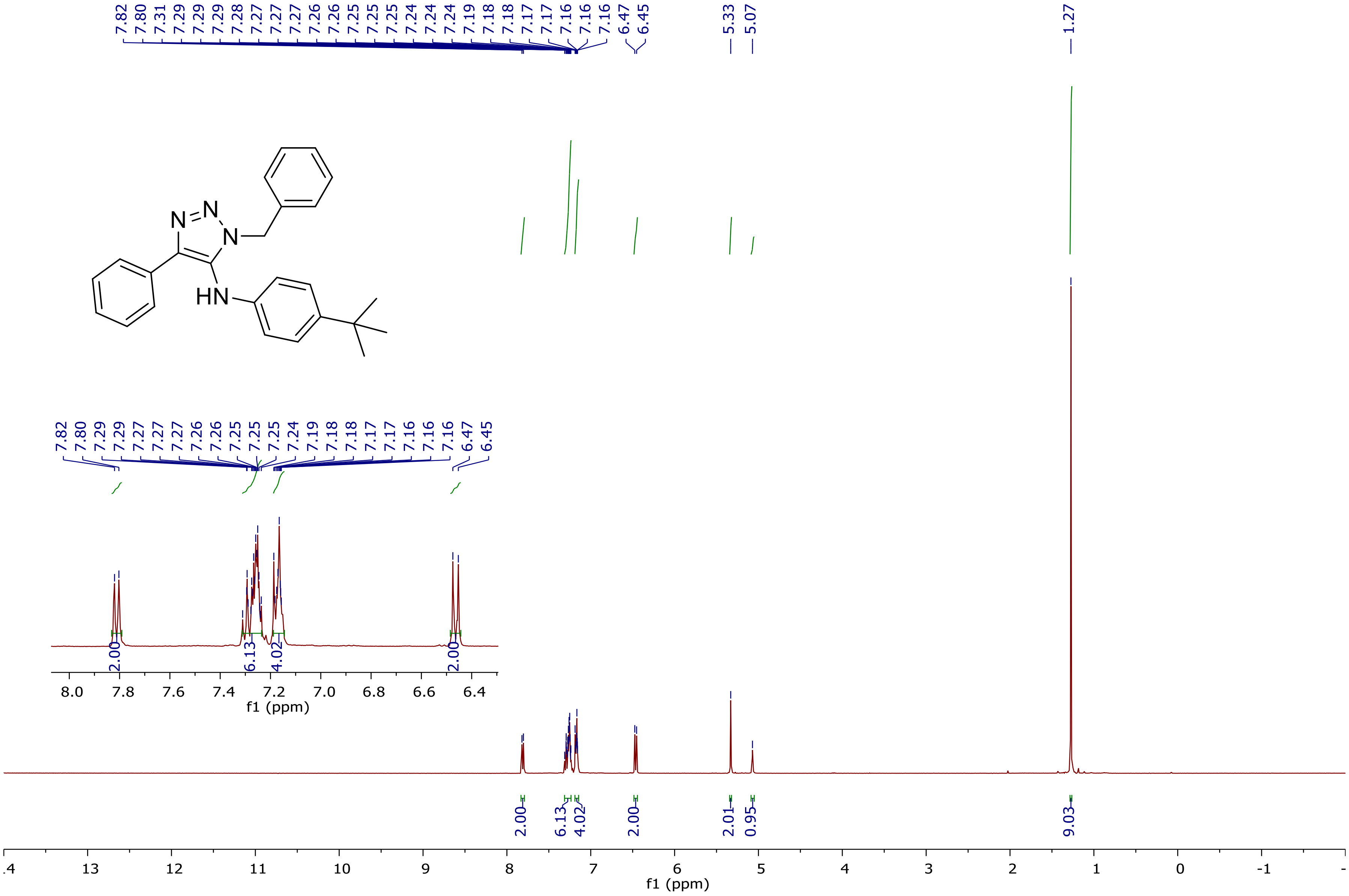

Figure S20. ^{1}H NMR (400 MHz, Chloroform-*d*) of 2i

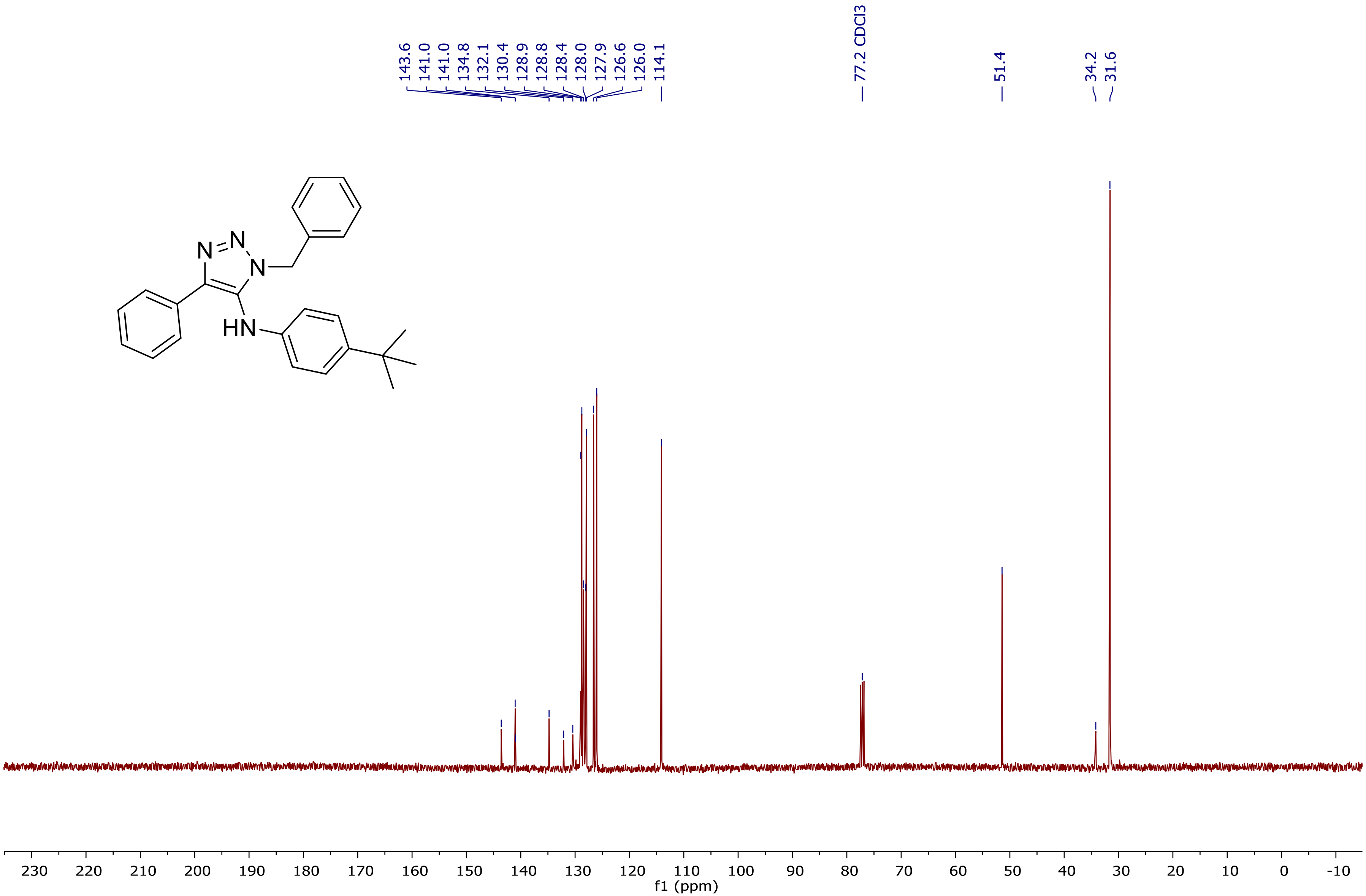

Figure S21. $^{13}C\{^1H\}$ NMR (101 MHz, Chloroform-*d*) of 2i

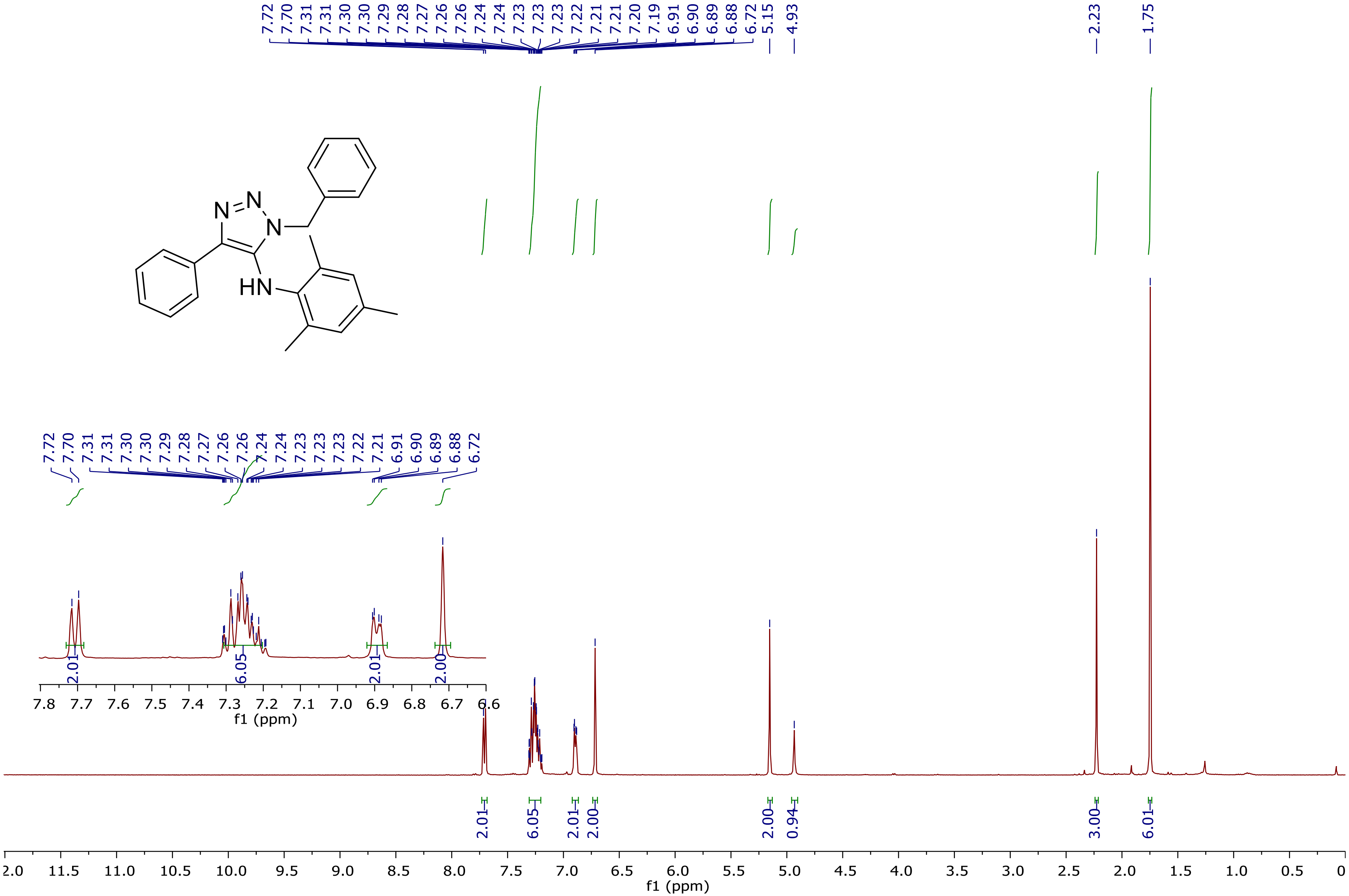

Figure S22. ^{1}H NMR (400 MHz, Chloroform-*d*) of 2j

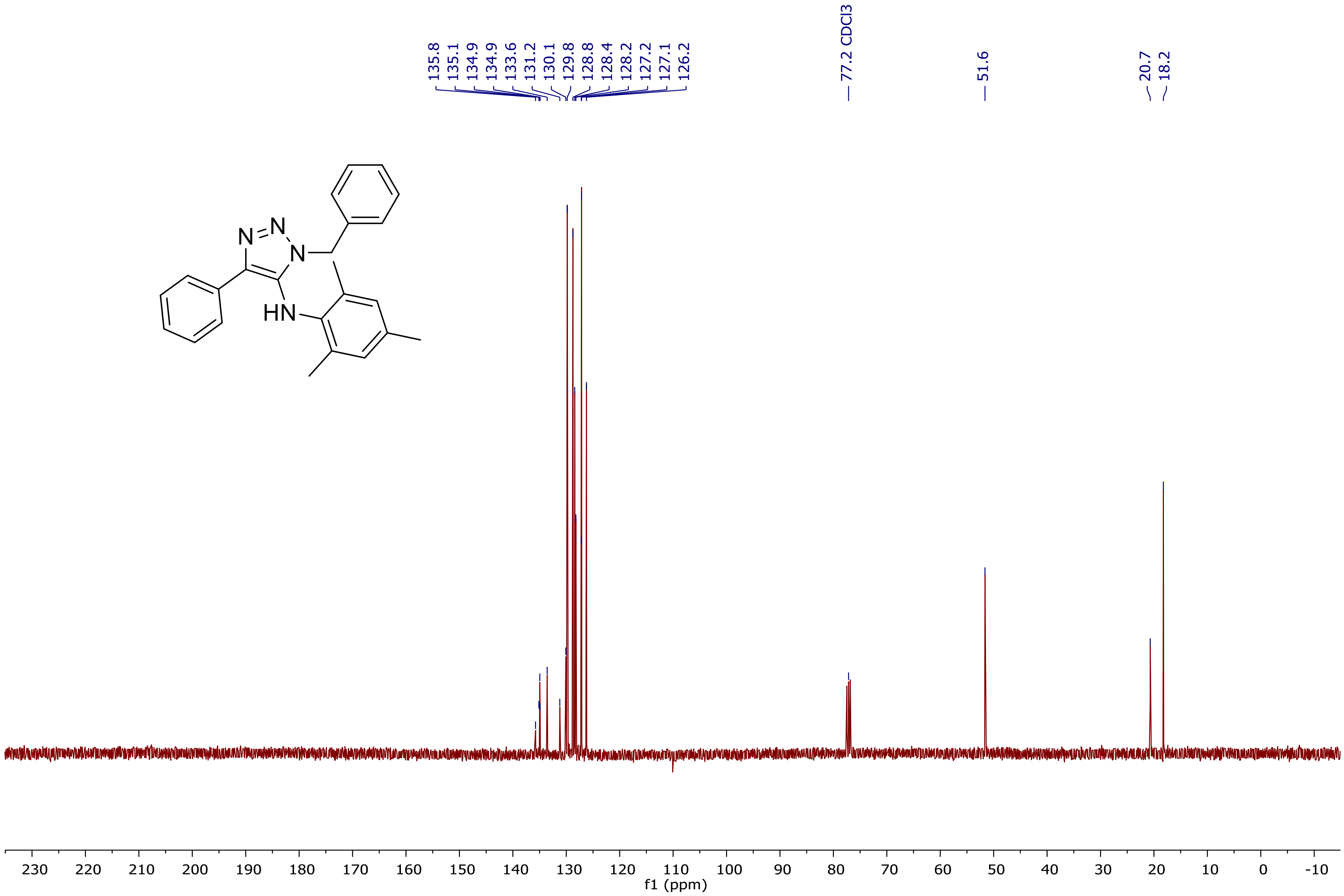

Figure S23. $^{13}C\{^{1}H\}$ **NMR (101 MHz, Chloroform-*d*) of 2j**

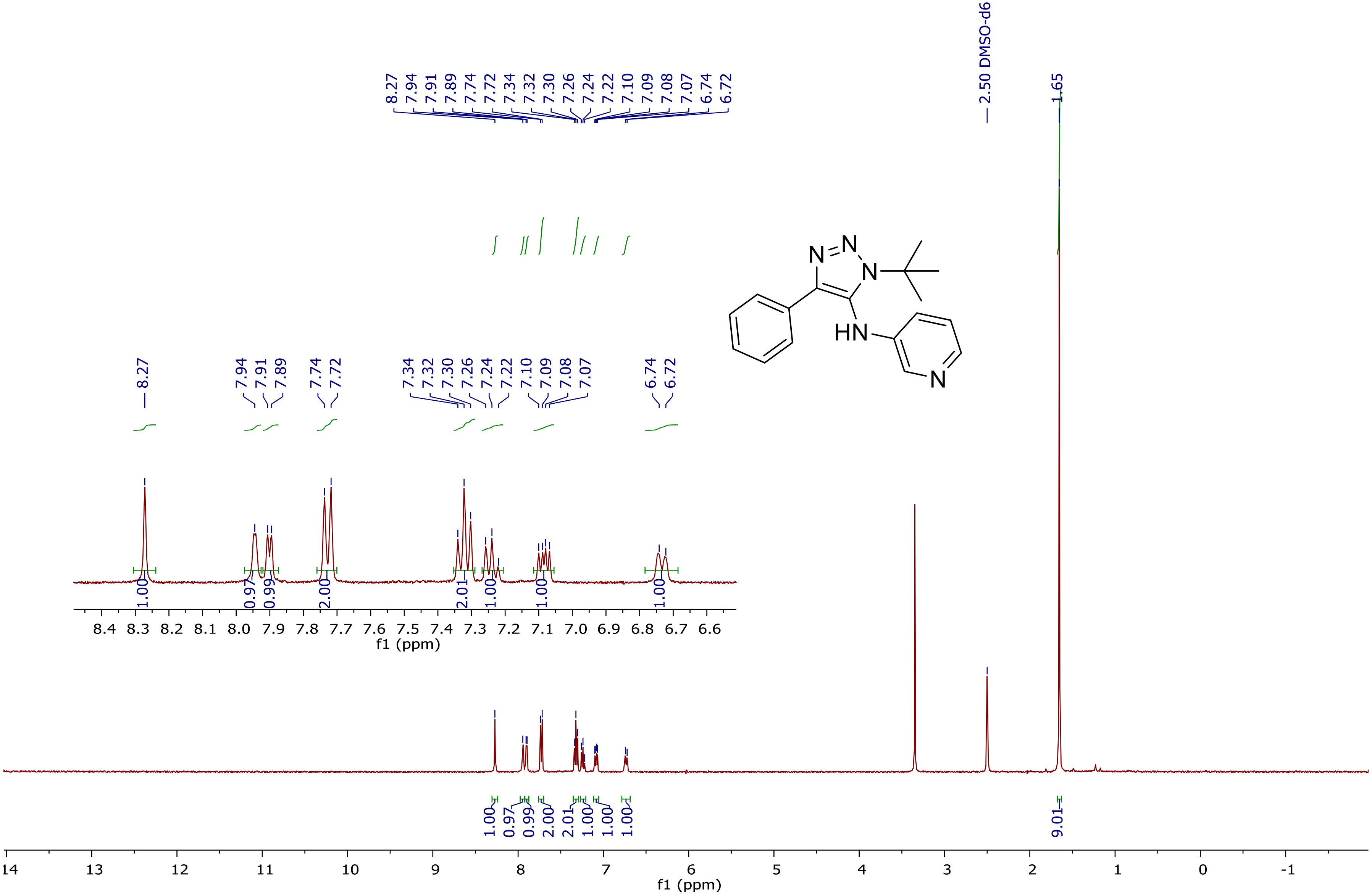

Figure S24. ^{1}**H NMR (400 MHz, DMSO-**d_6**) of 2k**

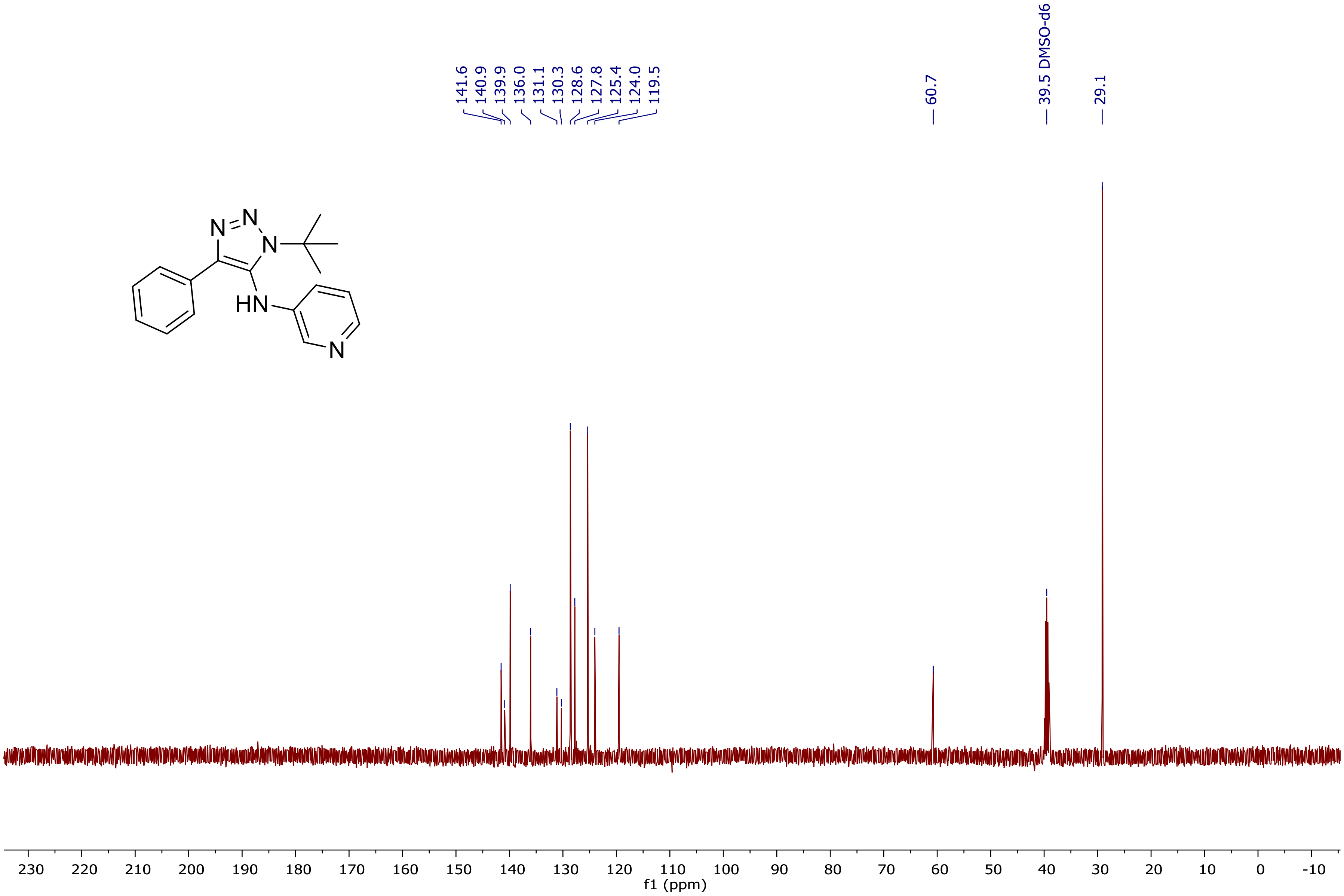

Figure S25. $^{13}C\{^{1}H\}$ **NMR (101 MHz, DMSO-d_6) of 2k**

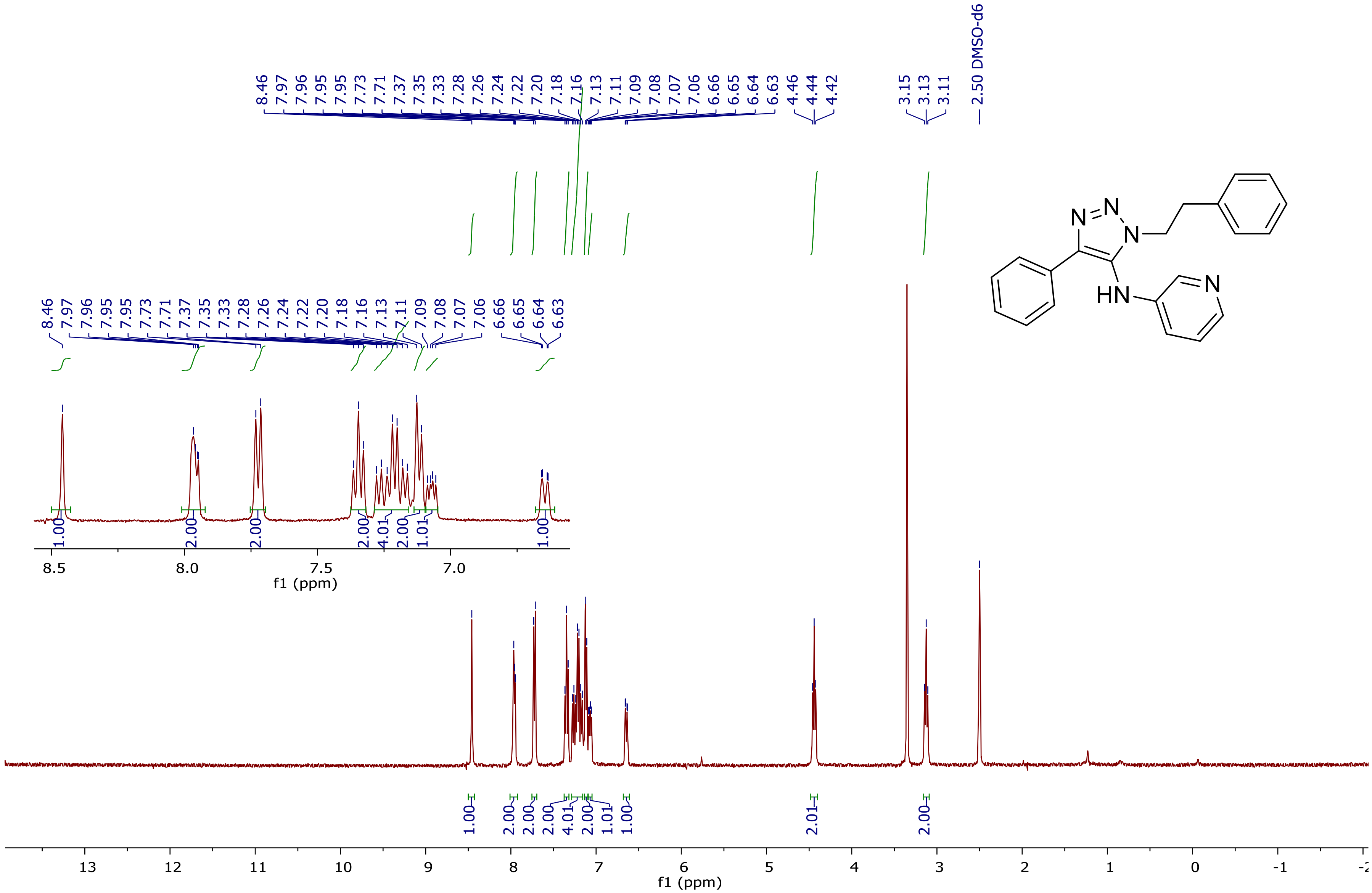

Figure S26. ^{1}H NMR (400 MHz, DMSO-d_6) of 2l

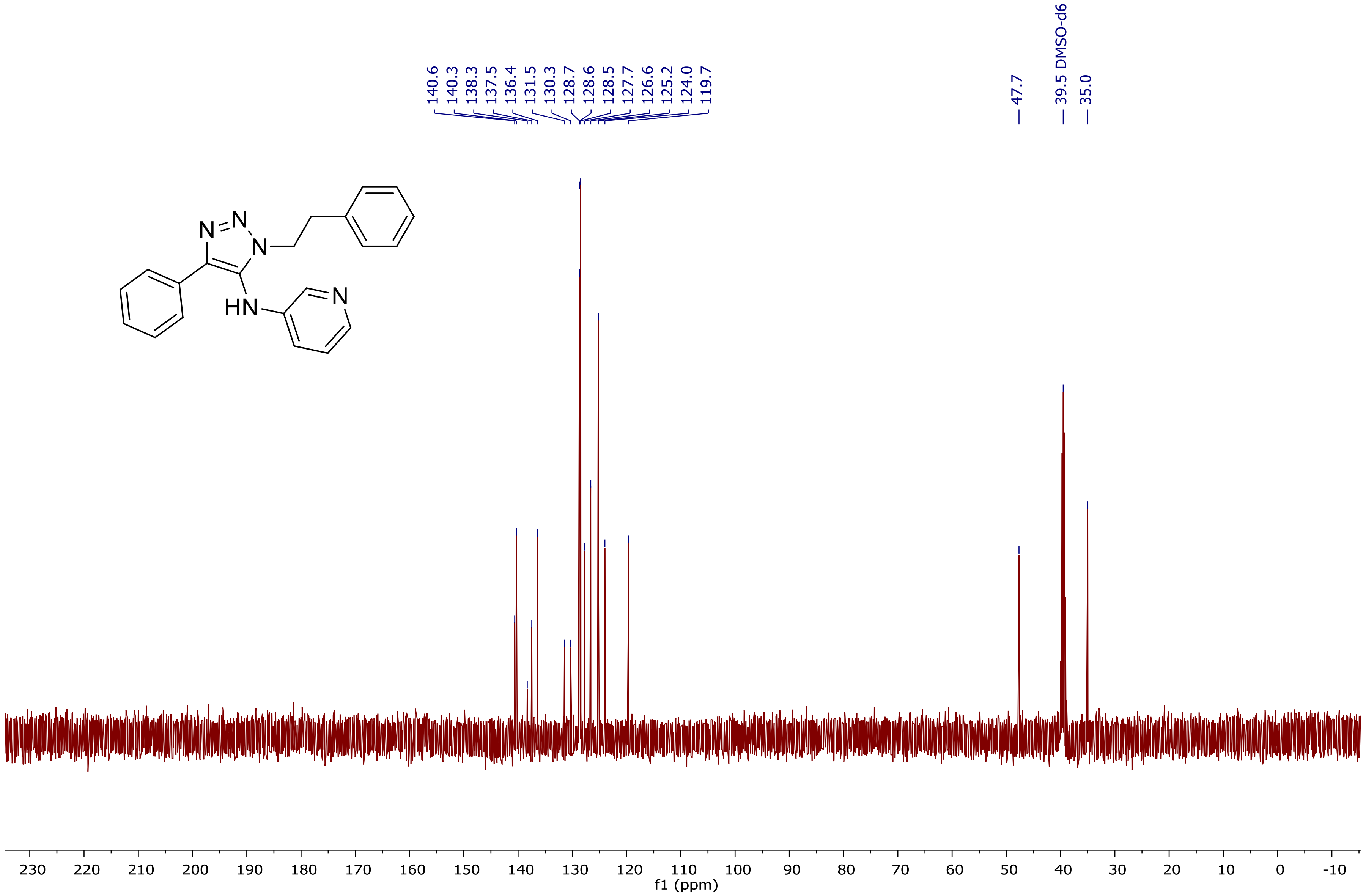

Figure S27. $^{13}C\{^{1}H\}$ **NMR (101 MHz, DMSO-d_6) of 2l**

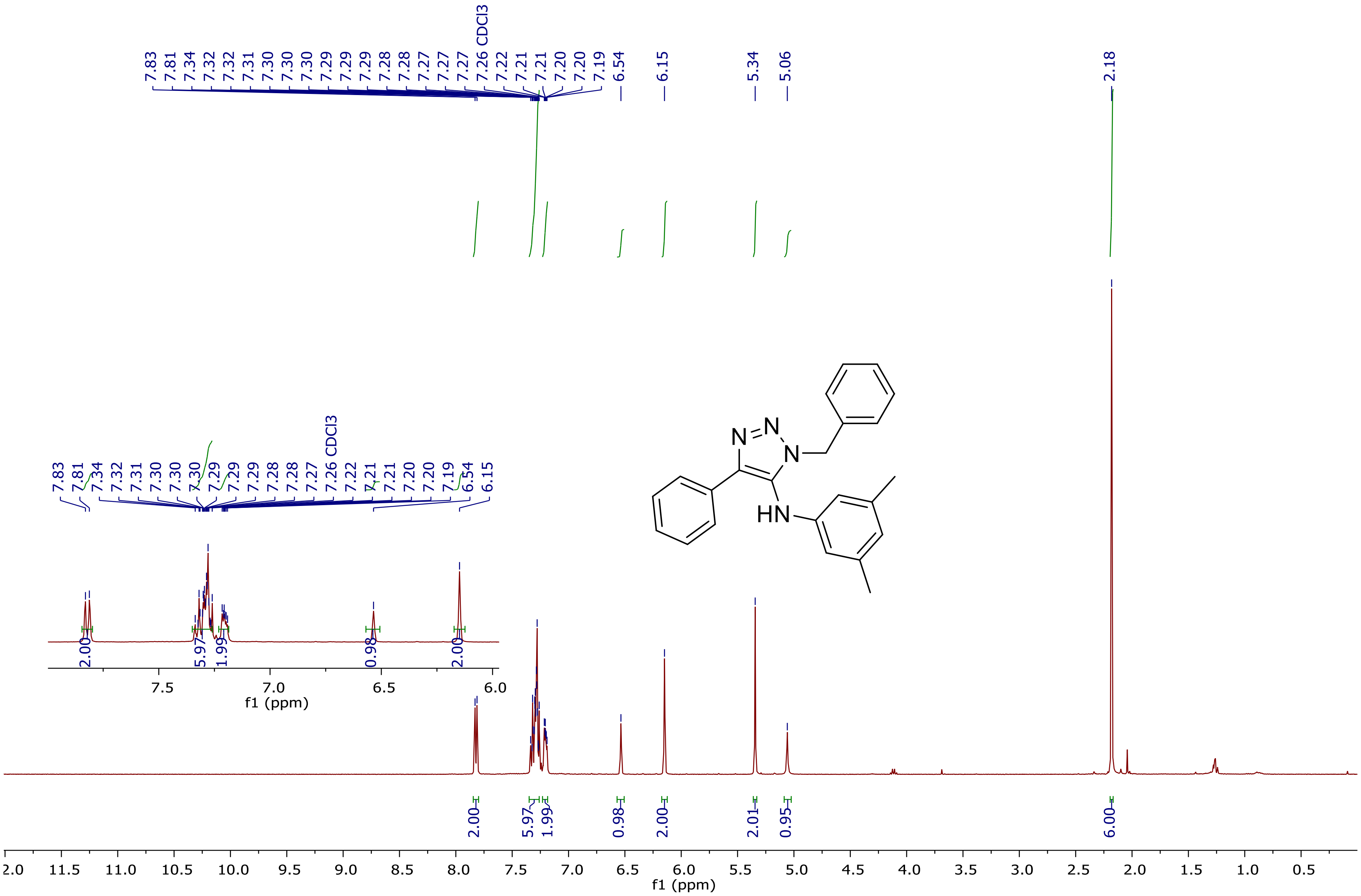

Figure S28. ^{1}H NMR (400 MHz, Chloroform-*d*) of 2m

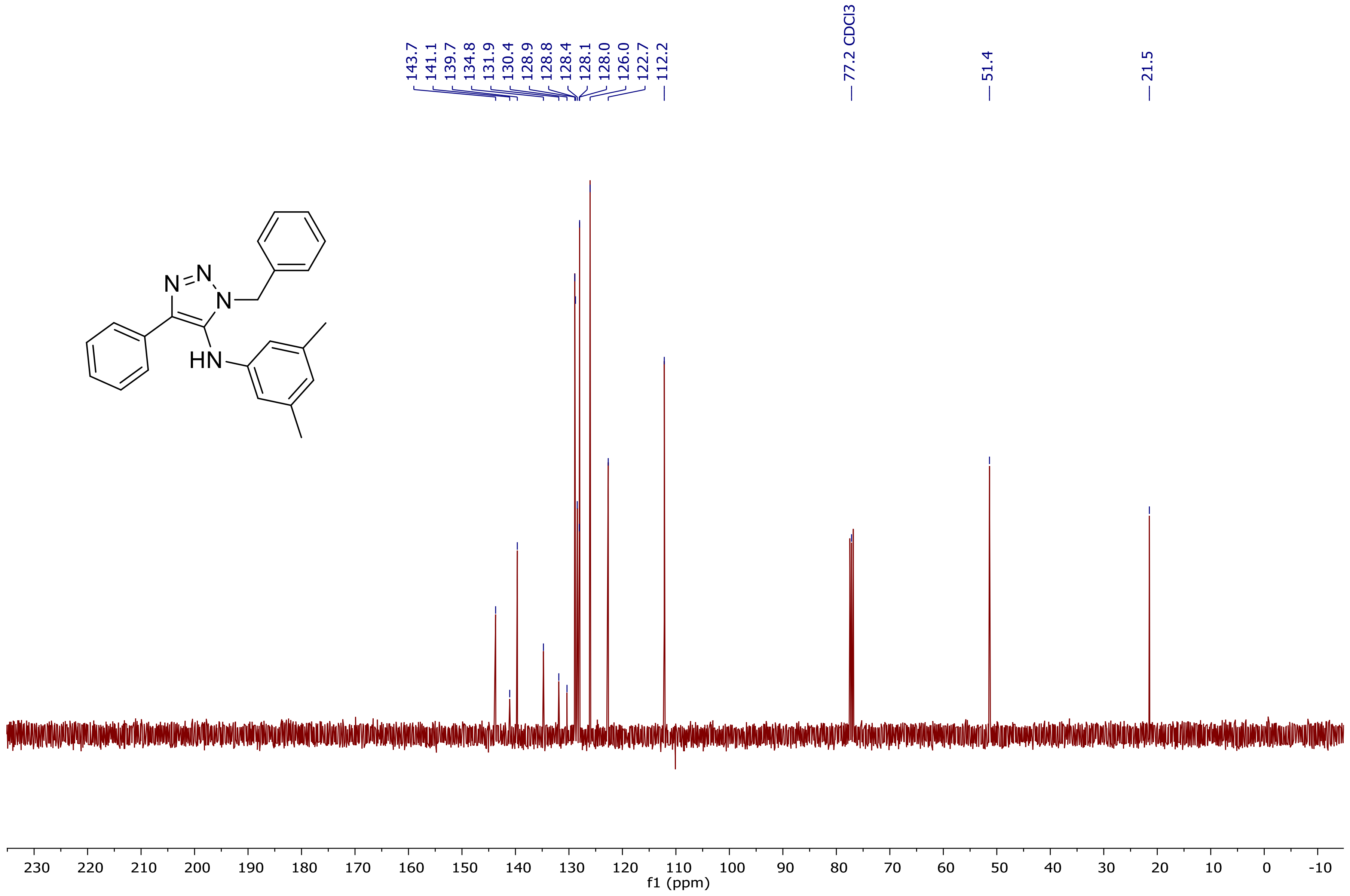

Figure S29. $^{13}C\{^{1}H\}$ **NMR (101 MHz, Chloroform-*d*) of 2m**

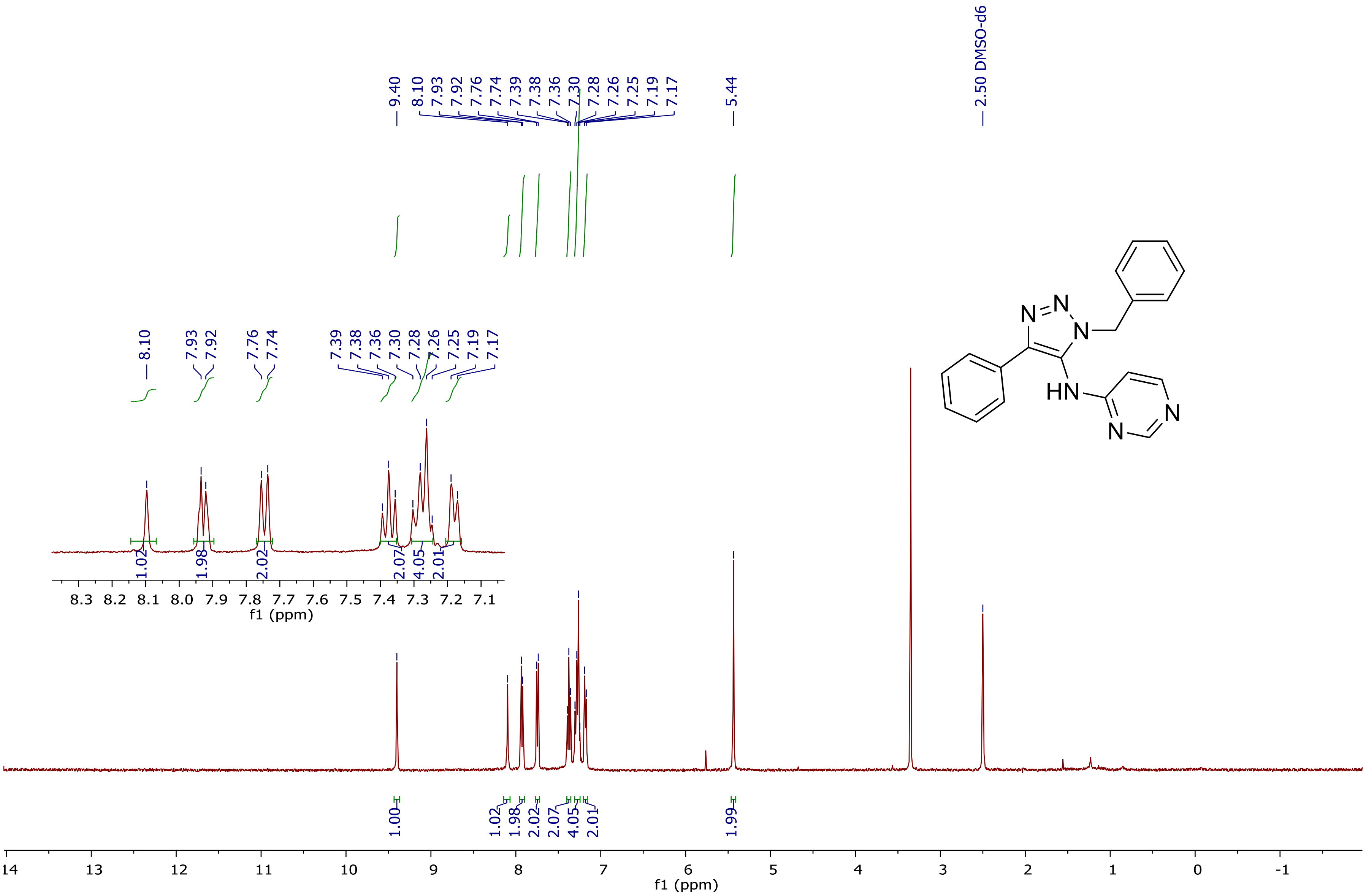

Figure S30. ^{1}**H NMR (400 MHz, DMSO-*d*$_6$) of 2n**

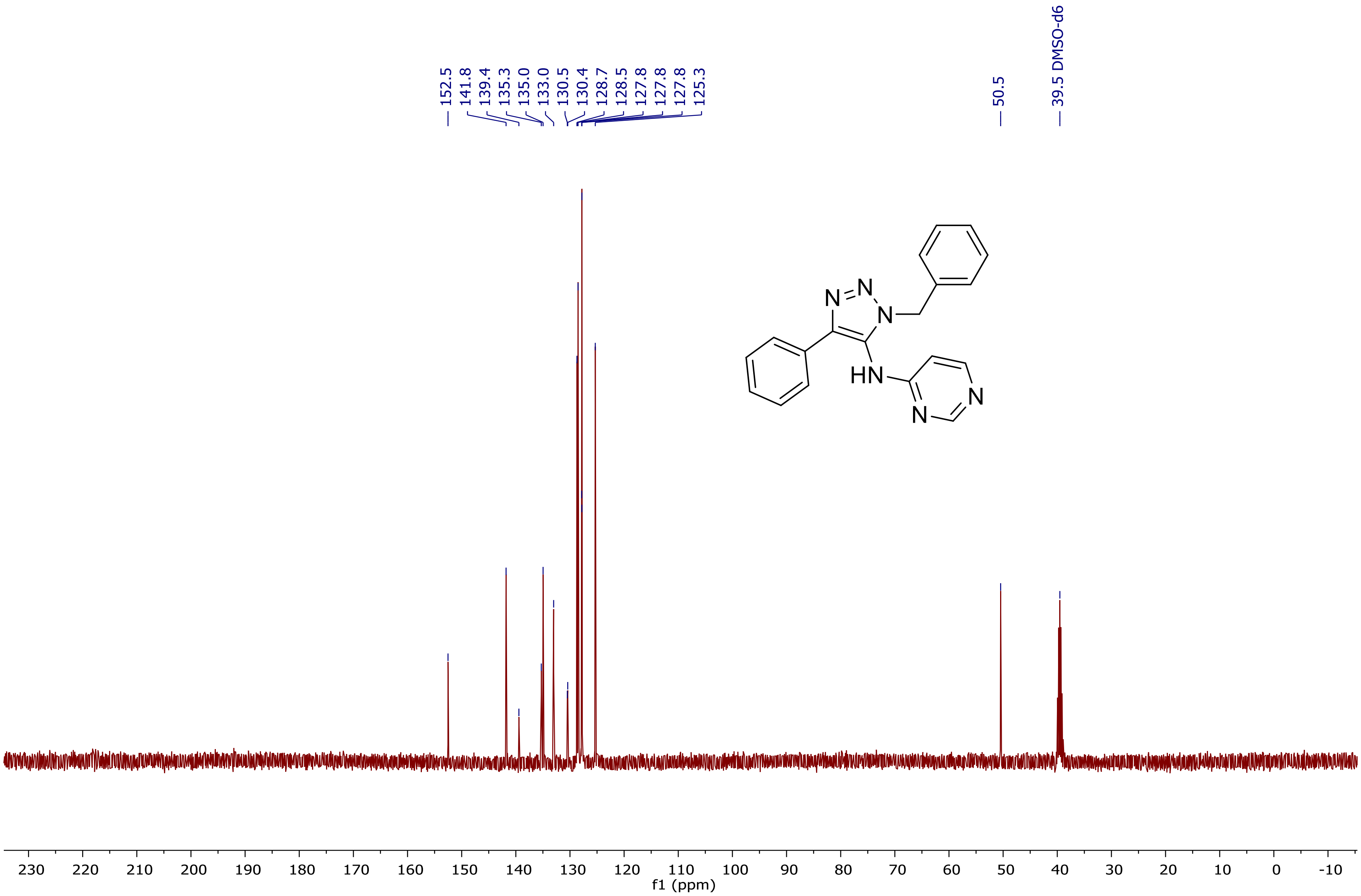

Figure S31. $^{13}C\{^{1}H\}$ **NMR (101 MHz, DMSO-d_6) of 2n**

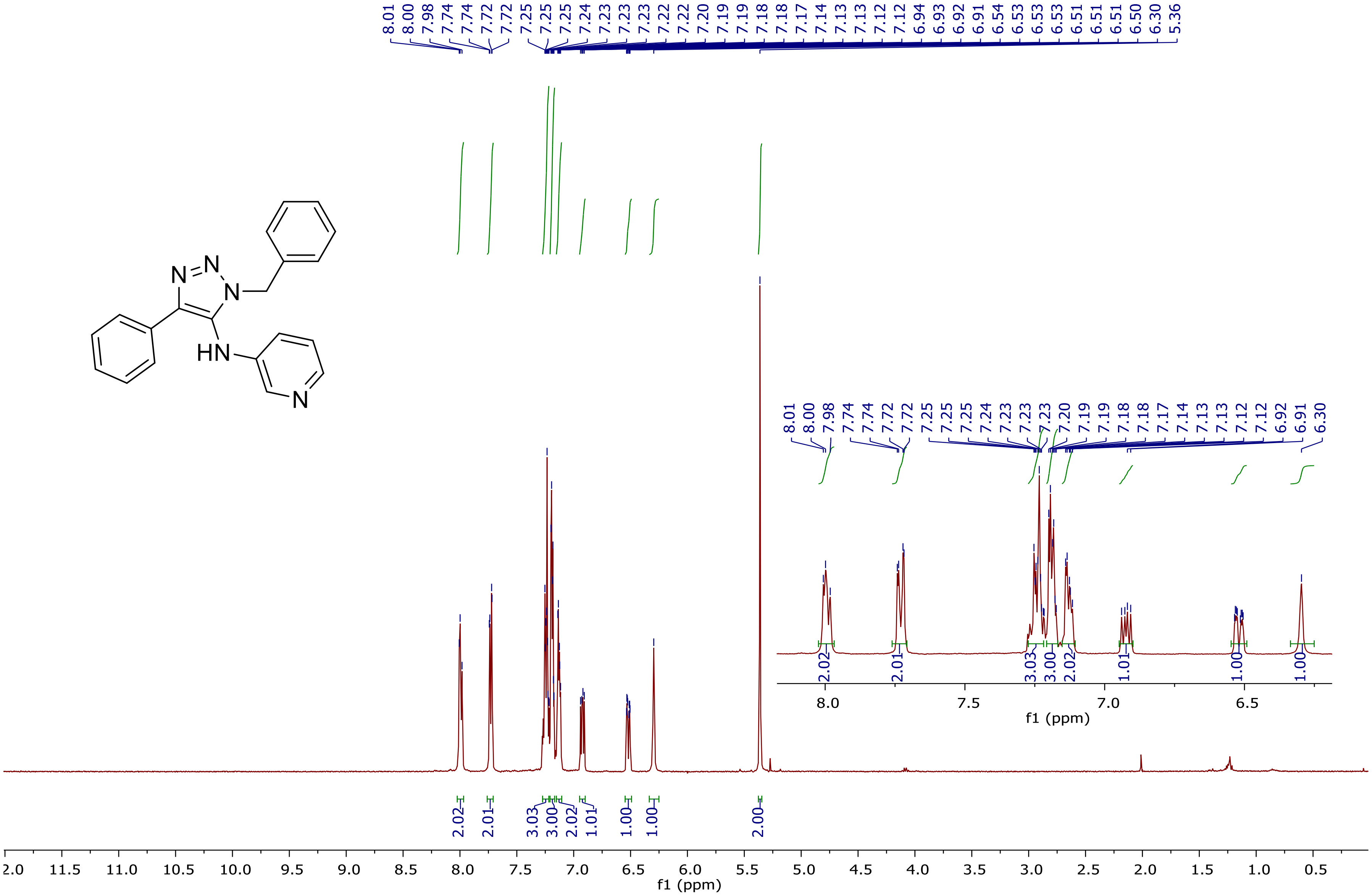

Figure S32. ^{1}H NMR (400 MHz, Chloroform-*d*) of 2o

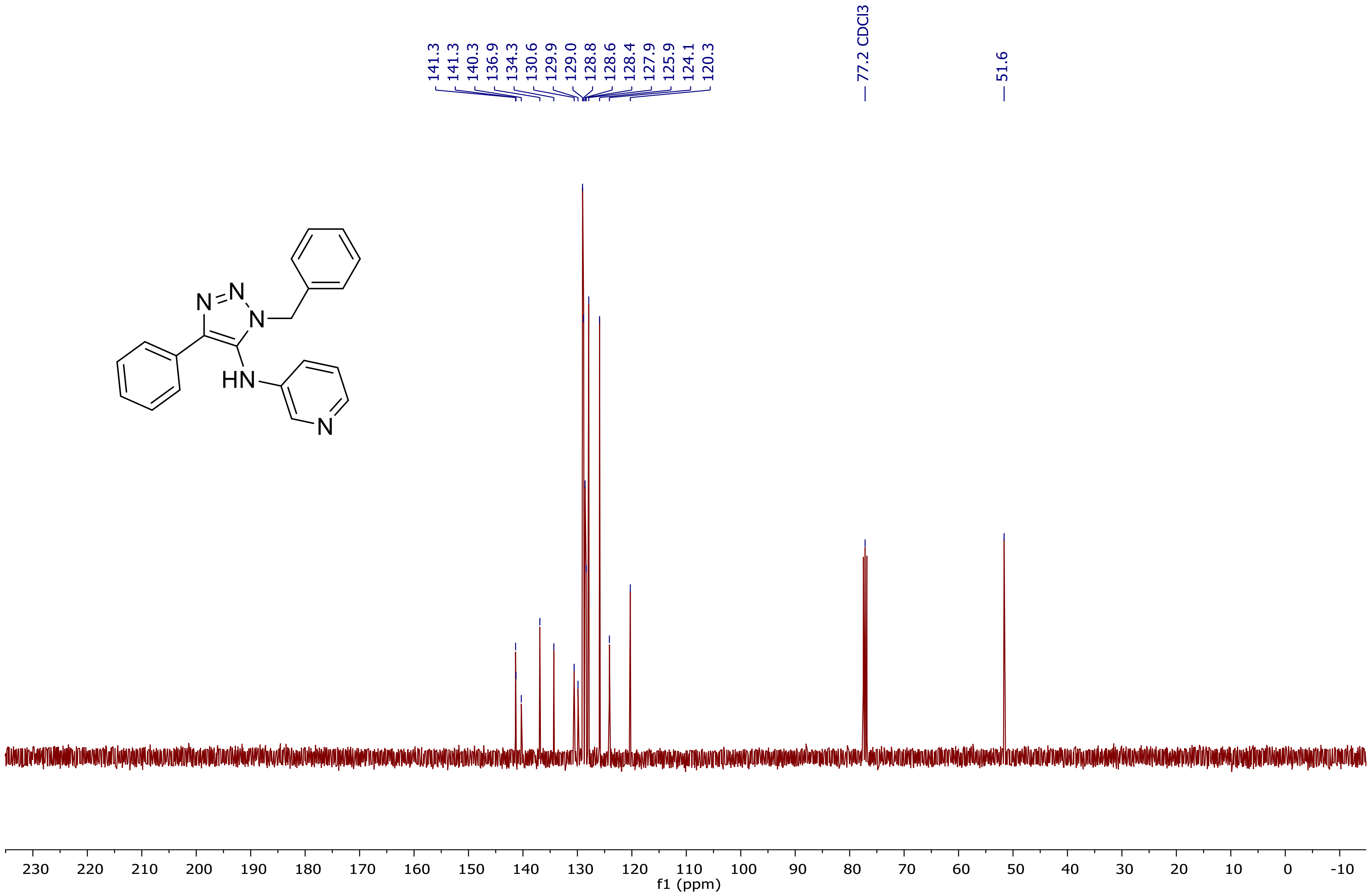

Figure S33. $^{13}C\{^{1}H\}$ **NMR (101 MHz, Chloroform-*d*) of 2o**

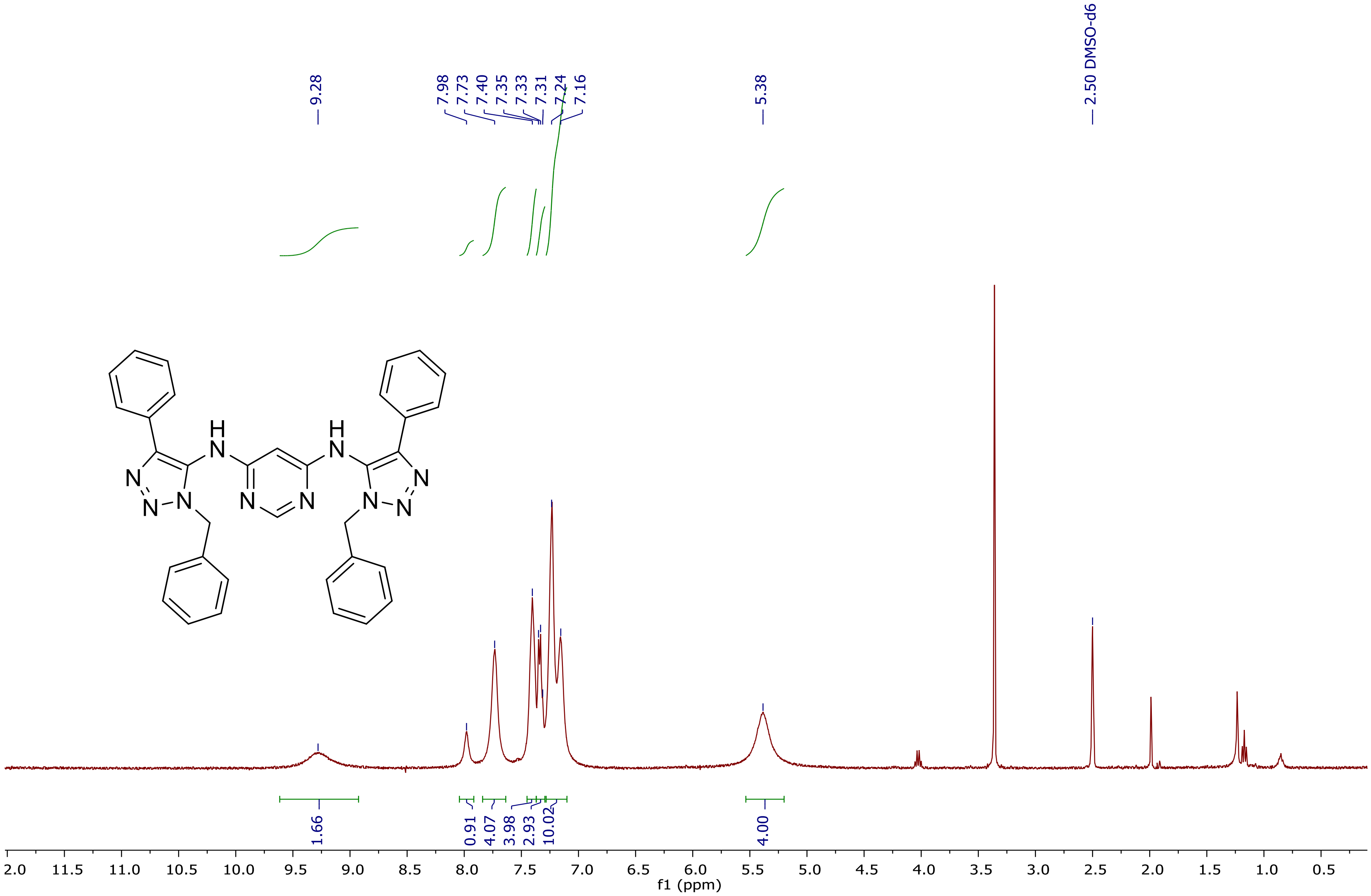

Figure S34. ^{1}H NMR (400 MHz, DMSO-d_6) of 2p

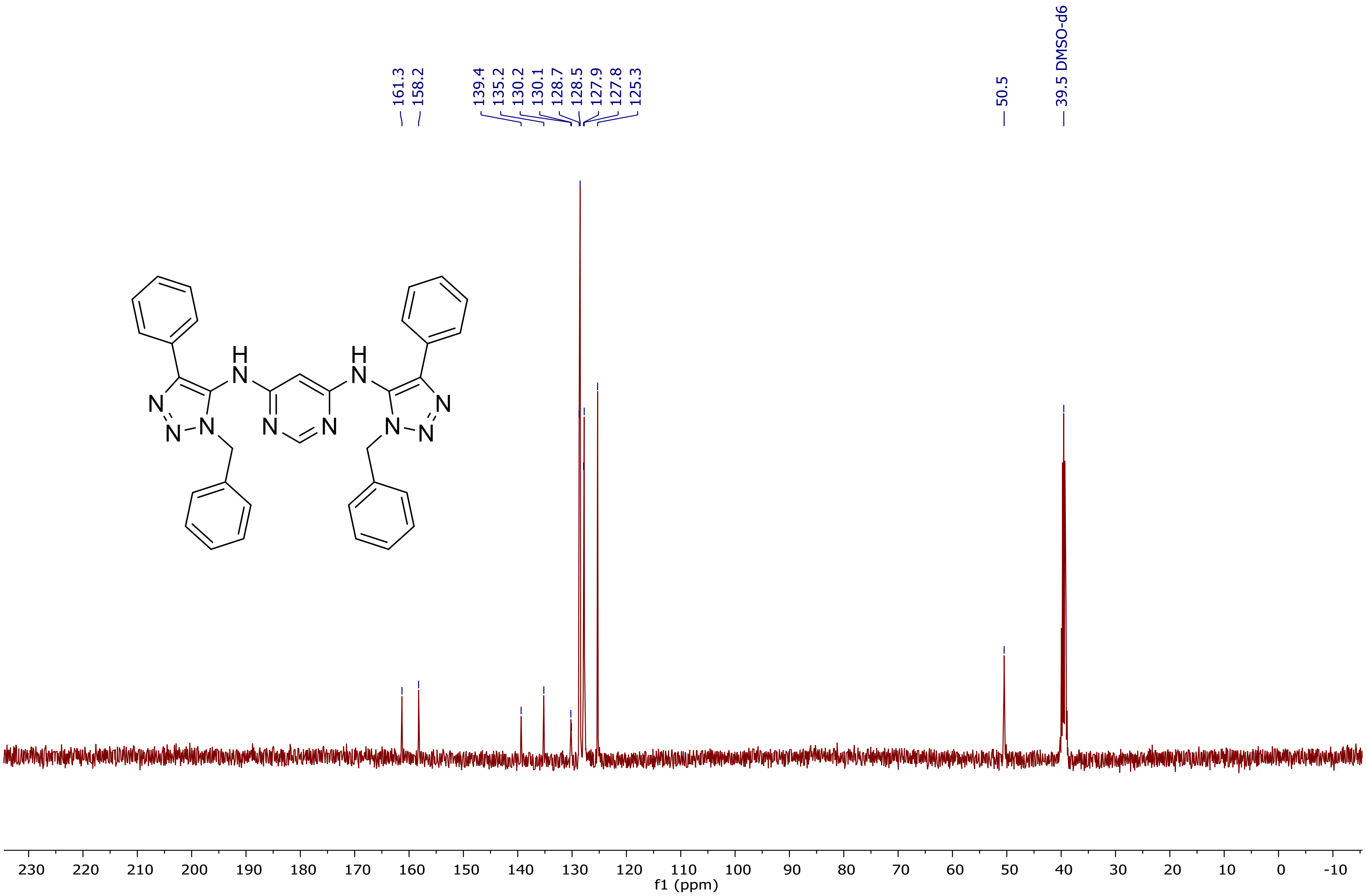

Figure S35. $^{13}C\{^{1}H\}$ NMR (101 MHz, DMSO-d_6) of 2p

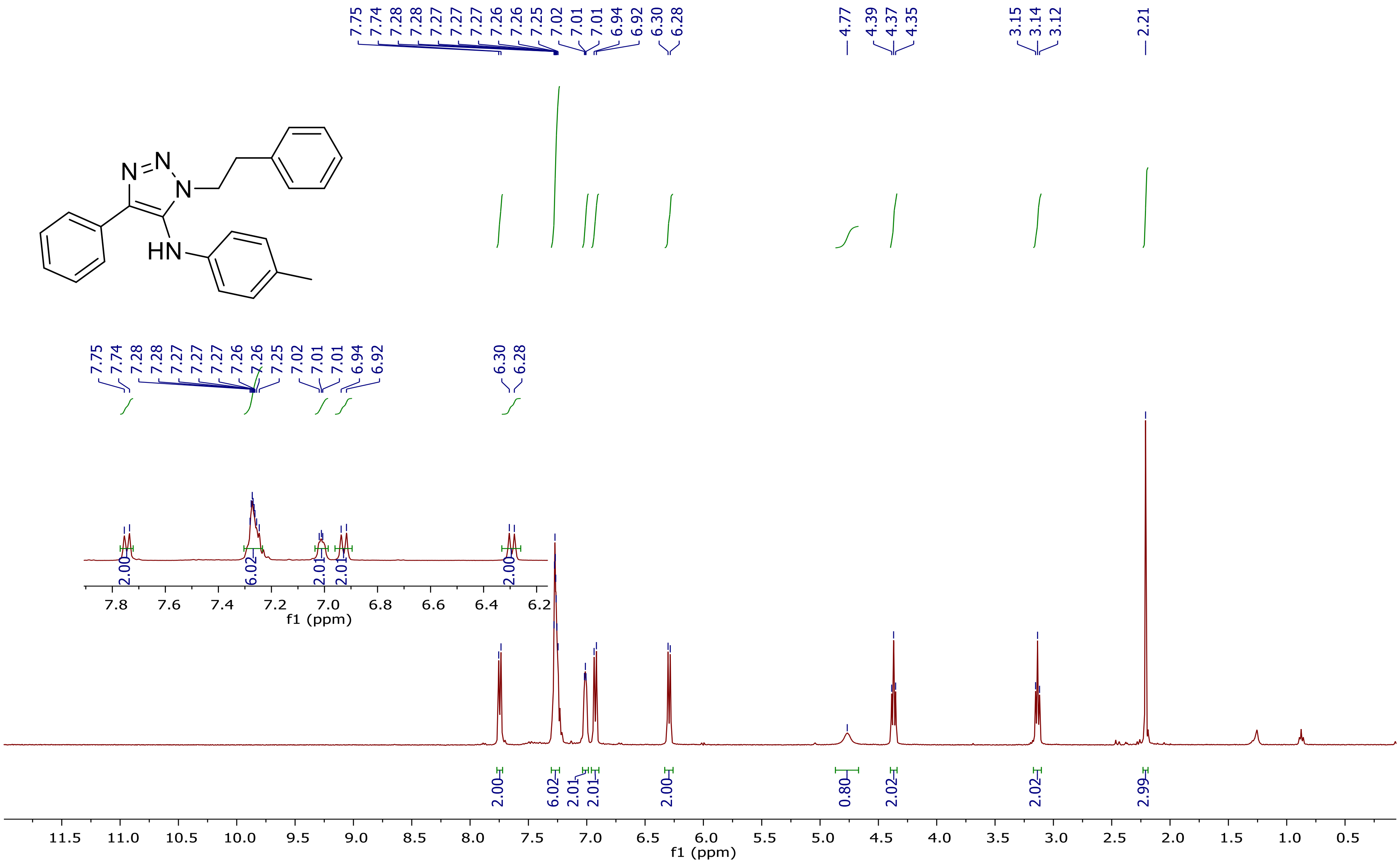

Figure S36. ^{1}H NMR (400 MHz, Chloroform-*d*) of 2q

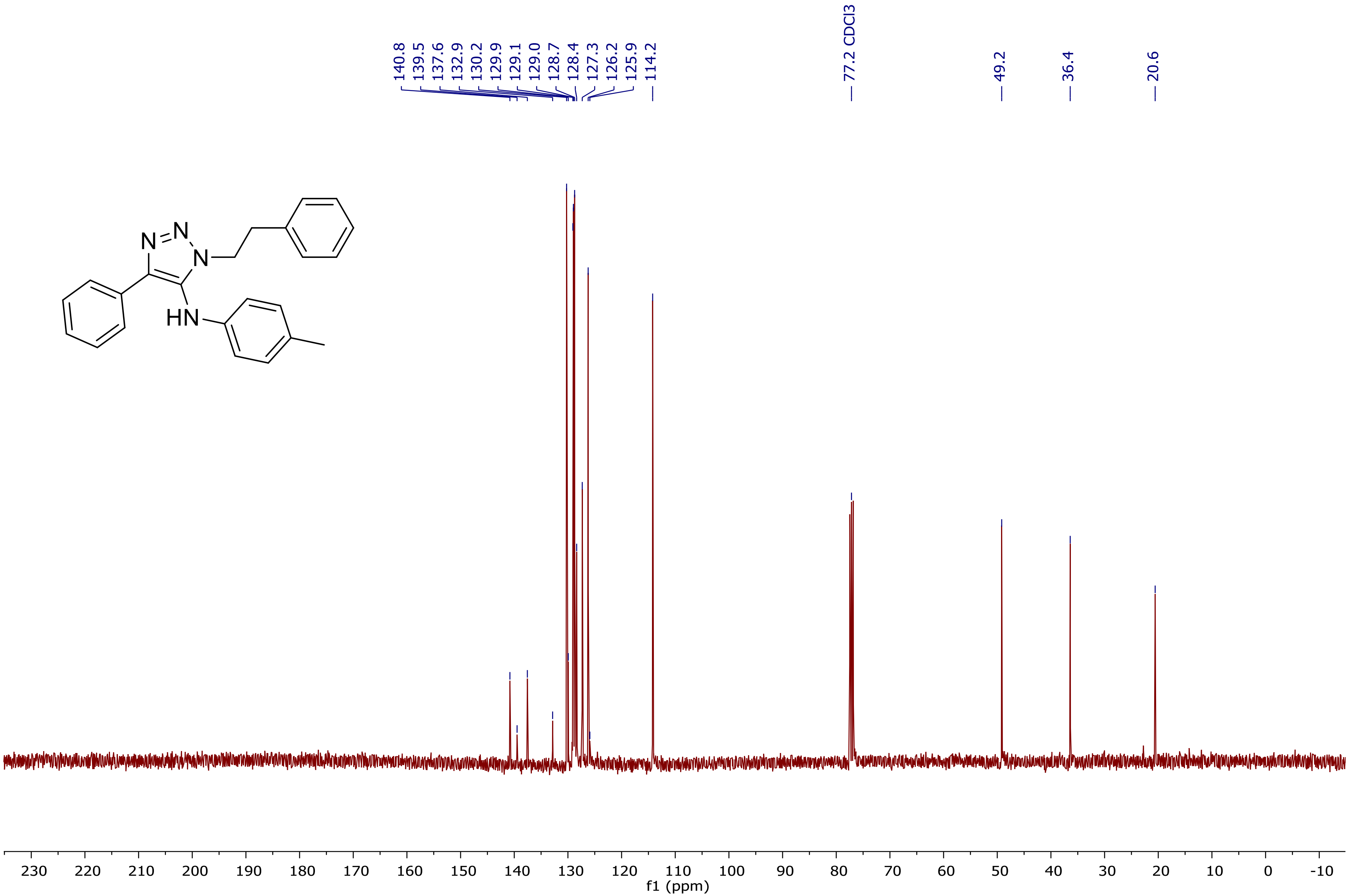

Figure S37. $^{13}C\{^{1}H\}$ **NMR (101 MHz, Chloroform-*d*) of 2q**

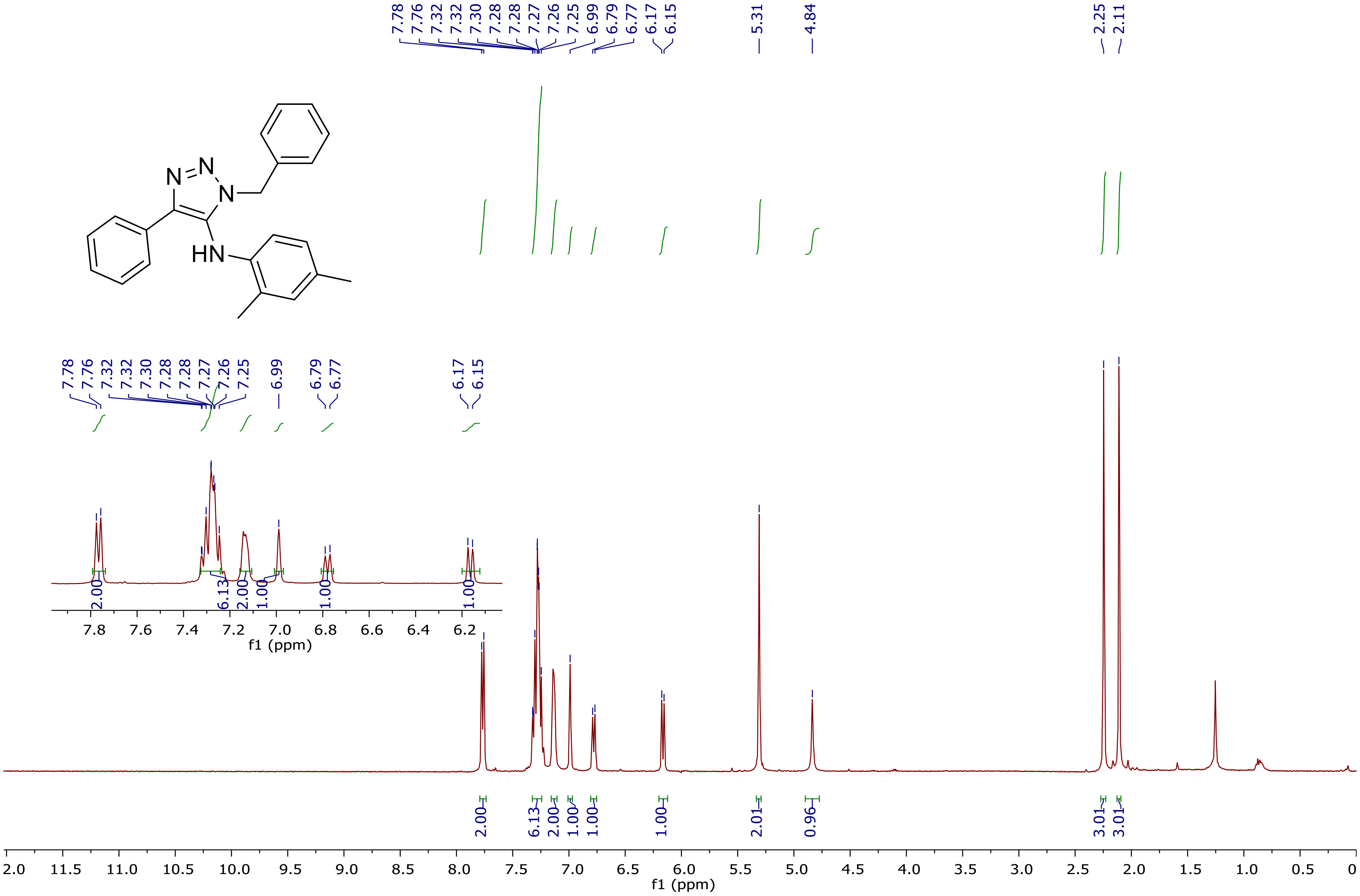

Figure S38. ¹H NMR (400 MHz, Chloroform-*d*) of 2r

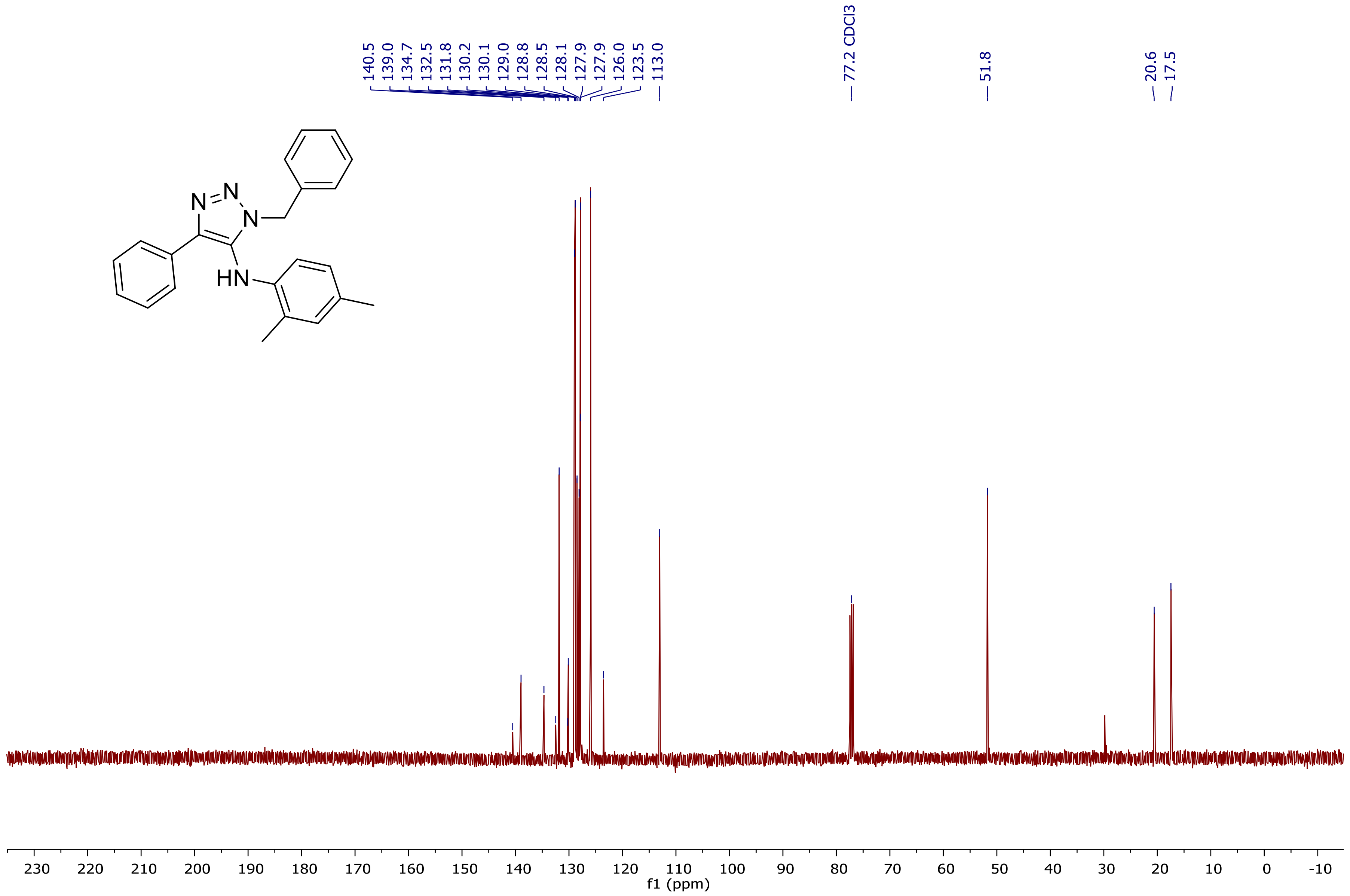

Figure S39. $^{13}C\{^{1}H\}$ NMR (101 MHz, Chloroform-*d*) of 2r

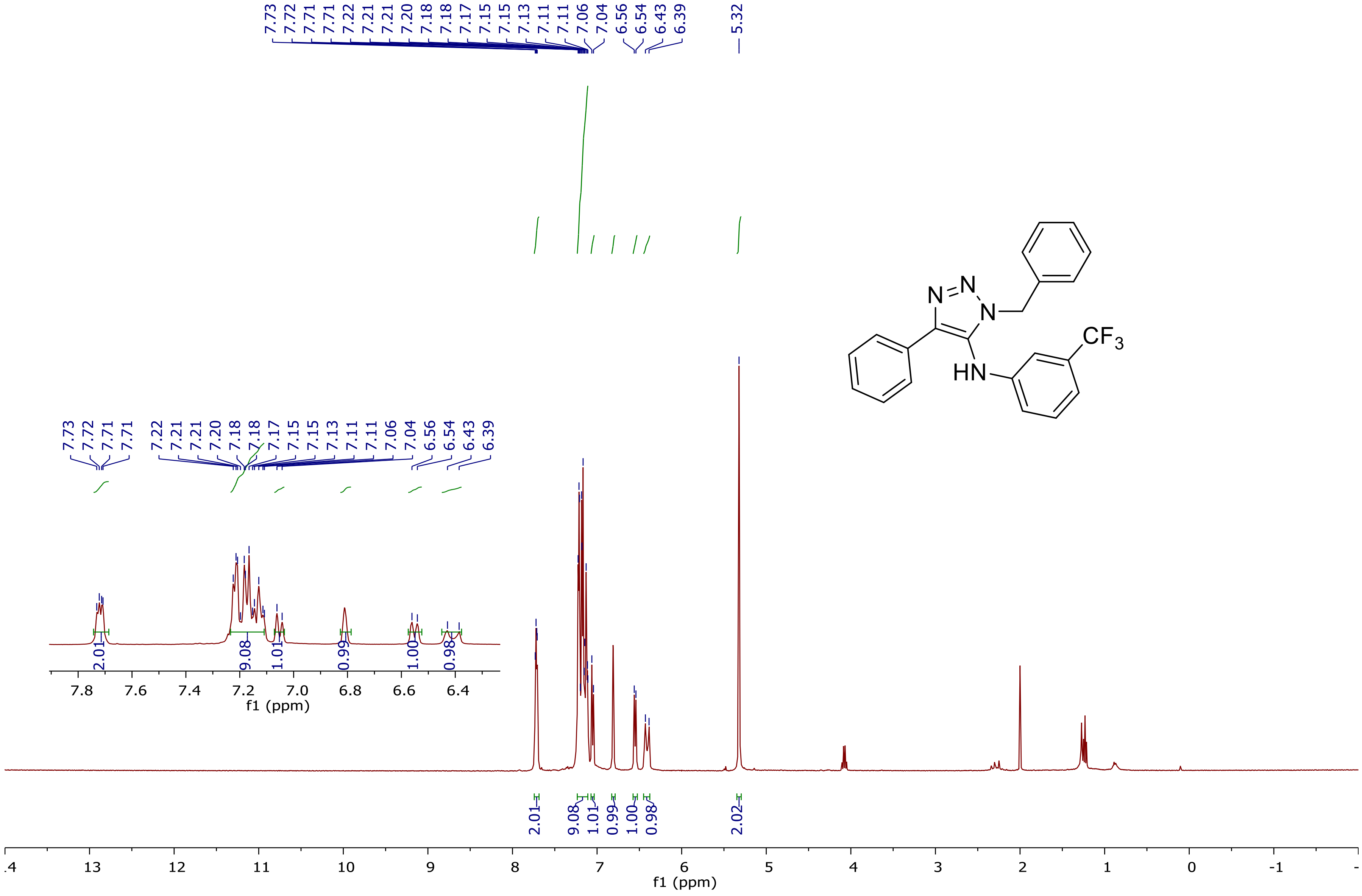

Figure S40. ^{1}H **NMR (400 MHz, Chloroform-*d*) of 2s**

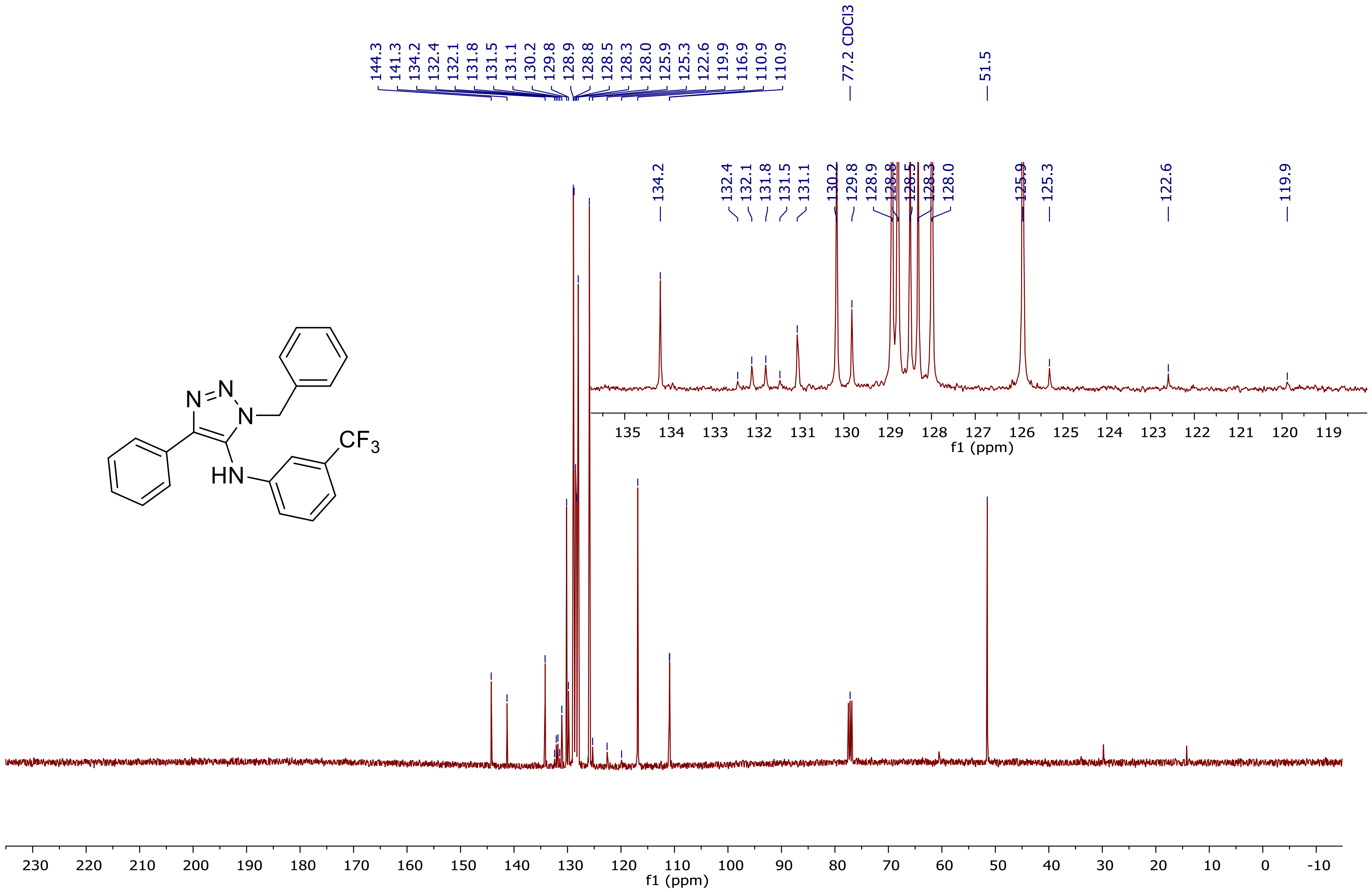

Figure S41. $^{13}C\{^{1}H\}$ **NMR (101 MHz, Chloroform-*d*) of 2s**

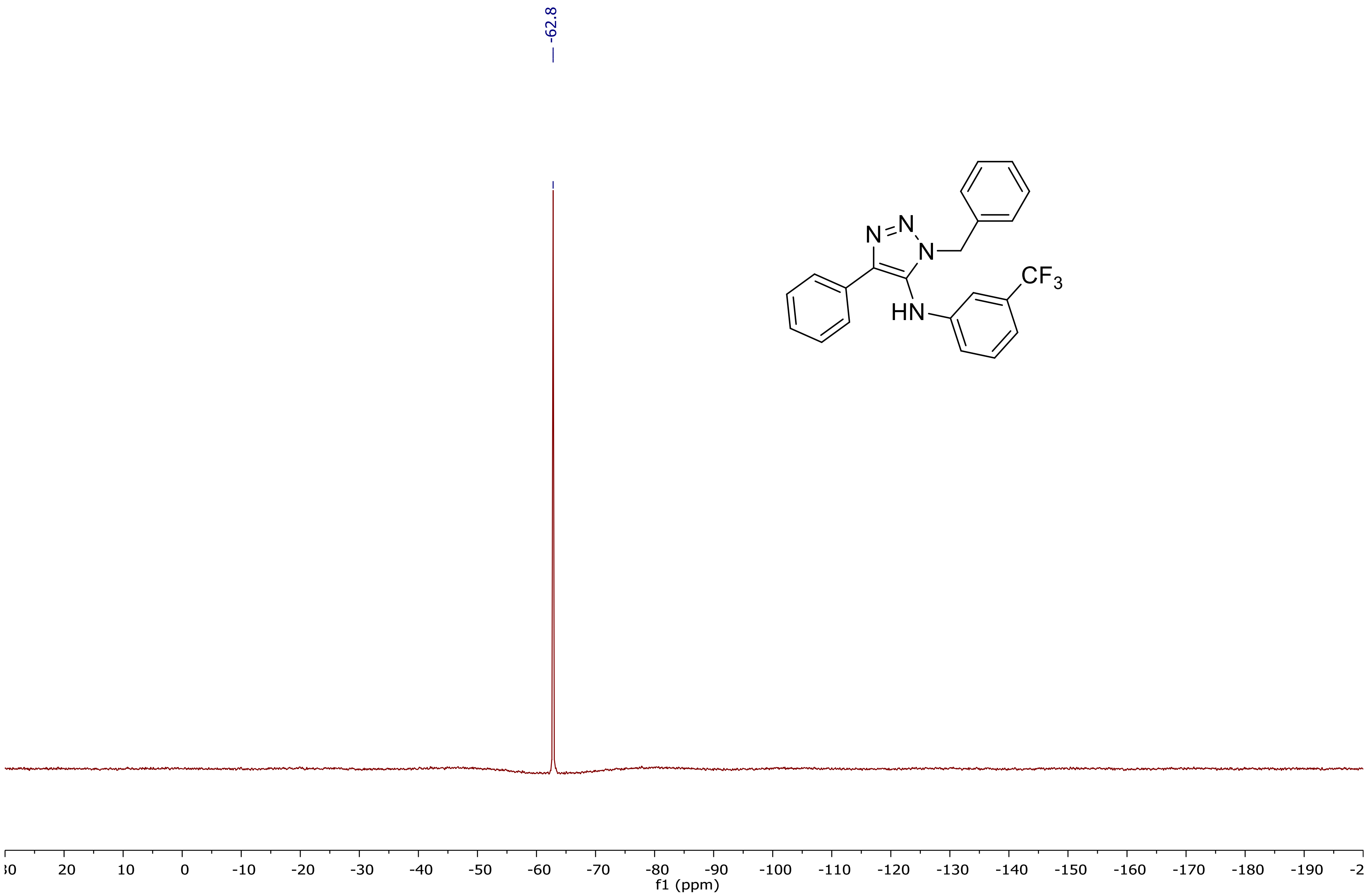

Figure S42. ^{19}**F NMR (376 MHz, Chloroform-*d*) of 2s**

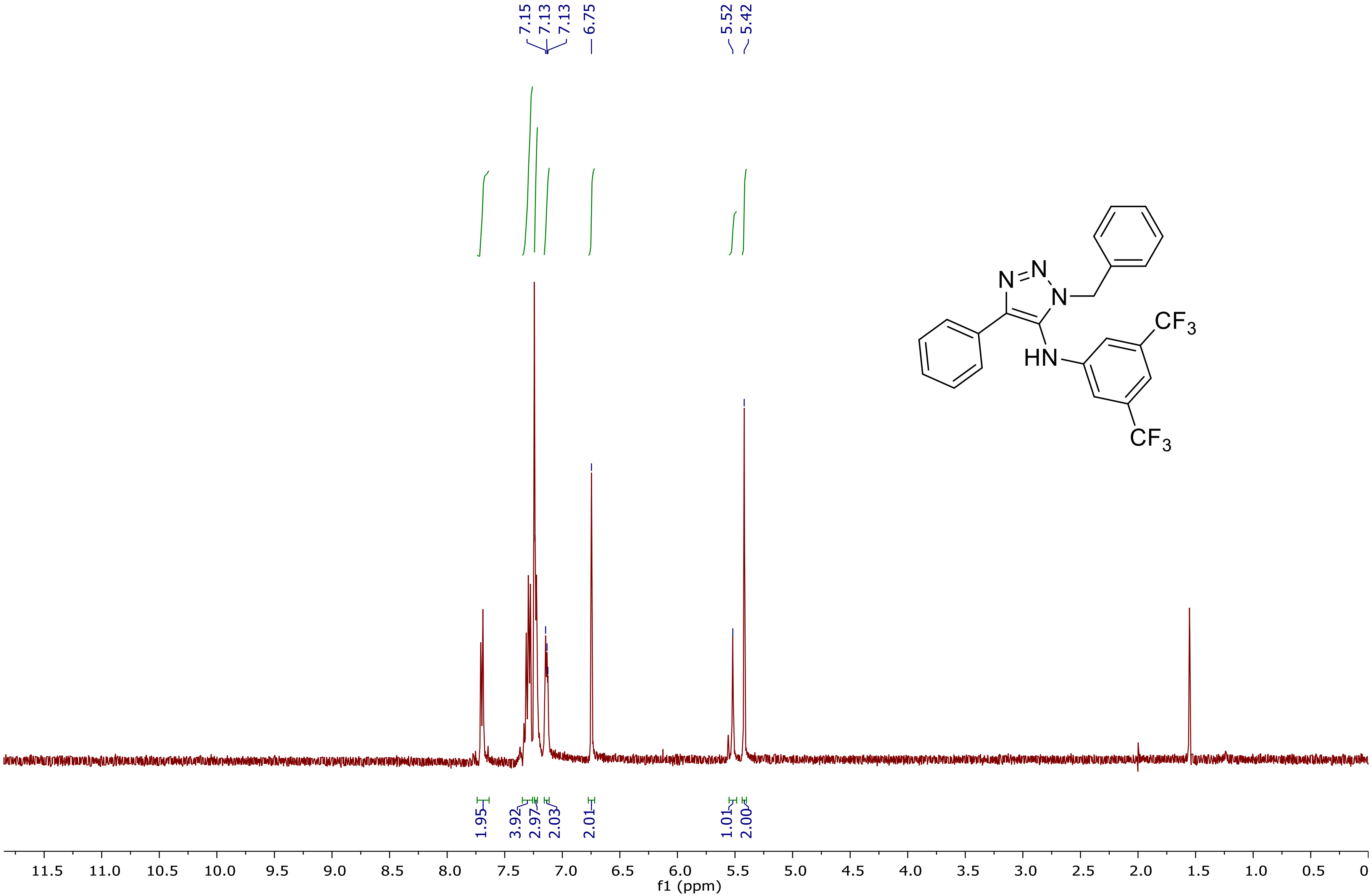

Figure S43. **^{1}H NMR (400 MHz, Chloroform-*d*) of 2t**

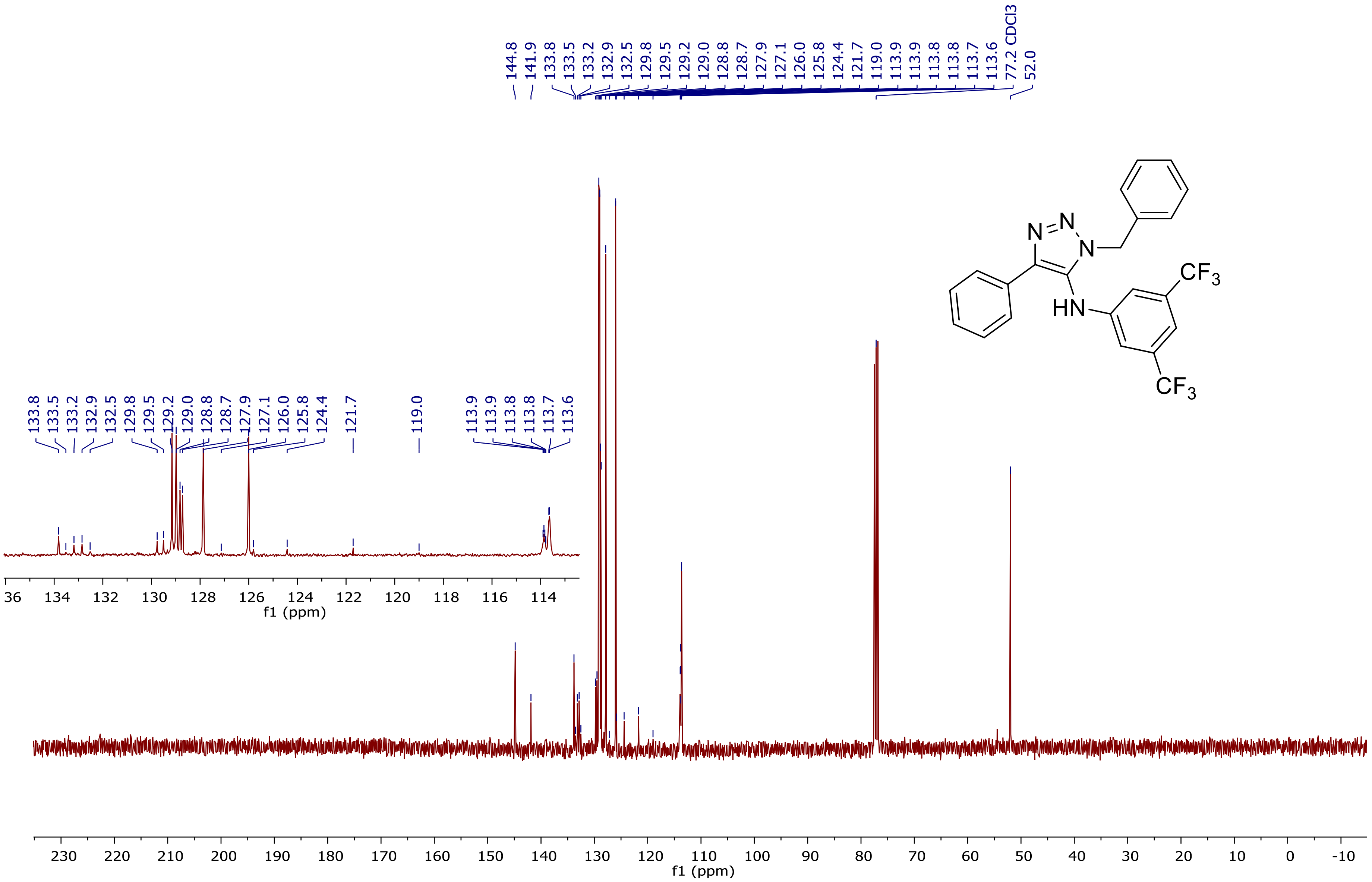

Figure S44. $^{13}C\{^{1}H\}$ **NMR (101 MHz, Chloroform-*d*) of 2t**

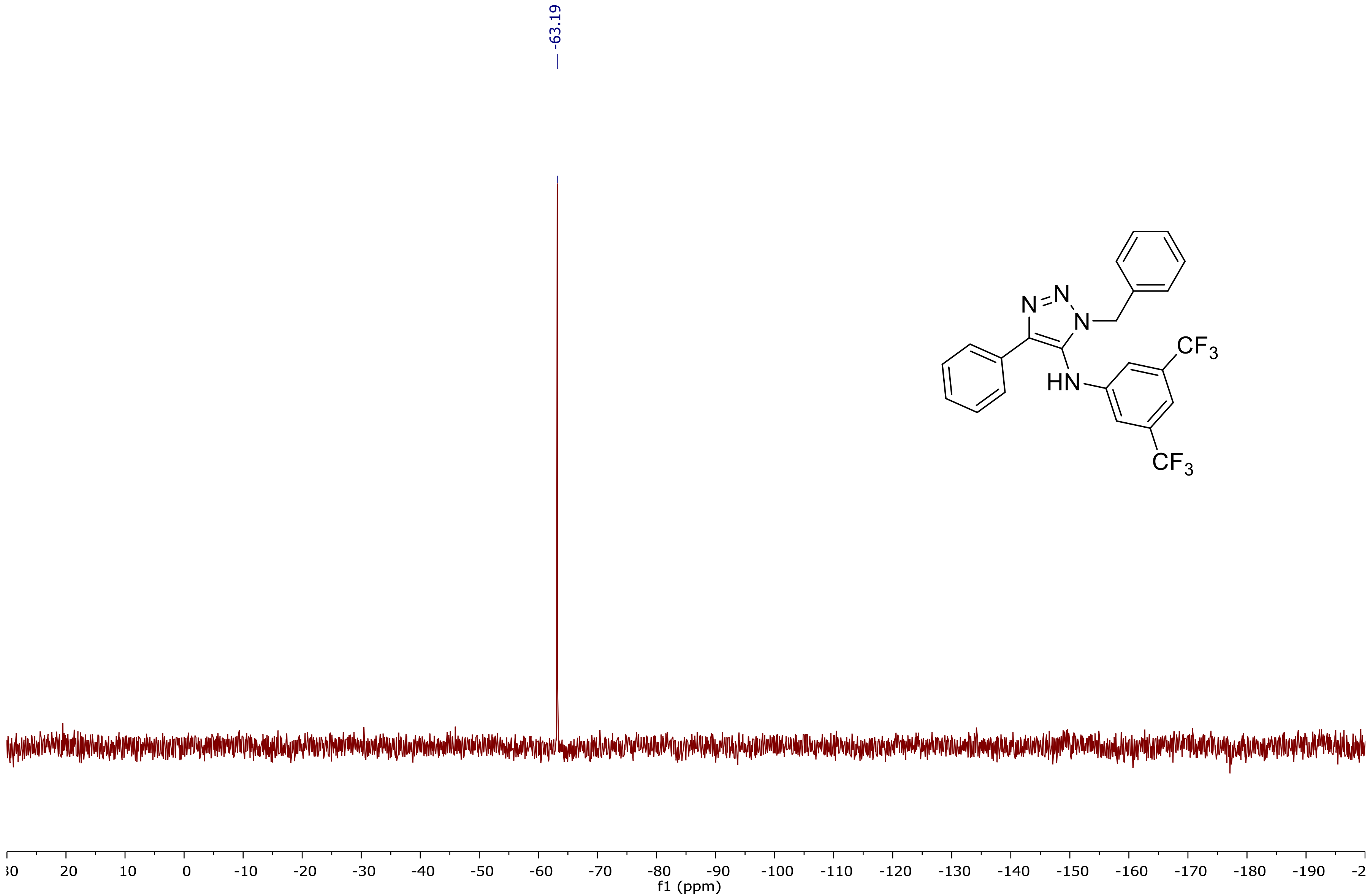

Figure S45. ^{19}F NMR (376 MHz, Chloroform-*d*) of 2t

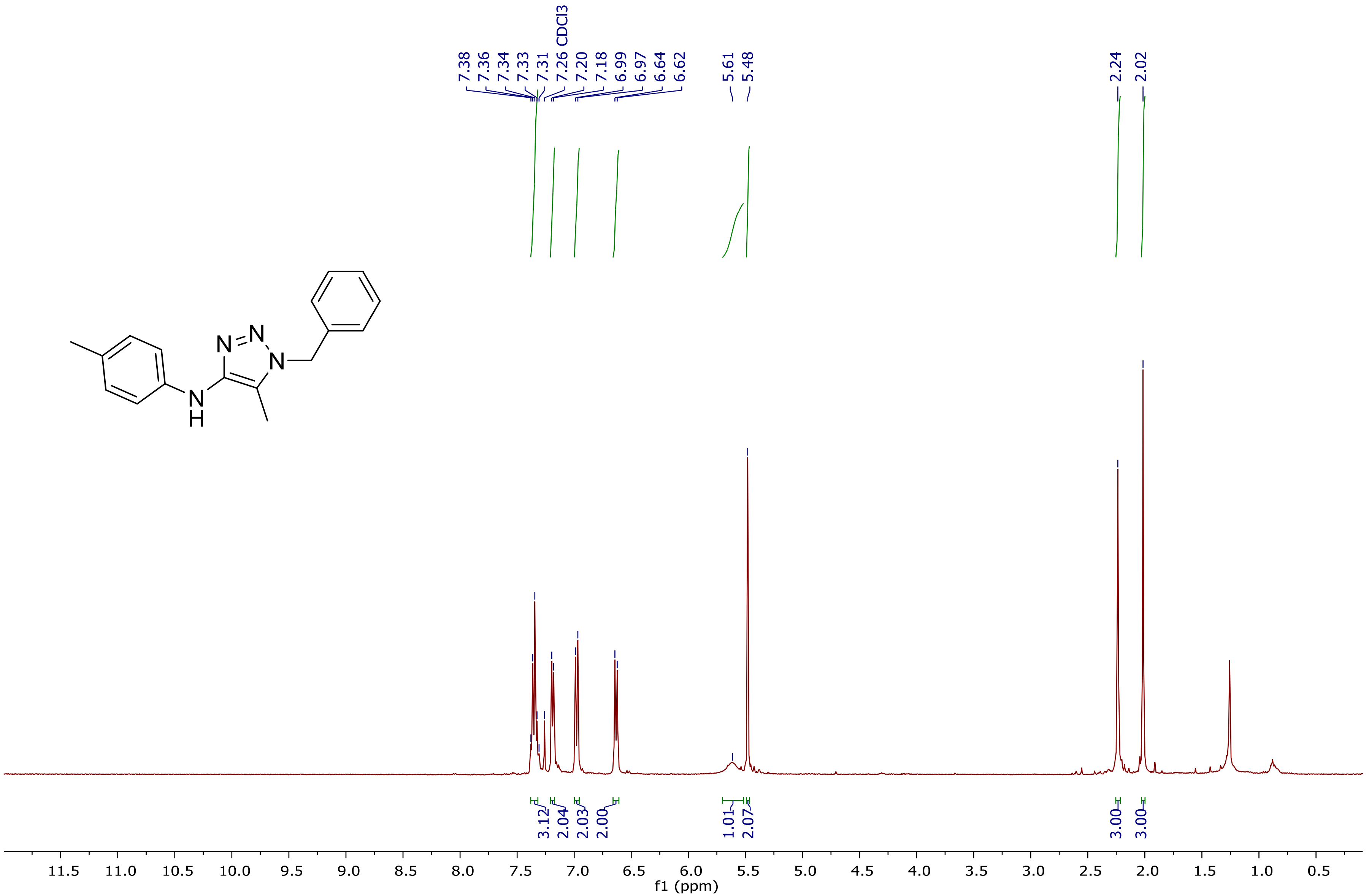

Figure S46. ^{1}H NMR (400 MHz, Chloroform-*d*) of 2u

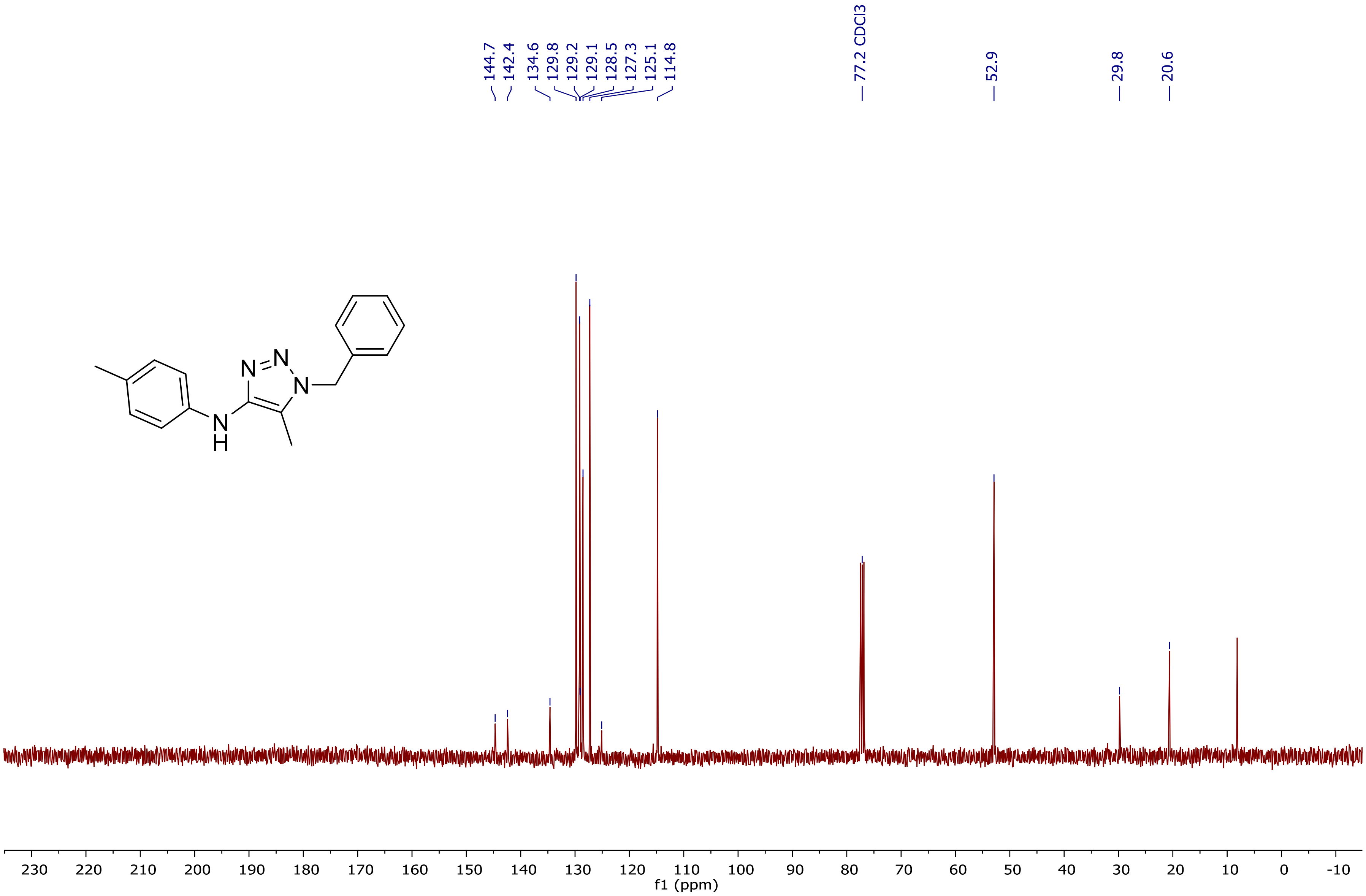

Figure S47. $^{13}C\{^{1}H\}$ **NMR (101 MHz, Chloroform-*d*) of 2u**
